# Kinome inhibition reveals a role for polo‐like kinase 1 in targeting post‐transcriptional control in cancer

**DOI:** 10.1002/1878-0261.12897

**Published:** 2021-02-01

**Authors:** Qamraa H. Al‐Qahtani, Walid N. Moghrabi, Suhad Al‐Yahya, Latifa Al‐Haj, Maher Al‐Saif, Linah Mahmoud, Falah Al‐Mohanna, Norah Al‐Souhibani, Ayodele Alaiya, Edward Hitti, Khalid S. A. Khabar

**Affiliations:** ^1^ Molecular BioMedicine Program Faisal Specialist Hospital and Research Centre Riyadh King Saudi Arabia; ^2^ Department of Comparative Medicine King Faisal Specialist Hospital and Research Centre Riyadh Saudi Arabia; ^3^ Stem Cell and Tissue Engineering Program Faisal Specialist Hospital and Research Centre Riyadh King Saudi Arabia; ^4^ Present address: Department of Pharmacology and Toxicology College of Pharmacy King Saud University Riyadh 11495 Saudi Arabia

**Keywords:** AU‐rich elements, kinase inhibitors, mRNA decay, post‐transcriptional control, targeted therapy, breast cancer

## Abstract

Dysfunctions in post‐transcriptional control are observed in cancer and chronic inflammatory diseases. Here, we employed a kinome inhibitor library (*n* = 378) in a reporter system selective for 3′‐untranslated region–AU‐rich elements (ARE). Fifteen inhibitors reduced the ARE‐reporter activity; among the targets is the polo‐like kinase 1 (PLK1). RNA‐seq experiments demonstrated that the PLK1 inhibitor, volasertib, reduces the expression of cytokine and cell growth ARE mRNAs. PLK1 inhibition caused accelerated mRNA decay in cancer cells and was associated with reduced phosphorylation and stability of the mRNA decay‐promoting protein, tristetraprolin (ZFP36/TTP). Ectopic expression of PLK1 increased abundance and stability of high molecular weight of ZFP36/TTP likely of the phosphorylated form. PLK1 effect was associated with the MAPK‐MK2 pathway, a major regulator of ARE‐mRNA stability, as evident from MK2 inhibition, in vitro phosphorylation, and knockout experiments. Mutational analysis demonstrates that TTP serine 186 is a target for PLK1 effect. Treatment of mice with the PLK1 inhibitor reduced both ZFP36/TTP phosphorylation in xenograft tumor tissues, and the tumor size. In cancer patients' tissues, PLK1/ARE‐regulated gene cluster was overexpressed in solid tumors and associated with poor survival. The data showed that PLK1‐mediated post‐transcriptional aberration could be a therapeutic target.

## Graphical abstract

A kinome inhibitor screen identified several kinases operating in post‐transcriptional control. Among those is polo‐like kinase 1 (PLK1), which is overexpressed in cancer, leading to MAPK‐activated protein kinases 2 (MK2)‐dependent phosphorylation and inactivation of the mRNA decay‐promoting protein, tristetraprolin. These events cause overexpression of many cancer genes; thus, therapeutic targeting of PLK1 kinase may restore post‐transcriptional defects in disease.

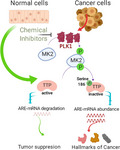

**Abbreviations**
3'‐UTR: 3' ‐untranslated region
ANOVA: analysis of variance
ARE: AU‐rich elements
B‐raf: serine/threonine–protein kinase B‐raf
CCNE2: cyclin E2
CDC6: CDC6 cell division cycle 6
CHX: cycloheximide
CIP: calf‐intestinal alkaline phosphatase
c‐MET: MET proto‐oncogene receptor tyrosine kinase
CXCL1: C‐X‐C motif chemokine ligand 1
CXCL8: C‐X‐C motif chemokine ligand 8
DMSO: dimethyl sulfoxide
E2F1: E2F transcription factor 1
E2F8: E2F transcription factor 8
ELISA: enzyme‐linked immunosorbent assay
GAPDH: glyceraldehyde 3‐phosphate dehydrogenase
G2/M: second growth phase/mitosis
H3: histone 3
HA: hemagglutinin tag
IL‐6: interleukin‐6
IL‐8: interleukin‐8
IL11: interleukin‐11
KO MEFs: knockout mouse embryo fibroblasts
LRRCC1: leucine‐rich repeat and coiled‐coil centrosomal protein
MCM10: minichromosome maintenance 10 replication initiation factor
Nluc: nanoluciferase
MAPK: mitogen‐activated protein kinase
MK2: MAPK‐activated protein kinase 2
mRNA: messenger RNA
PLAU: plasminogen activator urokinase
PLAUR: plasminogen activator urokinase receptor
PLK1: polo‐like kinase 1
RAD54L: DNA repair and recombination protein RAD54‐like
RFS: relapse‐free survival
RNA‐seq: RNA sequencing
RPS30: ribosomal S30
RTKN2: Rhotekin 2
SGFP: super enhanced green fluorescent protein
SSMD: strictly standardized mean difference
TCGA: the Cancer Genome Atlas
TMEM184A: transmembrane protein 184A
TNF: tumor necrosis factor
TTP: tristetraprolin
VEGFR2: vascular endothelial growth factor receptor 2
ZFP36: zinc finger‐binding protein 36 homolog
ZFP36L1: ZFP36 protein‐like 1

## 1 Introduction

Post‐transcriptional regulation includes mRNA decay mechanisms that fine‐tune the abundance of the mRNA available for translation. Among the well‐studied *cis*‐acting mRNA instability determinants are (AU)‐rich elements (ARE) [1, 2]. A key RNA‐binding protein that promotes ARE‐mRNA deadenylation and decay is the zinc finger protein, tristetraprolin (ZFP36/TTP). Many human tumors are found to be associated with deficiency of this protein [3, 4], which is consequently linked to hallmarks of cancer [1, 5]. The aberrant expression or activity of ZFP36/TTP could be attributed to changes at different levels of regulation, including transcriptional (e.g., epigenetic), post‐transcriptional, and post‐translational. Phosphorylation of ZFP36/TTP by several protein kinases is one of the post‐translational modifications that profoundly affect its cellular localization and activity [6, 7, 8, 9]. Given that phosphorylated ZFP36/TTP is unable to promote ARE‐mRNA decay, abundance of the proteins involved in inflammation and cancer is increased and prolonged. Thus, knowledge about the events regulating ZFP36/TTP phosphorylation in cancer is important for further understanding of targeting this pathway.

Protein phosphorylation and dephosphorylation events are mediated through the action of protein kinases, which are opposed by phosphatases [10, 11]. Nearly 2% of the human genome encodes protein kinases (http://kinase.com/kinbase/). Most of the kinase inhibitor drugs that are currently approved or in clinical trials are intended for the treatment of cancers [12, 13]. In our study, we employed a kinase inhibitor library that comprises 378 drugs containing a proportion of FDA approved agents, in addition to many in clinical trials at different phases. We used this library with an optimized reporter assay that was designed to identify the hits affecting ARE‐mediated post‐transcriptional regulation. Compounds from the library were scored as hits if they significantly reduced the expression of the reporter activity. As a result, we have identified a putative novel pathway in ARE‐mediated gene post‐transcriptional regulation involving polo‐like kinase 1 (PLK1) and studied its role in ZFP36/TTP phosphorylation.

PLK1 is a member of a family of regulatory serine/threonine kinases, which consists of five members (PLK1‐5). They are involved in the cell cycle at different levels, including mitosis, spindle formation, cytokinesis, and meiosis [14]. In our study, PLK1 was found to impart aberrant post‐transcriptional regulation of cancer‐associated ARE‐coding genes that are associated with increased abundance and stability of ZFP36/TTP, likely due to increased phosphorylation. We demonstrated that the pharmacological PLK1 inhibitor, volasertib, attenuates ZFP36/TTP phosphorylation both in vitro and in vivo, at least partially through mitogen‐activated protein kinase (MAPK) p38/MAPK‐activated protein kinase 2 (MK2). Further, PLK1 inhibitor resulted in reduction in the half‐life of ARE mRNAs. Thus, PLK1 inhibition normalized aberrant ZFP36/TTP activity in cancer cells, making PLK1‐mediated post‐transcriptional pathway and ZFP36/TTP phosphorylation promising targets for cancer therapy.

## 2 Materials and methods

### 2.1 Cell lines

Breast cancer cell lines MDA‐MB‐231, SKBR3, MCF‐7, the normal‐like breast cell line MCF10A, and the HEK293 kidney cell line were obtained from American Type Culture Collection (ATCC, Rockville, MD, USA). MK2‐knockout mouse embryo fibroblasts (MEF) were kindly provided by Dr. M. Gaestel (Germany) [15]. HEK293, MEFs, MDA‐MB‐231, and MCF‐7 cell lines were cultured in Dulbecco's modified Eagle's medium (DMEM; Invitrogen, Carlsbad, CA, USA) at 37 ^ο^C supplemented with 2 mm glutamine and 10% fetal bovine serum (FBS). The SKBR3 cell line was cultured in McCoy's 5A Medium (McCoy's 5A; Thermo Fisher Scientific, Waltham, MA, USA) supplemented with 10% FBS, whereas, MCF10A cells were maintained in Ham's F12–DMEM mixture (Thermo Fisher Scientific, Waltham, MA, USA) and supplemented with 20 ng·mL^−1^ epidermal growth factor (EGF), 0.01 mg·mL^−1^ bovine insulin, and 500 ng·mL^−1^ hydrocortisone (Sigma, St. Louis, MO, USA). All culture media were supplemented with 1% penicillin–streptomycin antibiotics (Sigma‐Aldrich).

### 2.2 Protein kinase inhibitor library and other compounds

Selleckchem Kinase Inhibitor Library that includes 378 pharmacologically active inhibitors of many protein kinases was purchased from Selleckchem (Houston, TX, USA). Each compound is provided as a predissolved 10 mm dimethyl sulfoxide (DMSO) solution in 96‐well format tubes and diluted to working solution, first as 1 mm DMSO, then in the experiment medium. The MK2 inhibitor, PF‐364402, was purchased from Tocris (Tocris Bioscience, Minneapolis, MN).

### 2.3 Plasmids and transfections

PLK1 expression vector was obtained from GeneCopoeia (Rockville, Maryland, US); vector expressing human hemagglutinin (HA)‐tagged ZFP36 (TTP) was described in Ref.[16], and HA‐tagged ZFPL36L1 (BRF1) was cloned by PCR from cDNA in a CMV‐driven expression vector. The mouse TTP wild‐type (WT) and TTP‐S52/178A mutant were originated from G. Stoecklin and were used previously [17, 18]. Human ZFP36/TTP expression vectors were subjected to site‐directed mutagenesis to produce serine to alanine substitution in 60 and 186 sites. All plasmids were verified by our sequencing facility. All transfections were performed in reduced serum media using either Lipofectamine 2000 or Lipofectamine LTX (Invitrogen, Carlsbad, CA, USA). SuperGFP expression plasmid cotransfection was used to monitor transfection effect.

### 2.4 Post‐transcriptional reporter assay

The RPS30 promoter (RPS30)‐linked SuperNanoluciferase (SuperNluc) expression vectors comprised either control non‐ARE‐coding 3′UTR or ARE‐coding 3′UTR containing clustered copies of AUUUA and were previously described [19, 20]. The constructs (2 µg) were transfected to MDA‐MB‐231 cells in 10‐cm^2^ plates and then seeded into 96‐microwell plates at a density of 4 × 10^4^ cells/well and incubated overnight. The cells were transfected using the Lipofectamine 2000. The superluciferase (firefly) was used as a normalization control as needed (i.e., when using different vectors). After 24 h, the cells were treated with each member of protein kinase inhibitors for 24 h. Then, luciferase reaction was performed as prescribed by manufacture's protocol (Nano‐Glo Luciferase, Promega, Madison, WI, USA)/well and measured through chemiluminescence using a Zenith 3100 (Anthos Labtec, Eugendorf, Austria).

### 2.5 Quantitative reverse transcription–polymerase chain reaction and mRNA half‐life

Total RNA was extracted using TRIzol reagent (TRI Reagent, Sigma‐Aldrich). The cells were lysed directly on the culture dish by adding 1 ml of the TRI Reagent per 10‐cm^2^ surface area. Reverse transcription for preparation of cDNA was performed using 3 μg of total RNA, 150 ng random primers, 0.1 M dithiothreitol (DTT), 10 mm deoxynucleotide triphosphate (dNTP), and 200 U of SuperScript II (Invitrogen, Foster City, CA). The quantitative RT–qPCR was performed in multiplex in the Chroma 4 DNA Engine cycler (Bio‐Rad, Hercules, CA, USA) using FAM‐labeled TaqMan probes (Applied Biosystems, Foster City, CA, USA) for uPA (PLAU ), IL‐8, PLK1, and SNluc, while a VIC‐labeled glyceraldehyde‐3‐phosphate dehydrogenase (GAPDH) probe was used as the endogenous control. Samples were amplified in triplicate, and quantification of relative expression was performed using the estimation of quantitation cycle (Cq) method.

For the mRNA half‐life experiments, 5 μg·mL^−1^ of actinomycin D (ActD; Sigma‐Aldrich, St Louis, MO) was added to the cells for 1, 2, 4, and 6 h prior to extraction of total RNA using TRIzol reagent (Sigma). The reverse transcription reaction and quantitative PCR were performed as described above. The half‐life of mRNAs was estimated using the one‐phase exponential decay method, using GraphPad Prism software (GraphPad Software, San Diego, CA).

### 2.6 RNA‐seq experiments

MDA‐MB‐231 cells were treated with either DMSO or volasertib (330 nm) overnight. Then, the cells were dissolved in TRIzol reagent. Briefly, mRNA was purified from total RNA using oligo(dT)‐attached magnetic beads. The mRNA molecules were fragmented, and cDNA was generated using random hexamer‐primed RT, followed by a second‐strand cDNA synthesis. The synthesized cDNA was subjected to end‐repair and was then 3′ adenylated. Adapters were ligated to the ends of these 3′ adenylated cDNA fragments. The cDNA fragments with adapters were amplified using PCR, and then purified with Ampure XP Beads (Agencourt). The resultant library was validated on the Agilent Technologies 2100 bioanalyzer. The double‐stranded PCR products were heat‐denatured and circularized by the splint oligo sequence. The library was amplified with phi29 to make DNA nanoballs and loaded into the patterned nanoarray, and single‐end 50 (pair end 100) base reads were generated by sequencing. Transcriptome sequencing was performed using Illumina HiSeq2000 at BGI (Shenzhen, China). After sequencing, quality control and filtering of raw data were performed, in which low‐quality reads were removed. Clean reads were stored in FASTQ format after filtering and were used for downstream bioinformatics analysis, including differential gene expression and functional enrichment using DAVID bioinformatics.

### 2.7 Protein stability assay

MDA‐MB‐231 cells were seeded overnight and then treated with CHX (Sigma‐Aldrich) at a concentration of 30 µg·mL^−1^ for 0, 1, 2, 4, and 6 h. Cells were collected, and the protein levels were determined by western blotting. For cotransfection experiments, HEK293 cells were transfected with either ZFP36/TTP (0.3 µg/million cells) and empty vector (0.2 µg/million cells) or ZFP36/TTP and PLK1 expression vectors together for overnight, and then treated with CHX as above. Cells were collected, the protein abundance was determined by western blotting, and the quantification was performed using ImageJ software. The protein half‐life was determined using regression analysis with GraphPad software.

### 2.8 Western blotting and TTP phosphorylation

Cells were lysed in a mixture of 0.5% NP‐40 buffer, protease inhibitor, and phosphatase inhibitor. The calf‐intestinal alkaline phosphatase (CIP, Promega, Madison, WI, USA) was used to verify the phosphorylation status of ZFP36/TTP; 20 units were added to the cell lysate (per 250 ul). The cell lysates were loaded and subjected to electrophoresis on 4‐12% NuPAGE Bis–Tris gel (Invitrogen, Foster City, CA, USA). Then, the proteins were transferred from the gel to nitrocellulose membranes (Hybond ECL; Amersham Biosciences, Piscataway, NJ) in the presence of NuPAGE 20 x transfer buffer (Invitrogen). After blocking, membranes were incubated with the primary antibodies diluted in 5% bovine serum albumin (BSA) (Sigma‐Aldrich, St Louis, MO) at 4˚C overnight. For ZFP36/TTP, a custom‐made affinity‐purified TTP polyclonal antibody was raised against the C‐terminal end of TTP [17]. This antibody is specific to ZFP36/TTP, but not the ortholog ZFP36L1 when using 0.5% NP‐40 buffer instead of Laemmli buffer. Other antibodies used are as follows: anti‐PLK1 (dilution 1:1000, Cell signaling, Massachusetts, USA), anti‐MK2 and anti‐phospho MK2 antibody (Cell Signaling), anti‐uPA (dilution 1:1000, Cell signaling; anti‐actin (dilution 1:1000, Cell signaling), anti‐GAPDH (dilution 1:500, Abcam, MA, USA), and anti‐HA (dilution 1:5000, Roche, Upper Bavaria, Germany). Thereafter, the membranes were incubated with corresponding secondary antibodies (diluted in 5% BSA, 1:2000 dilution) (Santa Cruz Biotech, Santa Cruz, CA) for 1–3 h. Protein bands were detected using ECL western blotting detection reagents (Amersham Biosciences, Amersham, UK) in Molecular Imager ChemiDoc machine (Bio‐Rad, Hercules, CA, USA).

### 2.9 In vitro PLK1 kinase assay

The kinase reaction was performed with active human recombinant PLK1 (100 ng) and combined with the substrate either dephosphorylated casein (100 ng, positive control) or inactive MK2 (100 ng; Sigma, Cat No. 14‐349) using 1 X reaction buffer (40 mm Tris, 7.5 pH; 20 mm MgCl_2_, 0.1mg·mL^−1^ BSA, 50 μM DTT) and 50 µm ATP. The reaction was incubated for 60 min. The generated ADP from ATP during the kinase reaction was measured using PLK1 ADP‐Glo Kinase assay kit (Promega) according to the manufacturer's instruction. Briefly, 25 µL of ADP‐Glo reagent was added to the 25‐µL reaction and incubated for 40 min. Subsequently, the detection reagent was added, and the mixture was incubated for 30 minutes. All reactions were performed at room temperature. Luminescence signal was recorded using the multimode Varioskan Flash reader (Thermo Fisher Scientific). Data are blotted as relative luminescence unites.

### 2.10 Cell cycle analysis

Subconfluent cells (in growing phase) were treated with DMSO, volasertib (330 nm), or nocodazole (50 ng·mL^−1^) for 8 or 24 h. The drug concentrations were comparable in cell cycle arrest at G2/M phase. For flow cytometry, the cells were collected, fixed in ice‐cold 70% ethanol and washed in 1% PBS. The cells were then incubated at room temperature for 30 min. with 200 µg·mL^−1^ RNase A (Sigma) followed by staining with 50 µg·mL^−1^ propidium iodine (Calbiochem, San Diego). Cell cycle fractions were assessed by flow cytometry using FACSCalibur instrument and software (Becton Dickinson). For immunoblotting, proteins were extracted from the cells, and western blotting using rabbit anti‐phosphorylated histone H3 (Ser 10, Cell Signaling) was used.

### 2.11 Animal studies

Nude mice were purchased from Jackson Laboratories (Bar Harbor, Maine, USA). The mice were housed at the animal facility at King Faisal Specialist Hospital and Research Center (KFSHRC) and maintained in accordance with protocols approved by the institution’s Animal Care and Use Committee (ACUC). The experiments and the procedures were approved by ACUC‐KFSHRC of the Research Advisory Council (RAC# 2110 032). One million MDA‐MB‐231 cells were suspended in 100 µL of PBS: Matrigel (1:1 ratio), then injected into the 4th mammary fat pad of female mice (8 weeks old) after they were anesthetized with isoflurane. Tumors were allowed to grow for one week before treatment with Volasertib (10 mg·kg^−1^) or DMSO alone (*n* = 5 mice per group). Treatment was administered via intraperitoneal route twice a week for four weeks after which mice were sacrificed and tumors collected for RNA and protein analyses. Tumor growth was measured by caliper, and the volume was calculated by the formula (π/6 x L x M2), where L and M refer to the large and small diameters of each tumor, respectively. Mice weight and tumor size were recorded weekly.

### 2.12 Patient data and analysis

The Cancer Genome Atlas (TCGA) was searched using the Oncomine web portal, www.oncomine.com or www.nextbio.com. TCGA has stated the following: 'All samples in TCGA have been collected and utilized following strict human subjects protection guidelines, informed consent, and IRB review of protocols'. The patients' survival data were generated using GOBO breast cancer or www.kmplot.com portals.

### 2.13 Image and statistical analysis

Data are presented as mean ± standard error of the mean (SEM). Images from blots were converted to 8‐bit gray scale images and analyzed by ImageJ software (NIH). The pixels were measured using median intensity readings after subtracting local background. Normalized ratios were performed using GAPDH image data. Two‐tailed Student's t‐test was used when comparing two columns of data. One‐way analysis of variance (ANOVA) was used to analyze multiple columns, and two‐way ANOVA was used for two groups of data (each having two data columns) with post hoc Tukey's or Sidak's comparison tests. The analyses were performed using GraphPad Prism. For high‐throughput screening hit selection (protein kinase inhibitors), strictly standardized mean difference (SSMD, β) test was used [21]. This statistical test measures the size of the effect of each member in a group relative to the other members. Based on SSMD values, the effect of each member of the library was classified as strong (β ≥ 5), moderate (1 ≤ β<5), and weak (β < 1). In this study, only drugs with strong β were selected from the primary screening.

#### 2.13.1 Results

### 2.14 Kinome inhibitor screening for ARE‐mediated post‐transcriptional regulation

**Fig. 1:**
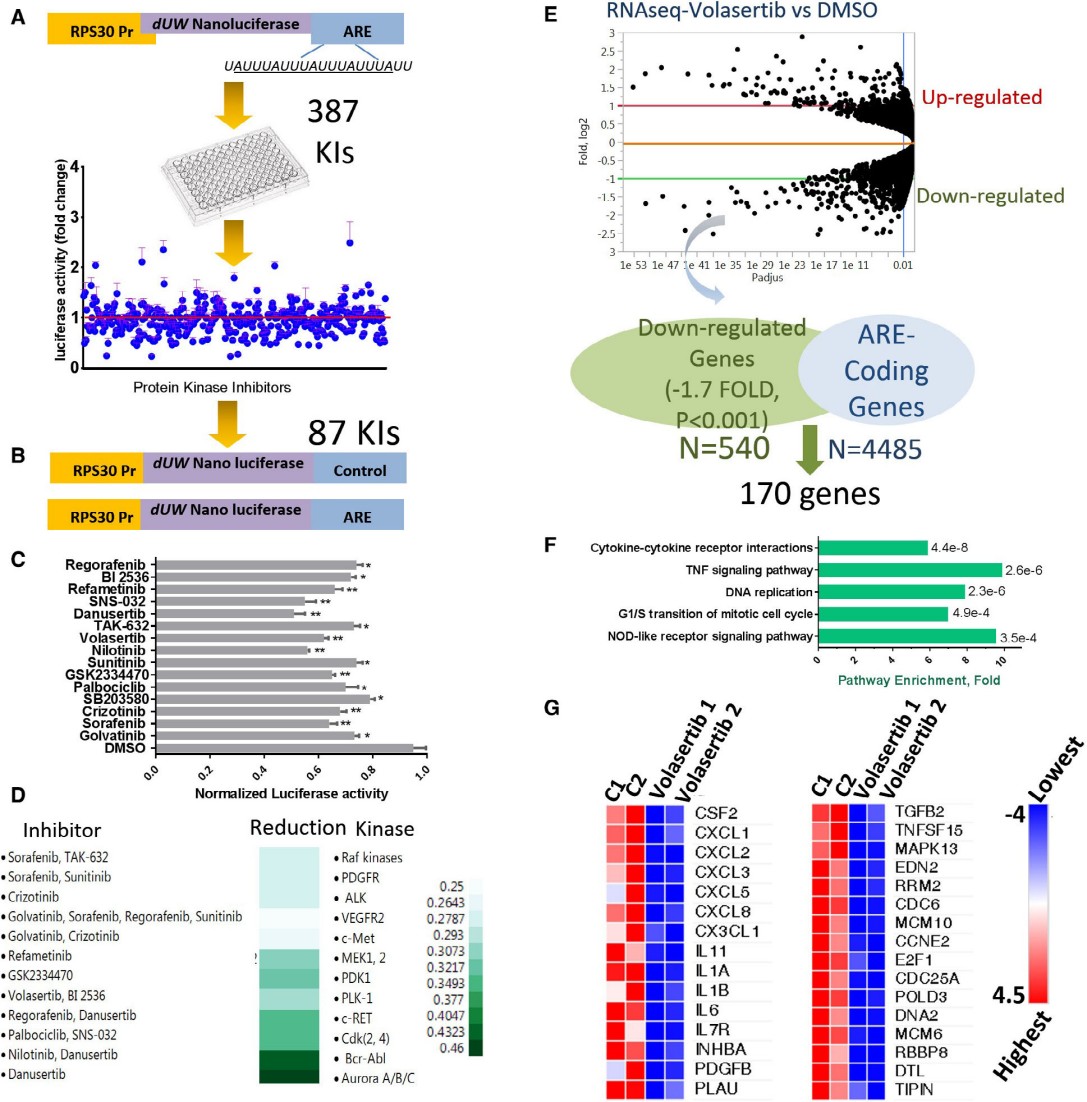
Kinome inhibitors screening on post‐transcriptional gene regulation. (A) Primary screening of 378 kinase inhibitors; MDA‐MB‐231 cells were transfected with ARE‐3′UTR fused Nluc reporter and then treated with each kinase inhibitor (5 µm) for 16 h; subsequently, the reporter's activity was measured. The mean and SEM of six readings (of two independent screens) were calculated for each drug and scattered around the control DMSO (red line). Using the SSMD statistical test, 87 drugs were found to significantly reduce the activity of ARE reporter. (B) Secondary screening of 87 kinase inhibitors, the cells were transfected with ARE‐ and non‐ARE control Nluc reporters and treated with selected agents (0.5 µm). Out of these, 15 agents (C) yielded specific reduction in ARE activity (mean ± SEM, triplicate readings of one experiment of two). (D) Heat map illustrates proportion reduction in ARE bearing reporter activity, as demonstrated by the gradual intensity of the green color (JMP software). (E) RNA‐seq analysis for PLK1 inhibitor effects on mRNA expression: MDA‐MB‐231 cells were treated with DMSO vehicle or the PLK1 inhibitor, Volasertib (0.5 log_10,_ i.e., 330 nm), total RNA was extracted, and then, RNA‐seq protocol was performed (upper panel). Data are from two independent experiments, showing average log2‐fold differential expression. The lower panel shows the intersection of PLK1 inhibitor downregulated mRNAs and ARE‐containing mRNAs (ARED). (F) Functional enrichment analysis of the resultant gene group; the analysis was performed using David bioinformatics. (G) A heatmap generated from RNA‐seq analysis showing further details of the effect of PLK1 inhibitor on the abundance of key cytokines and cellular growth genes *P* < 0.001). Scale is log2 where blue colors indicate reduction relative to red color for each gene (*P* < 0.001). [Colour figure can be viewed at wileyonlinelibrary.com]

The MDA‐MB‐231 cell line represents a model for highly invasive solid carcinomas that are characterized by aberrations in several protein kinase pathways and ARE‐mediated post‐transcriptional control [22, 23, 24]. The protein kinase inhibitors (*n* = 378) were examined for their effect on the expression of ARE‐ and non‐ARE‐containing reporters, using an optimized and selective post‐transcriptional reporter system [19]. First, a ‘primary screen’ was performed in which the ARE reporter‐expressing MDA‐MB‐231 cells were treated with 5 µm of each kinase inhibitor or DMSO as vehicle control for 16 h (Fig. 1A; Table S1). Using the SSMD statistical test, 87 drugs were found to reduce (β ≥ 5) the activity of ARE‐containing reporter significantly. A second screen was performed for the 87 drugs using an additional non‐ARE‐reporter expression construct, and a lower concentration (0.5 µm) of the kinase inhibitors (Fig. 1B; Table S2). Both approaches meant to increase the assay specificity by eliminating those drugs affecting the control non‐ARE reporter due to nonspecific cellular activities such as toxicity. Fifteen drugs with specific effects on the ARE‐mediated reporter were observed (Fig. 1C). These inhibitors target nearly 12 different kinases, including B‐Raf kinase, VEGFR2, c‐Met, ALK, MEK1, and polo‐like kinase 1 (Fig. 1D). We elected to focus on the PLK1 inhibitor, volasertib, not only because it provided consistent and significant results in the primary and secondary screens, but also because PLK1 would constitute a novel player in ARE‐mediated post‐transcriptional regulation. Moreover, PLK1 has been proven to possess multiple functions besides cell cycle such as cell–cell adhesion, invasion, epithelial‐to‐mesenchymal transition, and apoptosis [25, 26, 27]; these processes can be subjected to ARE‐mediated post‐transcriptional control [1].

### 2.15 PLK1 inhibition and global ARE‐coding gene expression

In order to gain global insights into the reduction in AU‐rich mRNA expression abundance due to PLK1 inhibition, RNA‐seq experiments were performed using MDA‐MB‐231 cell line treated with DMSO vehicle or the PLK1 inhibitor, volasertib. The expression of 540 genes was reduced by at least 1.7‐fold (*P* < 0.001; Fig. 1E) (Table S3). This group was crossed with the ARE‐mRNA database (ARED‐Plus) [28] to cluster the PLK1 inhibitor‐downregulated and ARE‐mRNA groups, which yielded 170 genes (Fig. 1E, lower panel; Table S4). Functional enrichment was performed with top categories in functions related to cell cycle and cytokine (particularly TNF) signaling (Fig. 1F,G). There was a significant reduction (*P* < 0.001) in the mRNA abundance of the pro‐inflammatory cytokines and chemokines (e.g., CXCL1, CXCL8 (also called IL‐8), IL‐1, and IL‐6) by at least 70% inhibition due to Volasertib (Fig. 1G). The transcript abundance of key cellular growth genes was reduced on average by 60% due to the treatment of the PLK1 inhibitor (Fig. 1G).

### 2.16 Reduction in ARE‐mRNA expression by the kinase inhibitor group

**Fig. 2:**
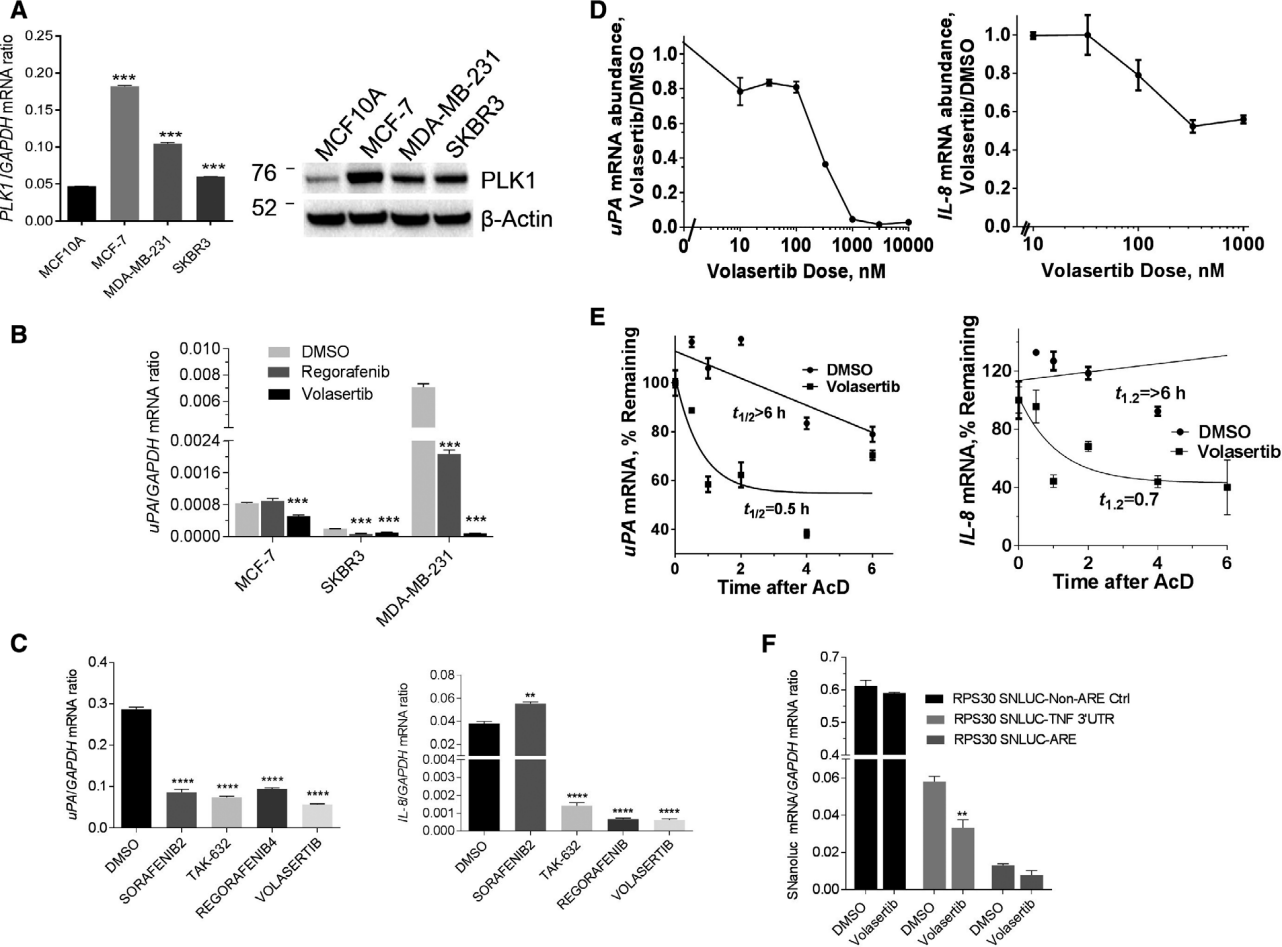
PLK1 inhibition accelerates ARE‐mRNA‐dependent decay. (A) PLK1 mRNA and protein expression in normal and breast cancer cell lines; PLK1 mRNA expression in MCF‐10A, MCF‐7, and MDA‐MB‐231 cell lines quantified by RT–qPCR using FAM‐labeled PLK1 and normalized with VIC‐labeled GAPDH probes (*left panel*). Values were expressed as mean ± SEM (three different experiments), and comparison to MCF10A control was performed by Student′s t‐test (*P < 0.05, **P < 0.001). *Right panel* shows the expression of PLK1 protein in normal and cancer cells; western blot was performed using specific antibodies to PLK1, and the housekeeping beta‐actin protein shown is a representative blot from three independent experiments. (B) PLK1 inhibition in different breast cancer cell lines. MCF7, SKBR3, and MDA‐MB‐23. Cells were treated with either DMSO, the VEGFR2 inhibitor, regorafenib (500 nm), or the PLK2 inhibitor, volasertib (330 nm) for 8 h, and then, uPA mRNA abundance was measured using RT–qPCR. (C) Effect of inhibitors of PLK1 and other kinases on ARE‐mRNA abundance. MDA‐MB‐231 cells were either treated with DMSO or the indicated inhibitors (300 nm) for 24 h. The uPA and IL‐8 mRNA abundance was determined in relation to GAPDH using RT–qPCR. The values were expressed as mean ± SEM (triplicate readings, one representative experiment of three). **P < 0.005 and ****P < 0.0005, Student′ t‐test (in comparison with DMSO control. (D) Dose–response curve of the effect of volasertib on mRNA levels of uPA and IL‐8 in MDA‐MB‐231 using RT–qPCR. The values were expressed as mean ± SEM of triplicate readings (one experiment of two independent experiments). (E) PLK1 inhibitor treatment accelerates mRNA decay. MDA‐MB‐231 cells were treated with 300 nm volasertib for 24 h, followed by treatment with 5 µg actinomycin D. Total RNA extraction followed by RT–qPCR was performed. The values were expressed as mean ± SEM of quadruplicate readings from one experiment of at least two. The half‐life determination was quantitated using one‐phase decay model. (F) The effect of volasertib on the ARE‐harboring SuperNluc reporter mRNA expression; MDA‐MB‐231 cells transfected with the indicated SuperNluc plasmid were treated with DMSO or volasertib (330 nm) for 8 h. The mRNA expression of SuperNluc was measured using quantitative RT–qPCR with specific primers. Data are mean ± SEM of replicate readings of one representative experiment of two. [Colour figure can be viewed at wileyonlinelibrary.com]

We further confirmed the effects of the PLK1 inhibitor, volasertib, on the expression of two procancer ARE‐coding genes, interleukin‐8 (IL‐8, CXCL8) and urokinase plasminogen activator, uPA (PLAU). First, we demonstrated that the abundance of PLK1 mRNA and protein increased in several breast cancer cell lines, compared to the normal‐like MCF10A (Fig. 2A). PLK1 pharmacological inhibition (volasertib) led to a reduction in uPA mRNA abundance in all the breast cancer cell lines and was apparently more potent than another protein kinase inhibitor identified in the screen (VEGFR2 inhibitor, regorafenib; (Fig. 2B). Several protein kinase inhibitors identified in the screen significantly reduced both uPA and IL‐8 mRNA abundance (except sorafenib) in MDA‐MB‐231 cell line (Fig. 2C).

### 2.17 The PLK1 inhibitor, volasertib, accelerates ARE‐mRNA decay

Volasertib reduced the levels of uPA and IL‐8 mRNAs in a dose‐dependent manner with an estimated ED_50_ in the vicinity of 100 nm, and the maximal effect was observed at 1 µm (Fig. 2D). Next, we evaluated the effect of volasertib on ARE‐mRNA decay and found that it significantly reduced the relative half‐life of uPA and IL‐8 mRNAs by at least sixfold (Fig. 2E). This suggests that PLK1 inhibition leads to accelerated ARE‐mRNA decay. Volasertib was indeed able to reduce the abundance of the ARE‐reporter mRNA, as measured by specific RT–PCR primers to the SuperNluc reporter mRNA (Fig. 2F).

### 2.18 ZFP36 phosphorylation in cancer cells and its reduction by PLK1 inhibition

**Fig. 3:**
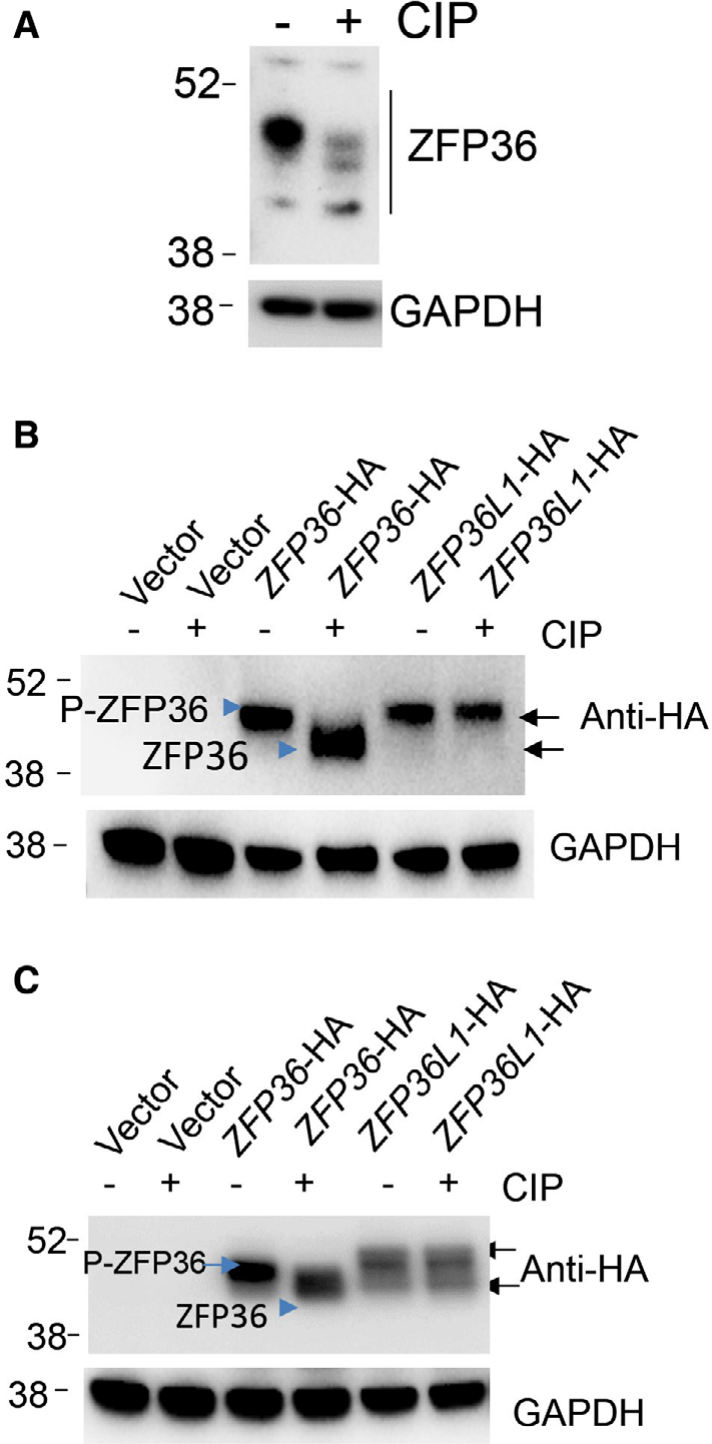
Endogenous and exogenous status of phosphorylated ZFP/TTP (A) Characterization of the protein bands in WB with anti‐ZFP36/TTP; lysates were treated with calf‐intestinal phosphatase (CIP) before separation on gels. Arrow indicates the phosphorylated form. (B, C) Determination of ZFP36/TTP and ZFP36L1 phosphorylation patterns in MDA‐MB‐231 and HEK293 cells; the cells were transfected either with control, ZFP36‐HA, or ZFP36L1‐HA vectors; lysates were treated with CIP. Western blotting was performed using anti‐HA as indicated. WBs are representative from two experiments. [Colour figure can be viewed at wileyonlinelibrary.com]

ZFP36/TTP is an ARE‐binding and mRNA decay‐promoting protein that is inhibited by phosphorylation [7]. Since PLK1 inhibitors are expected to inhibit phosphorylation events, we explored the effect of PLK1 pharmacological targeting on ZFP36/TTP phosphorylation. The size of TTP appears in multiple bands (40 to 50 kDa range) in PAGE blots due to phosphorylation modifications in different cell lines [29, 30, 31, 32, 33, 34, 35]. First, by using sensitive and specific western blotting for both unphosphorylated and phosphorylated ZFP36/TTP, we observed that in the triple‐negative MDA‐MB‐231 cell line, ZFP36/TTP largely existed as high‐molecular‐weight species as opposed to its 36‐kDa calculated weight and ~ 40 kDa apparent weight. This finding is consistent with literature indicating altered mobility of ZFP36/TTP protein is found during stimulated conditions, including tumorigenesis, because of phosphorylation [7, 35, 36]. We confirm that ZFP36/TTP bands reflected phosphorylated forms of TTP, since treatment with calf‐intestinal alkaline phosphatase demonstrated loss of upper bands (Fig. 3A). Previous reports indicate that phosphorylation events during inflammation lead to stabilization of both ZFP36/TTP mRNA and protein and that dephosphorylated TTP is unstable and much less abundant in cells [6, 7]. Ectopically overexpressed ZFP36/TTP became phosphorylated in MDA‐MB‐231 and HEK293 cells, since also treatment with CIP reduces its size (Fig. 3B). While the CIP treatment of cells transfected with ZFP36L1 (BRF1) did not lead to a similar effect (Fig. 3B,C), Both proteins are tagged with HA, allowing probing of both with antibody to HA.

**Fig. 4:**
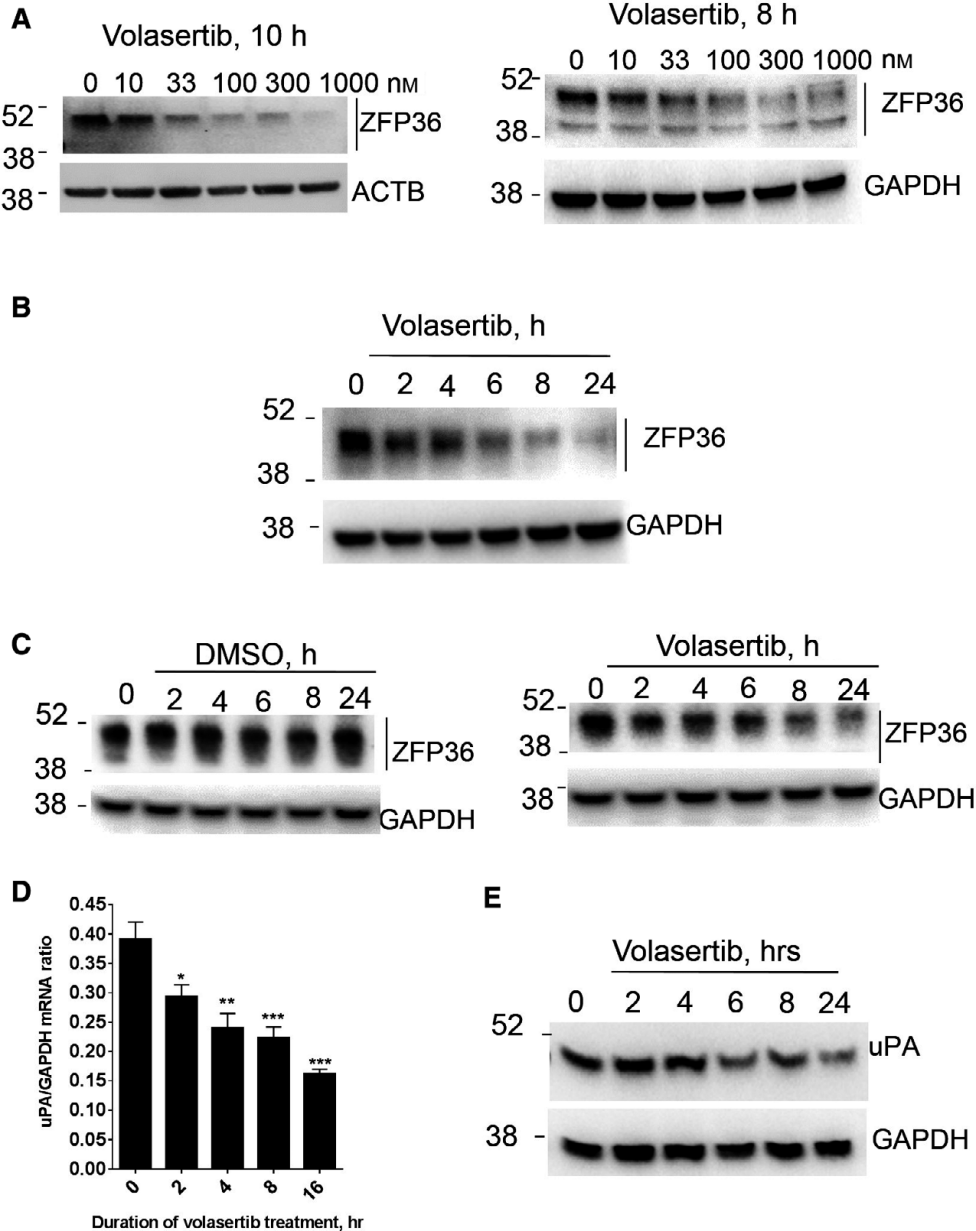
Response of TTP abundance to PLK1 inhibition. (A) Dose response for the PLK1 inhibitor (volasertib) effect on ZFP36/TTP level; WBs shown are from two independent experiments with 8 h and 10 h of treatments as indicated. (B, C) Effect of time course of volasertib (330 nm) action and DMSO control on ZFP36/TTP. WBs are representative of three independent experiments. (D, E) Time course of volasertib (330 nm) effect on uPA mRNA and protein abundance. Experiments are representative (with technical replicates of three in case of mRNA) of at least two independent experiments. [Colour figure can be viewed at wileyonlinelibrary.com]

Thus, we speculated that volasertib, as a kinase inhibitor, affects the status of ZFP36/TTP phosphorylation and its level. Indeed, compared to DMSO at 8 or 10 h, Volasertib reduced in a dose‐dependent manner the abundance of high‐molecular‐weight ZFP36/TTP likely due to reduced phosphorylation with the maximum dosage attained at 300 nm; the lowest tested dose of 10 nm was effective (Fig. 4A). There was no change in the mobility of ZFP36 as observed with CIP treatment, indicating a partial rather than complete dephosphorylation event (as with CIP). There was a time‐dependent reduction in ZFP36/TTP abundance, probably due to reduced phosphorylation (Fig. 4B,C). As early as 2 h and maximal at 8 h of inhibition were observed as a result of volasertib treatment (Fig. 4B,C). In contrast, DMSO at all durations of treatment did not reduce the abundance of TTP (Fig. 4C). The abundance of the uPA mRNA and protein was also reduced by volasertib (Fig. 4D,E).

**Fig. 5:**
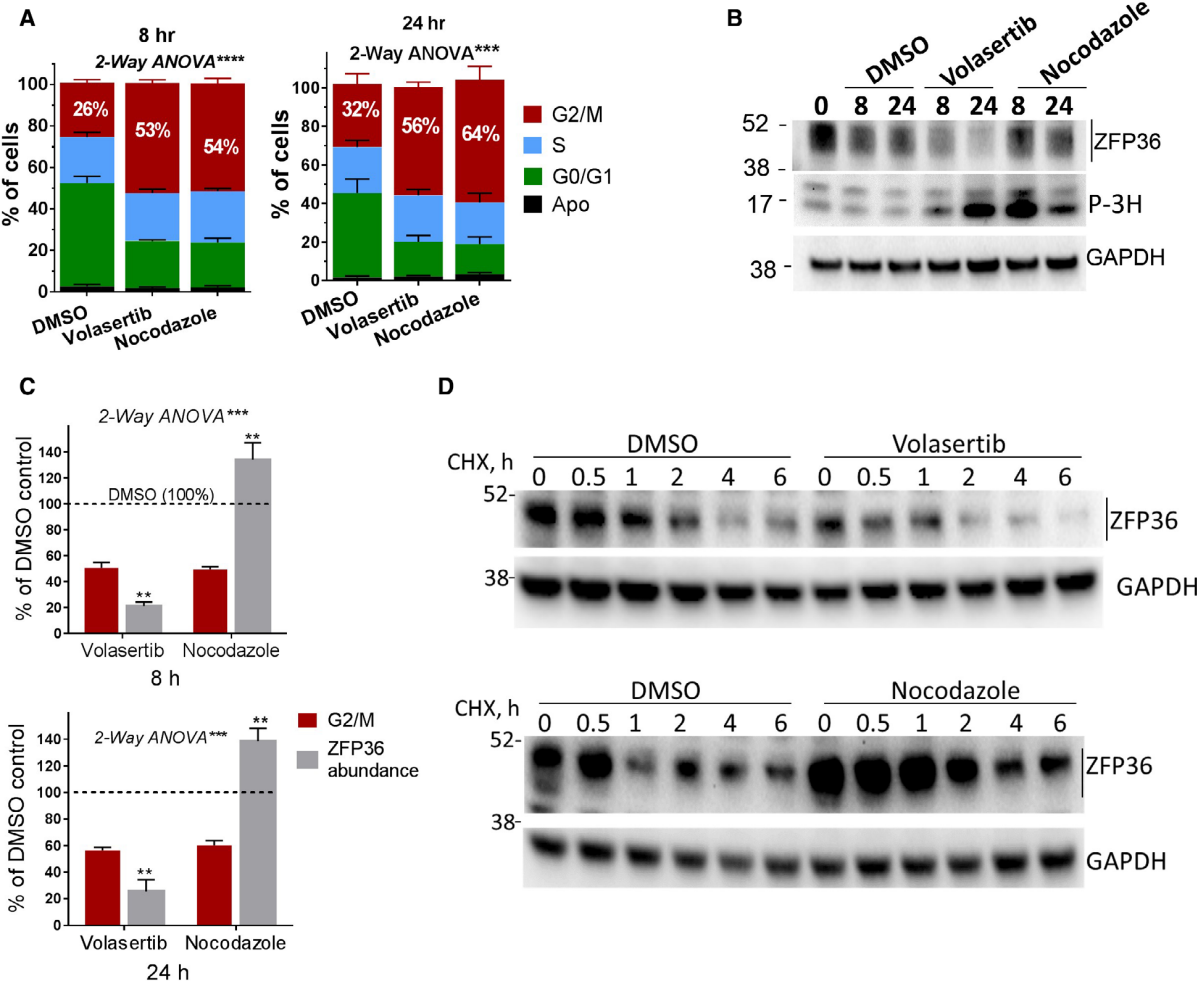
Comparative analysis of the effects of volasertib and nocodazole on cell cycle and ZFP36 abundance and stability. (A) Effect of volasertib and nocodazole on the cell cycle. MDA‐MB231 cells were treated with DMSO, volasertib (330 nm), or nocodazole (50 ng·mL^−1^) for 8 and 24 h. The proportion of the cells in the different phases of the cell cycle was assessed by flow cytometry. (B) The effect of volasertib and nocodazole on the levels of ZFP36 and histone 3 phosphorylation G2/M marker was assessed by western blotting. WB shown is a representative experiment of at least two. (C) Quantitative assessment of volasertib and nocodazole effects on G2/M arrest (flow cytometry) and abundance of P‐ZFP36/TTP (WB data). Data are mean ± SEM of independent experiments (*N* = 3–5) with readings of DMSO control are taken as 100%. **P < 0.01, ***P < 0.001 using two‐way ANOVA as indicated with Sidak's test used to compare two datasets within the larger dataset. (D) The stability of ZFP36/TTP protein. MDA‐MB‐231 cells were treated with DMSO, volasertib (330 nm), or nocodazole (50 ng·mL^−1^) for the indicated periods in the presence or absence of CHX. Data are a representative WB of two independent experiments. [Colour figure can be viewed at wileyonlinelibrary.com]

Both volasertib and nocodazole, a classical G2/M inhibitor, arrested cells in G2/M phase as assessed by flow cytometry and H3 phosphorylation (Fig. 5A,B). Figure 5A demonstrates that in the absence of the PLK1 inhibitor, the cell fraction at G2/M was only 26% at 8 h and, at most, 32% at the end of 24 h. The effects of volasertib and nocodazole at the experimental conditions used did not lead to the majority of the cells being at G2/M; only half of the cells were arrested (Fig. 5A). Most importantly, volasertib, unlike nocodazole, caused the specific reduction in the high‐molecular‐weight ZFP36/TTP (Fig. 5B). Figure 5C depicts the quantitative assessment of G2/M arrest and P‐ZFP36/TTP abundance as affected by volasertib and nocodazole treatment from multiple experiments. The reduction in ZFP36 abundance (>80%) was more substantial (*P* < 0.001) than the G2/M arrest (~50% average change) by volasertib with both 8 and 24 h of treatments. In contrast to volasertib, which always reduced ZFP36, nocodazole did not exert this reducing effect; in fact, in some of the experiments, it led to an opposite and slight increase (~40%) in ZFP36 /TTP abundance (Fig. 5C). Since ZFP36/TTP phosphorylation alters the protein's stability [6, 7], we investigated the effect of PLK1 inhibition on ZFP36/TTP protein stability. Cycloheximide (CHX) chase experiments were performed in the presence of vehicle, volasertib or nocodazole (Fig. 5D). The results indicate that volasertib is capable of reducing ZFP36/TTP protein stability most likely by reducing its phosphorylation. Nocodazole, on the other hand, did not reduce the stability of ZFP36/TTP protein; instead, it led to modest increase in protein stability (Fig. 5D, lower panel). Together, these results indicate that PLK1 inhibition (as compared to DMSO or the nocodazole) leads to specific reduction in phosphorylated ZFP36/TTP abundance and stability, particularly when compared to nocodazole, in a manner that may be distinct of G2/M arrest.

### 2.19 PLK1 increases the abundance and stability of ZFP36/TTP

**Fig. 6:**
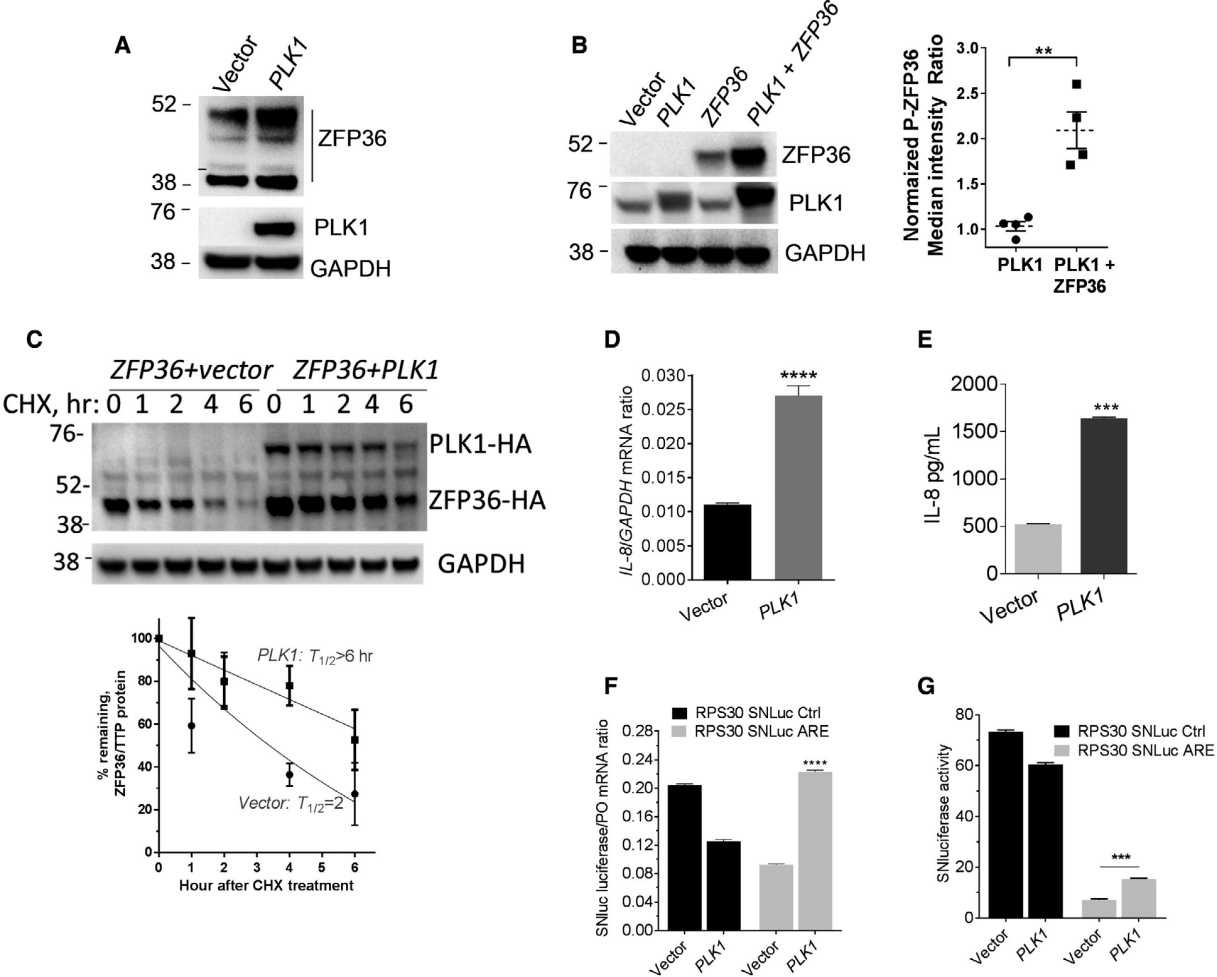
PLK1 increases the abundance and stability of ZFP36. (A) Ectopic expression of PLK1 in MCF10A; the cells were transfected with PLK1 vector (0.5 µg/million cells) for 24 h. The abundance of ZFP36/TTP was evaluated by WB (one shown from two independent experiments). (B) Cotransfection of HEK293 with PLK1 and ZFP36/TTP; the cells were cotransfected with PLK1 (0.2 µg/million cells) and ZFP36 (0.3 µg/million cells), and then, the abundance of ZFP36 was assessed by WB (one representative blot is shown from four independent experiments). Four blots were quantitated (*right panel*) using ImageJ software. Data are mean ± SEM of P‐ZFP36 median intensity ratio to GAPDH normalized to PLK1 vector alone as 1.0. ** denotes *P* < 0.005 using Student’s t‐test. (C) The effect of ectopic expression of PLK1 on the stability of ZFP36/TTP. HEK293 cells were cotransfected with either ZFP36/TTP and empty vector or ZFP36/TTP and PLK1 expression vectors. CHX chase was performed at the indicated time points, and the abundance of remaining ZFP36/TTP was evaluated by WB. Lower panel: Quantification of ZFP36/TTP abundance, normalized to GAPDH, was determined using ImageJ software and the estimation of the protein half‐life was assessed using the Graph Prism. Data are mean ± SEM of three experiments. (D, E) The effect of PLK1 overexpression in MCF10A cells on the endogenous IL‐8 mRNA and protein expression; cells transfected with vector or PLK1 vector (each 3 µg/million cells) for 24 h, and then, the IL‐8 mRNA and protein level were measured using RT–qPCR and ELISA, respectively. Data (mean ± SEM of replicates) from one experiment of two. (F) The effect of PLK1 overexpression in MCF10A cells on the Nluc ARE‐mRNA expression; the cells were transfected with PLK1 vector for 24 h, and then, the expression of ARE‐containing Nluc reporter was estimated by quantitative RT–qPCR. (G) Ectopic expression of PLK1 on the protein activity of Nluc bearing ARE in its 3`‐UTR. [Colour figure can be viewed at wileyonlinelibrary.com]

An independent line of evidence for the post‐transcriptional effects of PLK1 was sought by overexpressing PLK1 in MCF10A normal‐like cells, which express low PLK1 levels compared with tumor cells. PLK1 caused an increased abundance of the ZFP36/TTP (Fig. 6A). Moreover, using HEK293 cell line, which has nondetectable amounts of PLK1 and ZFP36 proteins, we showed that co‐expression of PLK1 and ZFP36/TTP led to increased abundance of the ZFP36 protein (2.1‐fold, *P* = 0.0023; Fig. 6B). All the bands in HEK293 are of higher molecular likely of phosphorylated forms, while lower unphosphorylated bands in HEK293 cells are usually not seen due to the protein instability [6, 7]. Cotransfection with SGFP did not affect the fluorescence levels due to PLK1, indicating the increase in the ZFP36 phosphorylation is not due to changes in transfection efficiency (data not shown). Cotransfection of ZFP36 with PLK1 expression vectors in HEK293 cells led to a marked increase in ZFP36 stability (Fig. 6C) from a protein half‐life of nearly two hours to more than six hours, according to estimations by cycloheximide chase experiments. These results indicate that PLK1 activity leads to stabilization of the ZFP36/TTP protein (Fig. 6C). PLK1 expression in MCF10A caused an increase in the abundance of IL‐8 mRNA (Fig. 6D) and also in secreted levels as measured by ELISA (Fig. 6E). Moreover, PLK1 caused an elevation in SNluc‐mRNA abundance that harbors the ARE sequence in its 3`‐UTR compared with non‐ARE control reporter mRNA (Fig. 6F). PLK1 increased the protein reporter activity; when normalized to the control, there was a 2.5‐fold increase (*P* < 0.0001) in the ARE‐reporter activity due to PLK1 expression (Fig. 6G).

### 2.20 Role of MK2 pathway in PLK1 effect on phosphorylation of ZFP36

**Fig. 7:**
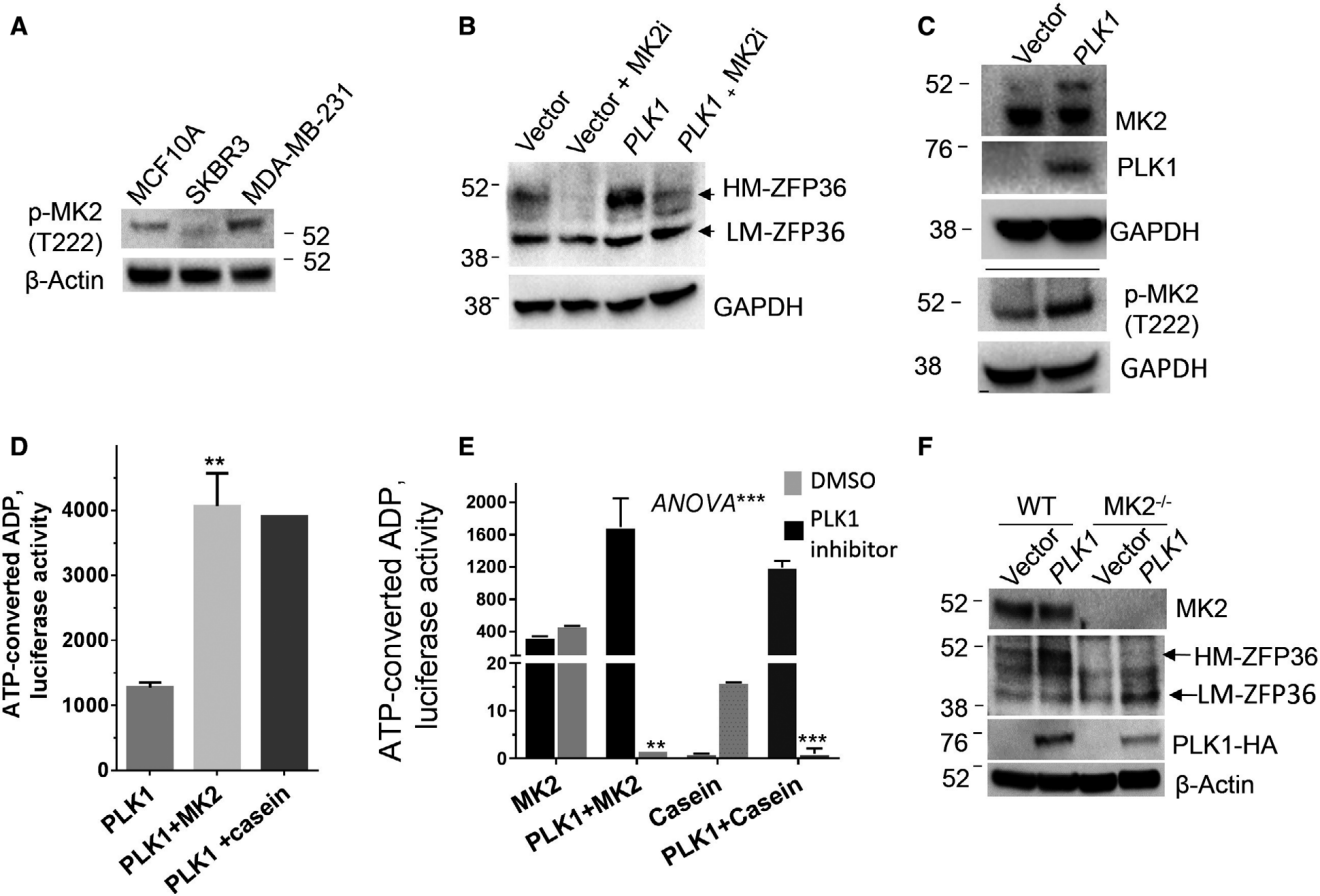
Role of MK2 pathway in PLK1‐induced TTP phosphorylation. (A) The abundance of phosphorylated MK2 in normal and cancer cells. Western blot using anti‐phospho‐MK2 and antibeta‐actin is a representative experiment of two independent experiments. (B) The effect of MK2 inhibition on ZFP36/TTP phosphorylation; MCF10 cells were transfected with either vector control or PLK1 expression vector, and then treated with 1 µm of the MK2 inhibitor, PF‐364402, for 4 h. WB shown is one of two blots from two independent experiments. (C) The effect of PLK1 overexpression on MK2 phosphorylation in MCF10A; the cells were transfected with PLK1 vector (0.5 µg/million cells) for 24 h. WB using antitotal and phosphor‐MK2 antibody is shown from one experiment—two blots. (D) PLK1 phosphorylation of MK2. The in vitro assay was performed with recombinant PLK1 (100 ng) and recombinant inactive (dephosphorylated) MK2 (100 ng) as a substrate or recombinant dephosphorylated casein as a positive control. ADP generated from ATP due to the kinase reaction was measured by chemiluminescence (ADP‐Glo assay). Data are from one experiment with duplicate readings. (E) In vitro PLK1 kinase inhibitor assay. The PLK1 inhibitor, volasertib, was used in the same kinase assay as above. Data were generated as described above. Data are relative luminescent units after subtraction of background from PLK1 autophosphorylation readings. One experiment with duplicate readings is shown, with 2‐way *ANOVA* and Sidak's post‐test to compare DMSO and PLK1 inhibitor. (F) WB analysis of total and phosphorylated ZFP36/TTP, and MK2, and PLK1 in MK2‐knockout MEF cells. The blot is representative of three independent experiments. [Colour figure can be viewed at wileyonlinelibrary.com]

The p38 mitogen‐activated protein kinase (MAPK)‐activated protein kinase‐2 (MK2) is a major regulator of mRNA stability by virtue of its ability of direct phosphorylation of ZFP36/TTP [37, 38, 39]. First, MDA‐MB231 was evaluated for its MK2 phosphorylation status (Fig. 7A), which has increased amounts of phosphorylated MK2 compared with the normal‐like MCF10A control. Due to the use of the MK2 inhibitor, PF‐364402, the PLK1‐induced increase in the high molecular ZFP36/TTP protein was significantly diminished in the normal‐like MCF10A (Fig. 7B). The ectopic expression of PLK1 in low‐PLK1 expressing MCF10A resulted in an increase in the abundance of phosphorylated MK2 (Fig. 7C). Moreover, using recombinant active PLK1 and recombinant inactive MK2 in kinase assay (*in vitro)*, we demonstrate that the recombinant PLK1 leads to MK2 phosphorylation (Fig. 7D). The PLK1 kinase substrate, casein, was used as a positive control (Fig. 7D). Further confirmatory experiments were performed in which the PLK1 inhibitor volasertib was added in the same in vitro kinase assay. The normalized background‐subtracted data demonstrate that the PLK1 inhibitor significantly reduced the extent of the MK2 phosphorylation, to a similar extent to the known PLK1 substrate casein, suggesting that it was largely due to PLK1 kinase action (Fig. 7E).

We tested the effect of MK2 knockout on PLK1‐induced ZFP36/TTP phosphorylation. Significant loss of the slow migrating ZFP36/TTP and further appearance of the smaller form of ZFP36/TTP was observed in MK2‐KO MEFs, suggesting reduced phosphorylation (Fig. 7F). Overexpression of PLK1 increased the abundance of the higher molecular weight phosphorylated form ZFP36/TTP only in WT cells but apparently had little effect on MK2‐KO cells, indicating an important requirement of MK2 in PLK1 effect on ZFP36/TTP phosphorylation. Together, these sets of experiments demonstrated a key role for MK2 in PLK1‐mediated phosphorylation of ZFP36/TTP.

### 2.21 Role of the major phosphorylation sites in PLK1 effect on ZFP36/TTP phosphorylation

**Fig. 8:**
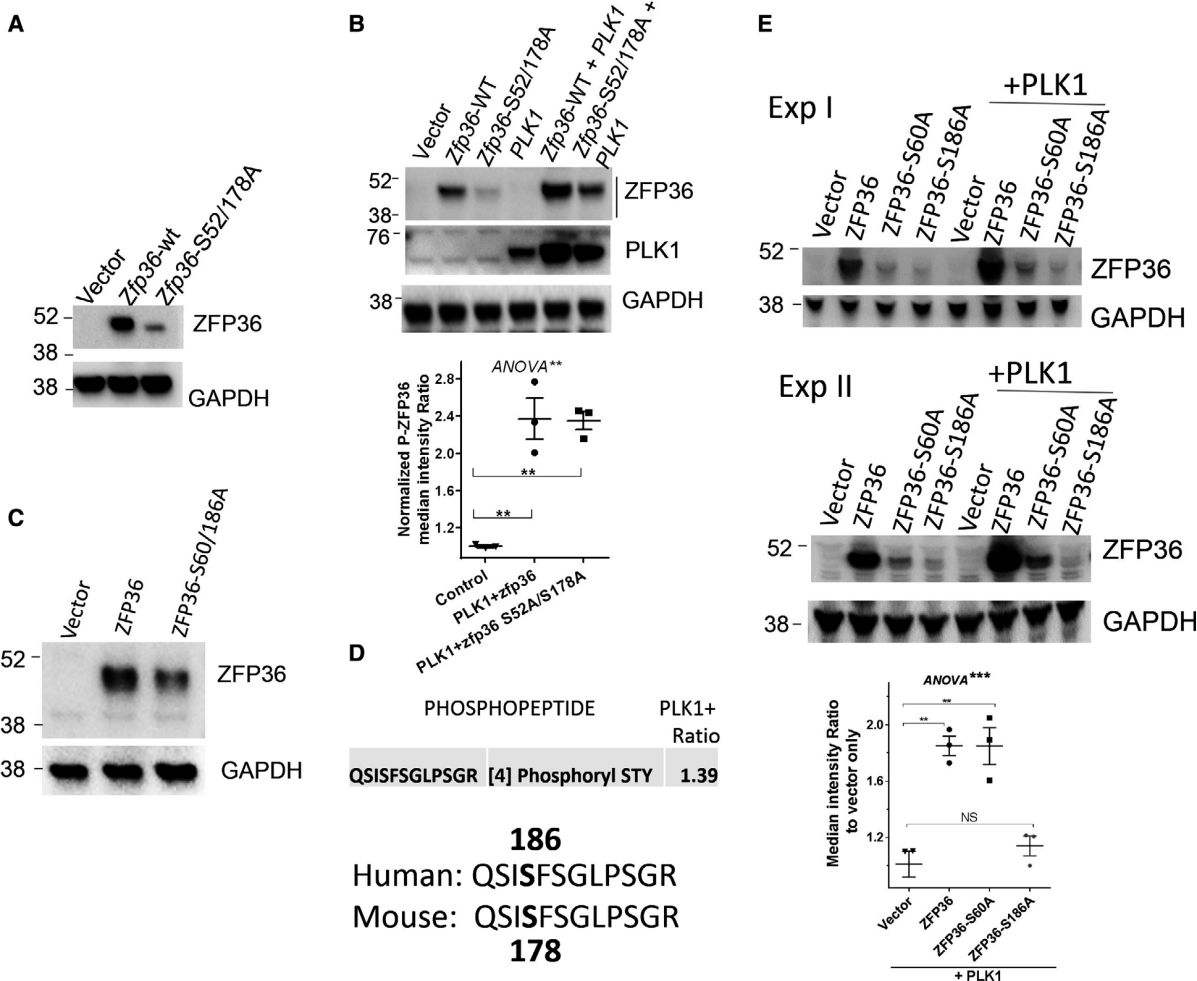
Role of MK2‐induced phosphorylation sites in PLK1 effects. (A) MDA‐MB‐231 cells were transfected with wild‐type ZFP36/TTP (ZFP36‐WT) or the double (serines 52/178)‐mutant mouse ZFP36/TTP. WB is one blot from at least two independent experiments. (B) The abundance of phosphorylated ZFP36/TTP in HEK293 after cotransfection with PLK1 and either ZFP36‐WT or ZFP36‐S52/178A in HEK293. WB is one of three blots from two independent experiments. Blot images were processed and analyzed by ImageJ software. Data are mean ± SEM of P‐ZFP36 median intensity ratio to GAPDH normalized to PLK1 vector alone as 1.0. (C) MDA‐MB‐231 cells were transfected with wild‐type human ZFP36/TTP (ZFP36‐WT) or the double‐mutant (serines 60/186) ZFP36/TTP. ANOVA with Sidak's post hoc tests was used to confirm the significance between two data as indicated (**P < 0.005). (D) HEK293 cotransfected with human ZFP36/TTP and either vector or PLK1 expression vector, and subjected to mass spectrometry analysis. The MS phosphopeptide mapping was performed using label‐free quantitative nanoacquity liquid chromatography coupled to Synapt G2 by ion mobility separation HDMSE, and data were analyzed by Progenesis QI for Proteomics (Waters, UK). Major phosphopeptide and phosphorylation sites are shown in bold at position 4. Ratio is the abundance of the ZFP36 phosphopeptide between PLK1 and vector samples. (E) The abundance of phosphorylated ZFP36/TTP in HEK293 after cotransfection with PLK1 and either ZFP36‐WT or ZFP36‐S60A or S186A in HEK293. Shown are blots from two independent experiments of three. Blot images (three) were processed and analyzed by ImageJ software. Data are mean ± SEM of ZFP36 median intensity ratio to GAPDH normalized to PLK1 vector alone as 1.0. Two‐way ANOVA with Tukey's post hoc tests was used to confirm the significance between two data as indicated (**P < 0.005, ***P < 0.0001). [Colour figure can be viewed at wileyonlinelibrary.com]

Two major MK2‐targeted phosphorylation sites were previously mapped, specifically serines 52 and 178 in the mouse ZFP36/TTP (corresponding to serines 60 and 186 in the human ZFP36/TTP [7, 37]. When the double‐mutant mouse S52/178A protein was expressed in MDA‐MB‐231, a significant reduction in the protein abundance was observed in contrast to the wild‐type ZFP36/TTP (Fig. 8A). Similar to MDA‐MB‐231, in HEK293 the double MK2 target mutant ZFP36 protein exhibited reduced phosphorylation when compared to the wild‐type (Fig 8B). Subsequently, we ectopically co‐expressed in HEK293, PLK1, with either wild‐type or MK2 target double‐mutant mouse ZFP36/TTP. PLK1 was still able to increase the abundance of both ZFP36 and the S52/178A‐mutant ZFP36 by nearly twofold ((*P* < 0.005) Fig. 8B), indicating that other MK2‐phosphorylatable serines/threonines may also be required in the mouse ZFP36/TTP.

Although most of the prior published studies were focused on the mouse serines 52 and 178 as major phosphorylation sites for MK2, in human ZFP36/TTP, the ortholog serine 186 but not serine 60 has been shown to be a major phosphorylation site [40]. Thus, we generated human ZFP36/TTP mutants (S60A and S186A). Like mouse ZFP36/TTP, there is a significant reduction in the abundance of the ZFP36/TTP (Fig. 8C). Unlike CIP treatment, which removes all phosphorylations and considerably reduces ZFP36/TTP to a lower band (Fig. 3A‐C), the human (S60/186A) mutant ZFP36/TTP band did not, at least appreciably, change its gel migration (Fig. 8C). Crude mass spectrophotometry analysis (i.e., without phosphopeptide enrichment) in HEK293 transfected with ZFP36/TTP showed one major abundant phosphopeptide harboring serine 186 as the major phosphorylation site which was also upregulated by PLK1 (Fig. 8D). Indeed, it appears that PLK1 was able to increase the abundance of wild‐type ZFP36 (1.85‐fold, *P* < 0.001) and S60A mutant ZFP36/TTP (fold, 1.84, *P* < 0.001) but minimal effect or no effect on S186A mutant ZFP36/TTP (Fig. 8E).

### 2.22 The effect of PLK1 inhibition on tumor xenografts in nude mice and ZFP36 phosphorylation

**Fig. 9:**
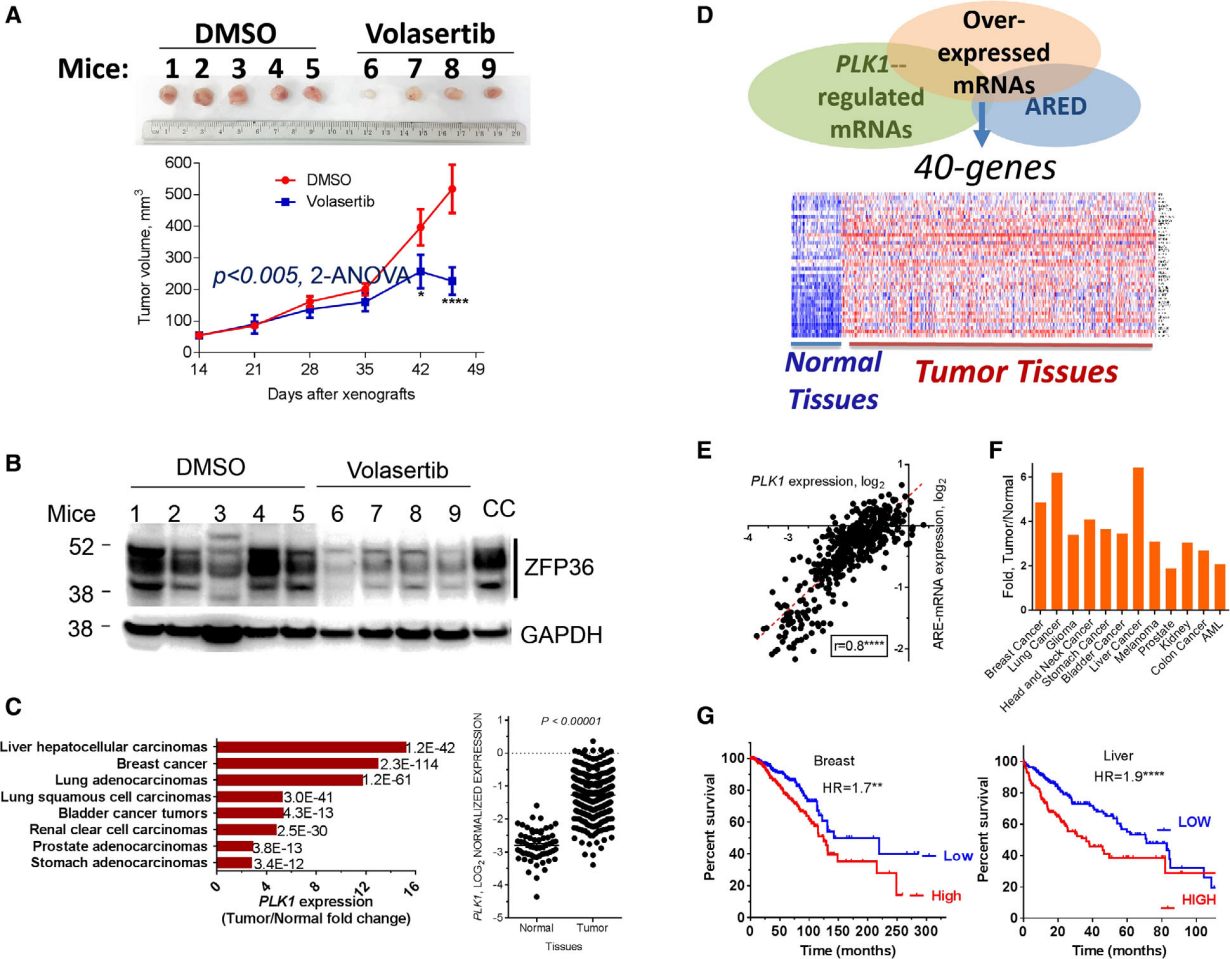
PLK1 inhibition in mice and PLK1/ARE‐mRNA expression patterns in cancer patients (A) PLK1 inhibition and tumor size in mice; MDA‐MB‐231 xenografts were injected into the mammary fat pad of female nude mice; when tumors become palpable, mice were treated with either volasertib (10 mg·kg^−1^) or vehicle twice weekly. The tumor size was calculated as described in Methods. Data are mean ± SEM from a nine‐mouse experiment as indicated. Two‐way ANOVA was performed for overall effect with Sidak′s multiple comparison test (*P < 0.05, ****P < 0.0001). (B) The effect of volasertib on ZFP36/TTP phosphorylation; WB of tumor tissues from each mouse as probed with anti‐ZFP36/TTP or GAPDH as the loading control. CC: lysates from MDA‐MB‐231 as WB‐positive control for phosphorylated ZFP36/TTP. (C) PLK1 expression in different types of cancer; RNA‐seq data of several cancer samples from TCGA patients were compared with normal tissue using Nextbio portal (left panel). Numbers indicate *P*‐values. Right panel, PLK1 overexpression in invasive ductal tumors relative to normal tissues. Data were obtained from TCGA (*P*‐values, Student′s *t*‐test). (D) *Upper panel*: Three data subsets were intersected to derive 40‐ARE‐mRNA group that are both overexpressed in breast cancer and PLK‐regulated (Table S5). The datasets are as follows: PLK1‐regulated mRNAs (*N* = 540 genes, RNA‐seq data; Fig. 1E), ARE mRNAs (ARE database, ARED), and overexpressed mRNAs in breast cancer (TCGA). *Lower pane:* heatmap for differentially expressed member of 40 ARE‐mRNA cluster in normal and patients' tumor sample‐TCGA invasive ductal breast cancer data. *Red indicates that the gene is overexpressed, blue indicates that the gene is underexpressed*. (E) Correlation plot between PLK1 mRNA expression (log2, x‐axis) and the average expression (log2, y‐axis) of the 40 ARE‐mRNA cluster members (above) using Pearson's correlation coefficient. (F) The average expression of the 40 ARE‐mRNA group in the tumor tissues was compared to the expression levels in the normal tissue samples. All patients' data were obtained from the public TCGA datasets. (G) The 40‐ARE‐mRNA expression and cancer patients′ survival. Kaplan–Meier relapse‐free survival (RFS) survival curves of breast and liver cancer patients with high versus low average expression of the 40 ARE‐mRNA group were generated. Data were generated using kmplot portal. [Colour figure can be viewed at wileyonlinelibrary.com]

To study the effect of PLK1 inhibition on TTP phosphorylation *in vivo* and the effect on tumor growth, MDA‐MB‐231 xenografts were injected into the mammary fat pad of female nude mice. Volasertib (10 mg·kg^−1^) or vehicle was administered biweekly upon the formation of palpable tumors. While the tumors in the control group continued to grow, those in the treatment group demonstrated a slower growth rate and began to regress by the end of the experiment (Fig. 9A, *upper panel*). A statistically significant difference in tumor volume could be seen after 4 weeks of treatment (Fig. 9A, *lower panel*). These results clearly demonstrate the role of PLK1 inhibition on tumor progression of MDA‐MB‐231 breast cancer cells. Next, we examined the *in vivo* effect of volasertib on TTP protein abundance in the excised tumor tissues. The amounts of the ZFP36/TTP levels in the mice tissues were markedly reduced due to the in vivo volasertib treatment most likely due to its reduced phosphorylation (Fig. 9B, lanes 6–9) compared with the control (lanes, 1–5), which clearly substantiates the *in vitro* data.

### 2.23 Correlation between PLK1 and ARE‐mRNA levels in cancer patients

To explore the inter‐relationships among PLK1, TTP, and ARE‐mRNA expression in cancer, we analyzed TCGA data for these genes. PLK1 was overexpressed in several types of cancers, including liver, breast, and lung (15‐, 13‐, and 12‐fold, respectively) compared with the abundance in normal tissue (Fig. 9C). PLK1 mRNA abundance is higher in breast invasive ductal patient tumor samples when compared to normal tissue samples (Fig. 9C, right panel). A 40‐ARE‐mRNA group that is both overexpressed in breast cancer and PLK1‐regulated was bioinformatically derived (Fig. 9D upper panel). Subsequently, this 40‐gene set (Table S5) was used to assess its differential gene expression between normal and breast cancer patients' tissues using TCGA data. Compared with the normal adjacent tissues, the 40 ARE‐mRNA group is significantly (*P* = 6.76E‐23, Fisher's exact test) upregulated (Odds ratio = 30, Fig. 9D lower panel). Moreover, this ARE‐mRNA cluster was indeed significantly correlated with PLK1 mRNA expression (Fig. 9E). Moreover, the 40‐gene group was significantly overexpressed in several solid tumors compared to their normal counterpart tissues (Fig. 9F). Remarkably, the 40‐gene cluster in breast cancer was associated with poor patient survival (Fig. 9G, left panel). This is not only observed in breast cancer but also in observed other solid cancers where PLK1 expression is high such as liver cancer (Fig. 9G, right panel). Among the 40‐gene group, the following members have been either validated as TTP ARE‐mRNA targets (*CDC6, CXCL8, E2F1, PLAU, and PLAUR)* [16, 22] or as ARE mRNAs that are correlated with TTP deficiency in breast cancer (*E2F8, CCNE2, IL11, LRRCC1, MCM10, RAD54L, RTKN2, and TMEM184A*) [22].

#### 2.23.1 Discussion

Protein phosphorylation by kinases is a post‐translational mechanism that affects numerous cellular responses to stimuli and influences downstream transcriptional and post‐transcriptional events. Human cells contain hundreds of kinases, many of which can be aberrantly active in cancer cells [10, 41]. Such kinase activity can cause abnormal regulation of gene expression at different levels. Today, post‐transcriptional control is more intricate and complex than previously thought, and as crucial as epigenetic and transcriptional regulation. Specifically, the ARE‐mediated post‐transcriptional defects exist almost in all hallmarks of cancer [1], and AREs are present in so many mRNAs (estimated to be 20% of human genes: [28]. Therefore, to pinpoint abnormally active kinases in cancer cells that affect ARE‐mediated post‐transcriptional control, we evaluated 378 kinase inhibitor drugs using a cell‐based reporter system. As a result, we discovered that PLK1 is a key factor in the ARE‐mediated post‐transcriptional control in cancer cells and exerts its effect through MK2‐mediated phosphorylation of ZFP36/TTP. This activity is demonstrated in vitro, as evidenced by the use of a pharmacological PLK1 inhibitor.

PLKs regulate cell cycle and are activated by upstream kinases through phosphorylation of PLK catalytic kinase domain [42]. PLK1 is expressed in the late interphase (G2) and M phases. Entry into mitosis is controlled by PLK1, a process that is attributed to Cdk1–cyclin‐B activation [43]. Moreover, PLK1 is tightly regulated at the expression level, being low at interphase and high during mitosis. In cancer cells, PLK1 aberrantly localizes in the nucleus early in the interphase, after being involved in the G1–S transition and DNA replication [44]. The involvement of PLK1 in advancing mitotic events is in agreement here with the bioinformatic data of increased abundance of PLK1 and its regulated ARE‐coding genes (which include cell cycle genes) across different cancers. Our data demonstrate the effect of PLK1 inhibitor on the ARE‐mediated pathways, for example, ZFP36/TTP phosphorylation, is both ARE‐dependent (compared to non‐ARE control) and specific (compared to DMSO). Moreover, unlike the PLK1 inhibitor, the well‐established G2/M inhibitor, nocodazole, caused G2/M arrest without reducing ZFP36 abundance.

The kinome analysis showed that the PLK1 inhibitor, volasertib, was the most consistent among other inhibitors in reducing the post‐transcriptional reporter activity. Another PLK1 inhibitor, BI2536, is active in this kinome screen; however, other PLK1 inhibitors did not show this activity. This may be due to the type of the cancer cells, drug sensitivity, or limitation of the assay conditions (e.g., duration and dose). RNA‐seq analysis demonstrated that the genes involved in cell cycle and DNA replication are downregulated by PLK1 inhibition (volasertib) in line with PLK1 cell cycle effects. Other PLK1‐regulated genes found here are also involved in other hallmarks of cancer. Indeed, it has been shown that PLK1 mediates tamoxifen resistance in breast cancer [45]. Additionally, a set of genes that belong to inflammatory and cytokine response was found here to be PLK1‐regulated (including post‐transcriptionally in case of genes such as IL‐8 and uPA). The data may stimulate further evaluation of PLK1 inhibitors as treatment for chronic inflammatory conditions characterized by overexpression of pro‐inflammatory cytokines coded by ARE‐mRNA, such as TNF.

Beyond the cell cycle regulation, there is increasing evidence that PLKs play regulatory roles in different cellular pathways by phosphorylation of an increasing number of newly discovered substrates [46]. In our data, PLK1 leads to ZFP36/TTP phosphorylation, and this may be due to MK2 phosphorylation—at least a good proportion—as confirmed by different lines of evidence. It is known that ZFP36/TTP harbors multiple phosphorylation sites [31], and thus can be affected by several signaling pathways and many kinases [47]. The p38/MK2 is a well‐established pathway that leads to TTP phosphorylation preventing its ability to recruit mRNA decay machinery and subsequently leading to overproduction of ARE‐mRNA products [18, 38]. Unlike the active unphosphorylated ZFP36/TTP, MK2‐phosphorylated ZFP36/TTP is of increased abundance due to protein stabilization, but, more importantly, of lesser activity [6, 7, 48]. In our study, PLK1 modulation led to ZFP36/TTP protein stability alterations in concurrence with the role of MK2 pathway in the stabilization of ZFP/36 protein.

Moreover, unlike the PLK1 inhibitors, merely blocking the cells in G2/M—by nocodazole—does not reduce the high‐molecular‐weight ZFP36/TTP abundance. Therefore, it is expected that this likely does not decrease the consequent protein stability according to the feature that phosphorylation of ZFP36/TTP affects its protein stability. There is even a modest increase in the protein abundance and stability due to nocodazole. Our results showed that PLK1 can lead to MK2 phosphorylation both in the cells and *in vitro* and that TTP phosphorylation is largely lost during MK2 pharmacological inhibition or genetic ablation. Thus, we propose that MK2 is a target for PLK1‐induced phosphorylation leading to ZFP36/TTP phosphorylation regulating the protein stability of TTP. A previous report showed the reverse, that is, PLK1 (at serine 326) is a direct target for MK2 phosphorylation [49]; this at least demonstrates the physical interaction between PLK1 and MK2 and the possible bidirectional phosphorylation.

A role was proposed for MK2 in cell cycle progression, for example, in G2/M arrest during DNA damage [50], and in tumorigenesis of several cancers (Reviewed in: [51]. Our results ascertain a significant role of MK2‐TTP phosphorylation activity in cancer [8], since most of the studies in this respect were in the context of inflammation. An added novelty in our study is that PLK1–MK2–TTP phosphorylation axis is operative in cancer. It is also possible that this axis is functional in inflammatory response since IL‐8, and potentially other pro‐inflammatory cytokines/chemokines are, as well, demonstrated here to be regulated. This can make PLK1–MK2–TTP an attractive pharmacological target for both cancer and chronic inflammatory conditions—the latter is important for the tumor microenvironment.

The major MK2 sites for ZFP36/TTP phosphorylation are mouse/human serine 52/60 and 178/186 [37, 52]. The expression of the double‐mutant (S52/178A or S60/186A) mouse and human protein resulted in a significant reduction in the phosphorylated protein in the invasive breast cancer cell line and also in HEK293 cells. These results indicate the importance of these two serine sites in ZFP36 phosphorylation in these cells, which renders ZFP36/TTP highly stable yet inactive [6, 7, 53], whereas unphosphorylated TTP is highly unstable and thus can hardly be detected in western blots. PLK1 overexpression is capable of upregulating the expression of both wild‐type mouse and human ZFP36/TTP but with different outcomes with respect to the known MK2‐induced phosphorylation sites. It is possible that the activities of PLK1 and MK2 kinases lead to phosphorylation of the same sites, during inflammatory response [6] or cell cycle changes. In the case of the mouse ZFP36/TTP, additional alternative amino acid sites for PLK1 effect are probable. However, in human ZFP36/TTP, serine 186 was the predominant phosphorylation site, and the PLK1 effect appears to be dependent on serine 186. In a phosphopeptide mass study, the serine 186 appeared as a major phosphorylation site during cell cycle changes [54]. Our data agree with a previous report [30] that serine 186 mutation does not change ZFP36/TTP gel mobility. This also agrees to the fact the PLK1 forced expression or the PLK1 inhibitor, volasertib, did not change ZFP36/TTP mobility. Clearly changed mobility of ZFP36/TTP was seen with treatment by CIP, which removes all phosphorylation groups in the protein.

The experiments with mice treated with the PLK1 inhibitor, volasertib, reduced tumor growth and demonstrated the remarkable decrease in the abundance of the phosphorylated ZFP36/TTP as observed with the cell line models. However, direct demonstration of TTP phosphorylation as a mechanism of PLK1 inhibition of tumor growth cannot be inferred from these mouse studies and requires a different set of experiments. In patients, the bioinformatic analysis demonstrates that the PLK1‐regulated ARE‐mRNA group is overexpressed in different cancer types and correlated with PLK1; besides, the gene group correlated with patients' poor survival as seen at least in breast cancer. Many of these mRNAs are likely targets for ZFP36/TTP since several of them, namely CCNE2, CDC6, CXCL8, E2F1, PLAU, and PLAUR, are established ZFP36 mRNA targets [16, 22, 55, 56].

## 3 Conclusions

Our results indicate that the increased abundance and activity of PLK1 in cancer may not be restricted to dysfunction in the classical role of PLK1 in the cell cycle, but can also lead to an upregulation in the expression of ARE‐coding genes, including pro‐inflammatory, chemotactic, and metastatic players. PLK1 seems to be a promising target for cancer therapy; its inhibition could repress multiple aberrations by a single agent. Together, our data strongly support a role for PLK1 in ZFP36/TTP‐regulated post‐transcriptional control that is compromised in cancer, and targeting this pathway can restore the aberrant activities. Moreover, the screen data offer other kinases that can be further explored for targeting post‐transcriptional defects in cancer.

## Conflict of interest

The authors declare no conflicts of interest.

## Author contributions

H.A. Qamraa, W.N. Moghrabi, S. Al‐Yahya, L. Al‐Haj, L. Mahmoud, M. Al‐Saif, F. Al‐Mohanna performed experiments. F. Al‐Mohanna and N. Al‐Souhibani designed and analyzed the animal studies. A. Alaiya performed MS work and analysis. Q.H. Al‐Qahtani, N. Al‐Souhibani, E. Hitti, and K.S.A. Khabar designed the experiments and analyzed the data. Q.H. Al‐Qahtani and K.S.A. Khabar wrote the manuscript. E. Hitti and K.S. A. Khabar revised the manuscript.

## Supporting information


**Table S1.** Data of primary kinase inhibitors screen of the post‐transcriptional ARE‐reporter activity.
**Table S2.** Data of secondary kinase inhibitors screen of on the post‐transcriptional ARE‐reporter activity.
**Table S3.** Down‐regulated expressed genes (at least 1.7 fold, *P* < 0.001) as a result of the PLK1 inhibitor, volasertib, treatment.
**Table S4.** The list of genes and there are sequences due to crossing of ARED with the PLK1 inhibitor reduced genes. Table S5 list of the 40‐gene cluster and their ARE sequences.
