# Supplementary material for: Kinome inhibition reveals a role for polo‐like kinase 1 in targeting post‐transcriptional control in cancer
Source: Mol Oncol. 2021 Feb 1;15(8):2120–39. doi: 10.1002/1878-0261.12897 (PMC8334256; doi:10.1002/1878-0261.12897)
Supplement: Supplementary file 1 — Table S1. Data of primary kinase inhibitors screen of the post‐transcriptional ARE‐reporter activity. Table S2. Data of secondary kinase inhibitors screen of on the post‐transcriptional ARE‐reporter activity. Table S3. Down‐regulated expressed genes (at least 1.7 fold, P < 0.001) as a result of the PLK1 inhibitor, volasertib, treatment. Table S4. The list of genes and there are sequences due to crossing of ARED with the PLK1 inhibitor reduced genes. Table S5 list of the 40‐gene cluster and their ARE sequences. [file MOL2-15-2120-s001.pdf]

Supplemental Table S1 Primary screen

**DRUG**	Linifanib	Cediranib	Lapatinib	Sorafenib	AC480	SB203580	KU-55933	LY294002	Palbociclib	Brivanib	CP-724714	DMSO
**MEAN**	19627	4518	19032	7178	13736	9381	9448	7754	7072	8448	19077	11677
**SEM**	668	1355	2576	1439	1096	146	814	704	348	207	1051	113
**SSDM**	-7.37	3.12	-2.18	1.57	-1.48	9.36	1.97	3.90	8.40	8.86	-4.38	0.00
**Fold Change**	1.43	0.49	1.50	0.55	1.15	0.83	0.84	0.80	0.74	0.77	1.64	1.00

**DRUG**	Axitinib	Dovitinib	Motesanib	Sunitinib	Masitinib	SB202190	GSK1904529 A	OSU-03012	Triciribine	AG-490	TGX-221	DMSO
**MEAN**	9950	6634	0	4081	19231	9727	10180	2126	9210	9375	6660	8909
**SEM**	154	522	2	152	897	400	917	904	1093	759	431	219
**SSDM**	-3.33	2.66	-4.83	20.17	-7.10	-1.01	-0.14	5.18	-0.33	-0.07	2.91	0.00
**Fold Change**	1.26	0.83	1.40	0.73	2.04	1.33	1.16	0.90	1.10	1.04	0.95	1.00

**DRUG**	Saracatinib	PD184352	Nilotinib	Tandutinib	GDC-0941	MK-2206	PF-04217903	Danusertib	XL147	SNS-032	WZ3146	DMSO
**MEAN**	8525	8384	6050	8571	6626	6400	10433	1927	11188	3748	1724	9213
**SEM**	299	838	622	1038	384	494	454	345	1222	640	606	61
**SSDM**	**1.53**	**1.24**	**5.53**	**13.85**	**27.69**	**1.94**	**3.06**	**5.92**	**0.53**	**3.61**	**2.05**	**0.00**
**Fold Change**	1.03	0.89	0.72	0.89	0.83	0.82	1.09	0.24	1.22	0.49	0.93	1.00

**DRUG**	Selumetinib	Dasatinib	NVP-AEW541	Temsirolimus	SL-327	SU11274	MLN8054	BI 2536	Cabozantinib	Barasertib	CYC116	DMSO
**MEAN**	8027	8123	2024	9709	8690	9589	10998	5047	5045	12551	7389	7447
**SEM**	603	538	288	1014	606	529	125	732	552	1391	1394	371
**SSDM**	**-1.03**	**-1.15**	**10.67**	**-0.82**	**-1.18**	**-2.67**	**-18.21**	**1.78**	**3.10**	**-1.71**	**0.05**	**0.00**
**Fold Change**	0.99	1.01	0.87	1.12	1.02	1.22	1.28	0.65	0.75	1.48	0.92	1.00

**DRUG**	Nintedanib	Ridaforolimus	Pazopanib	Vandetanib	Crizotinib	Brivanib	Vatalanib	Foretinib	Everolimus	PLX-4720	WZ4002	DMSO
**MEAN**	7054	7739	5884	4803	1323	8117	6685	2375	8958	7457	3585	9315
**SEM**	670	595	375	198	170	358	605	685	1401	335	1858	428
**SSDM**	**1.53**	**1.24**	**5.53**	**13.85**	**27.69**	**1.94**	**3.06**	**5.92**	**0.53**	**3.61**	**2.05**	**0.00**
**Fold Change**	0.90	0.89	0.83	0.67	0.76	0.97	0.89	0.90	1.00	0.87	0.86	1.00

**DRUG**	Afatinib	Erlotinib	PD0325901	VX-680	PHA-665752	NVP-ADW742	U0126-EtOH	SGX-523	BMS-754807	Roscovitine	PD98059	DMSO
**MEAN**	8283	10825	7185	7827	6275	2001	19514	9949	1979	7379	6655	7742
**SEM**	1325	184	418	243	557	634	7460	185	131	535	323	434
**SSDM**	**-0.39**	**-10.03**	**0.56**	**0.51**	**1.48**	**5.62**	**-0.37**	**-6.51**	**28.05**	**0.53**	**2.52**	**0.00**
**Fold Change**	1.03	1.26	0.78	1.04	0.99	0.84	1.21	1.12	0.50	0.94	0.88	1.00

DRUG	BMS-536924	Gefitinib	PI-103	Y-27632 2HCl	ZSTK474	Refametinib	ZM 447439	GSK690693	Alisertib	SNS-314 Mesylate	Regorafenib	DMSO
**MEAN**	3513	11941	4600	5921	4156	6169	12851	21323	10865	13380	5298	10112
**SEM**	263	341	928	31	139	149	733	2955	231	1287	377	838
**SSDM**	**13.55**	**-4.34**	**3.04**	**67.51**	**22.10**	**12.42**	**-3.42**	**-2.47**	**-3.51**	**-1.46**	**6.83**	**0.00**
**Fold Change**	0.45	1.07	0.60	0.73	0.56	0.63	1.26	2.11	0.97	1.28	0.65	1.00

DRUG	Bosutinib	Imatinib	Sirolimus	Enzastaurin	SB216763	OSI-906	GDC-0879	JNJ-38877605	AT9283	Lenvatinib	WZ8040	DMSO
**MEAN**	8988	9839	10001	10530	10411	4793	7907	10259	1360	6733	3275	8576
**SEM**	785	1108	1202	595	204	241	928	1020	123	147	490	583
**SSDM**	**-0.67**	**-0.68**	**-0.78**	**-2.49**	**-6.99**	**8.72**	**0.17**	**-1.20**	**32.63**	**4.83**	**6.27**	**0.00**
**Fold Change**	1.03	1.21	1.06	1.07	1.13	0.59	0.86	1.16	0.23	0.87	0.53	1.00

DRUG	ENMD-2076	Amuvatinib	TG100-115	PHA-680632	HMN-214	AT7519	AZD8055	Fasudil	AZD8330	GSK461364	Mubritinib	DMSO
**MEAN**	966	1915	2500	1548	2171	1136	1383	1866	1531	1889	2099	1891
**SEM**	95	103	534	40	162	63	104	120	155	70	153	139
**SSDM**	**9.30**	**-0.13**	**0.10**	**8.70**	**-0.95**	**10.11**	**4.41**	**0.22**	**2.71**	**0.79**	**-0.61**	**0.00**
**Fold Change**	0.53	1.04	1.06	0.84	1.17	0.61	0.74	1.00	0.82	1.04	1.13	1.00

DRUG	CUDC-101	JNJ-7706621	GSK1059615	VX-745	AEE788	Quizartinib	PHT-427	BIRB 796	KW-2449	R406	PP242	DMSO
**MEAN**	4444	3162	1569	1945	1904	1733	1778	1634	1289	1497	1024	1908
**SEM**	355	234	111	223	163	183	124	191	101	99	57	97
**SSMD**	**-6.21**	**-4.18**	**2.96**	**-0.28**	**0.18**	**1.32**	**1.21**	**1.90**	**4.91**	**3.58**	**13.63**	**0.00**
**Fold Change**	2.35	1.67	0.83	1.01	1.01	0.90	0.94	0.85	0.68	0.80	0.54	1.00

DRUG	PIK-75	PD173074	MGCD-265	Thiazovivin	PHA-793887	Hesperadin	KRN 633	Tie2 kinase inhibitor	RAF265	SGI-1776	CYT387	DMSO
**MEAN**	882	1494	1751	1588	1413	930	2137	2041	1387	1220	1647	1909
**SEM**	104	163	93	166	161	26	87	101	91	100	80	34
**SSMD**	**9.67**	**2.31**	**1.94**	**2.09**	**3.33**	**32.28**	**-2.65**	**-1.35**	**4.58**	**6.55**	**2.52**	**0.01**
**Fold Change**	0.46	0.79	0.92	0.83	0.74	0.49	1.12	1.07	0.73	0.64	0.86	1.00

DRUG	Tivozanib	WYE-354	Rigosertib	SP600125	PIK-93	BIX 02188	AT7867	H 89 2HCl	AZD1480	BMS-794833	SB590885	DMSO
**MEAN**	2133.33	2106.33	2410.17	2243.67	1540.33	1558.83	1141.83	1810.50	1296.83	1578.67	1386.00	1930.00
**SEM**	223.00	256.80	112.59	187.95	112.96	112.64	58.31	155.56	64.85	148.53	83.65	247.26
**SSMD**	**-0.62**	**-0.42**	**-4.85**	**-2.07**	**1.00**	**0.76**	**8.17**	**0.09**	**5.60**	**1.86**	**3.64**	**0.00**
**Fold Change**	1.16	1.19	1.35	1.19	0.84	0.84	0.64	1.01	0.70	0.85	0.76	1.00

DRUG	YM201636	Vemurafenib	Ki8751	AZD6482	Ponatinib	BIX 02189	BMS-777607	TWS119	PF-4708671	NVP-BHG712	Apatinib	DMSO
**MEAN**	1471	1833	1797	1508	1025	1767	2165	2012	1928	2839	2540	2261
**SEM**	298	112	54	119	77	105	128	124	118	235	328	472
**SSMD**	**0.30**	**0.33**	**0.58**	**2.41**	**8.30**	**0.07**	**-2.37**	**-1.52**	**-1.45**	**-4.33**	**-1.21**	**0.00**
**Fold Change**	0.81	0.91	0.91	0.89	0.52	0.87	1.07	1.00	0.96	1.50	1.31	1.00

DRUG	OSI-930	BX-795	Ruxolitinib	Orantinib	LY2228820	AZD7762	PD318088	Acadesine	LY2784544	OSI-420	CAL-101	DMSO
**MEAN**	1861	1890	2251	1693	1721	855	1334	1850	2266	2044	1587	1618
**SEM**	111	195	201	60	148	48	40	118	160	205	145	74
**SSMD**	**-2.07**	**-0.67**	**-2.61**	**-0.81**	**-0.01**	**13.41**	**4.99**	**-2.33**	**-3.33**	**-1.44**	**0.47**	**0.01**
**Fold Change**	1.17	1.16	1.42	1.06	1.08	0.53	0.84	1.16	1.42	1.27	0.99	1.00

DRUG	KU-0063794	BX-912	Pelitinib	GSK429286A	CCT129202	R406	KU-60019	PF-573228	AST-1306	PIK-293	PIK-294	DMSO
**MEAN**	1153	1511	1596	1536	1715	1903	1776	1883	2758	2170	1997	2065
**SEM**	103	106	54	108	94	179	135	322	299	119	137	95
**SSMD**	**7.89**	**4.04**	**6.31**	**2.88**	**3.47**	**1.60**	**0.56**	**0.86**	**-1.85**	**-1.38**	**0.34**	**0.00**
**Fold Change**	0.57	0.74	0.78	0.75	0.85	0.94	0.88	0.95	1.35	1.06	0.98	1.00

DRUG	AG-1024	Genistein	Aurora A Inhibitor I	Pimasertib	SAR245409	CP-673451	BS-181 HCl	BMS-265246	Sapitinib	AZ 960	Telatinib	DMSO
**MEAN**	1905	2188	1919	1806	2063	1673	1846	1801	1941	1870	1735	1576
**SEM**	140	264	230	449	126	175	77	124	228	224	93	76
**SSMD**	**-2.13**	**-1.41**	**-1.08**	**0.37**	**-2.04**	**0.62**	**-1.23**	**-0.81**	**-0.15**	**0.11**	**-0.40**	**0.96**
**Fold Change**	1.22	1.39	1.21	1.13	1.32	1.07	1.18	1.15	1.23	1.21	1.11	1.00

DRUG	Volasertib (BI 6727)	Chrysophanic Acid	LY2603618	AS-252424	NVP-BSK805	GSK1838705A	Crenolanib	BGT226	Dinaciclib	WP1066	Torin 2	DMSO
**MEAN**	3690	5247	5801	5472	4688	4422	4788	1473	4455	6477	3416	6287
**SEM**	292	226	267	239	167	150	330	260	188	420	84	216
**SSMD**	**7.34**	**3.61**	**2.05**	**3.27**	**8.73**	**11.42**	**3.98**	**16.64**	**8.24**	**-0.62**	**29.31**	**0.00**
**Fold Change**	0.59	0.84	0.93	0.87	0.75	0.70	0.76	0.23	0.72	1.03	0.55	1.00

DRUG	Palomid 529	Imatinib	DCC-2036	PF-00562271	R547	TAK-901	MK-8776	Milciclib	Dovitinib	AZD4547	TAE226	DMSO
**MEAN**	9438	6553	3745	4725	5039	1420	5747	5394	4422	6482	2958	5276
**SEM**	588	287	239	365	341	71	222	288	259	309	74	194
**SSMD**	**-6.12**	**-3.64**	**6.76**	**1.86**	**0.67**	**47.21**	**-2.24**	**0.37**	**3.38**	**-2.80**	**26.89**	**0.00**
**Fold Change**	1.79	1.25	0.71	0.89	0.95	0.27	1.10	1.02	0.84	1.23	0.56	1.00

DRUG	Degrasyn	Phenformin	CCT128930	Trametinib	WAY-600	AMG-900	TG101348	HER2-Inhibitor-1	MK-5108	CEP-33779	TPCA-1	DMSO
**MEAN**	3438	5380	3599	3886	5234	6209	2974	6398	7752	5692	5986	6069
**SEM**	483	289	219	157	407	378	203	210	279	340	219	310
**SSMD**	**4.01**	**1.71**	**8.61**	**10.53**	**1.31**	**-0.79**	**12.34**	**-2.46**	**-6.07**	**0.89**	**-0.98**	**0.00**
**Fold Change**	0.56	0.90	0.60	0.65	0.87	1.03	0.49	1.07	1.29	0.94	0.99	1.00

DRUG	BKM120	TAK-733	A66	Flavopiridol	TG101209	ZM 336372	PHA-767491	Varlitinib	MK-2461	Dabrafenib	SAR131675	DMSO
**MEAN**	4616	4427	5147	3166	3340	6523	5808	8413	6305	6969	5531	5467
**SEM**	153	185	138	176	150	304	351	254	233	485	337	131
**SSMD**	4.52	5.06	2.82	11.30	12.40	-3.06	-0.58	-10.26	-2.86	-3.27	0.46	0.00
**Fold Change**	0.85	0.81	0.94	0.58	0.61	1.20	1.06	1.54	1.15	1.28	1.01	1.00

DRUG	Asiatic Acid	AZD5438	NU7441	Ibrutinib	GDC-0980	PH-797804	PF-04691502	Wortmannin	AZD2014	GDC-0068	BI-D1870	DMSO
**MEAN**	5397	5439	4264	4292	3156	4816	3588	4772	4445	4561	6702	6337
**SEM**	274	499	196	201	112	238	343	178	196	104	148	196
**SSMD**	**2.43**	**1.27**	**8.82**	**8.85**	**24.14**	**4.62**	**6.79**	**8.17**	**8.35**	**13.61**	**-2.85**	**0.00**
**Fold Change**	0.85	0.86	0.67	0.68	0.50	0.77	0.57	0.75	0.70	0.72	1.06	1.00

DRUG	Honokiol	PP121	GSK2126458	AS-604850	A-769662	Dacomitinib	CCT137690	CUDC-907	TAK-285	INK 128	Semaxanib	DMSO
**MEAN**	5512	2430	4362	6272	6657	6082	5447	10445	5065	2612	5365	5172
**SEM**	93	77	276	288	128	137	183	369	200	171	247	176
**SSMD**	**-5.01**	**28.53**	**2.42**	**-3.83**	**-11.74**	**-6.56**	**-1.22**	**-12.64**	**0.29**	**12.51**	**-1.38**	**0.00**
**Fold Change**	1.07	0.47	0.84	1.21	1.29	1.19	1.06	2.03	0.99	0.50	1.04	1.00

DRUG	Indirubin	OSI-027	WYE-125132	CAY10505	CH5132799	AG-1478	AZ 628	NVP-BVU972	Tofacitinib	BYL719	Golvatinib	DMSO
**MEAN**	5300	3885	4602	5943	4522	6413	4143	7071	5882	5638	4041	6357
**SEM**	189	139	137	379	189	376	73	258	376	350	219	193
**SSMD**	**4.87**	**16.16**	**11.40**	**1.99**	**9.48**	**0.60**	**26.94**	**-1.99**	**1.15**	**3.08**	**9.65**	**0.00**
**Fold Change**	0.83	0.61	0.68	1.04	0.71	1.11	0.66	1.12	0.92	0.90	0.64	1.00

DRUG	Quercetin	Fostamatinib	A-674563	CHIR-124	KX2-391	SB415286	AMG-458	3-Methyl adenine	Sotrastaurin	Tyrphostin AG 879	IMD 0354	DMSO
**MEAN**	5992	4446	3715	5346	7597	7619	6991	6389	5821	5121	3974	5379
**SEM**	134	191	131	159	363	477	494	189	96	337	276	142
**SSMD**	**-5.07**	**4.20**	**11.36**	**0.10**	**-6.16**	**-3.83**	**-2.49**	**-5.30**	**-4.15**	**0.57**	**4.73**	**0.00**
**Fold Change**	1.12	0.83	0.69	1.00	1.41	1.41	1.30	1.19	1.08	0.95	0.74	1.00

DRUG	WHI-P154	GNF-2	Pazopanib	AP26113	CX-6258 HCl	IPA-3	AZD1080	RKI-1447	Ro3280	ZCL278	GNE-0877	DMSO
**MEAN**	4805	7768	5132	2687	6287	6074	6671	5283	5570	6434	6615	5760
**SEM**	219	231	294	99	314	309	364	390	502	362	466	171
**SSMD**	**3.75**	**-7.30**	**2.04**	**26.94**	**-0.61**	**-0.44**	**-2.02**	**1.72**	**1.00**	**-0.38**	**-1.00**	**0.00**
**Fold Change**	0.84	1.36	0.89	0.47	1.09	1.06	1.16	0.92	0.97	1.12	1.16	1.00

DRUG	TG100713	S-Ruxolitinib	Piceatannol	MEK162	AZ20	PF-3758309	10058-F4	BIO	AZD2858	AZD9291	GNE-9605	DMSO
**MEAN**	4228	6720	6161	4297	4600	2350	4347	6257	6226	3947	4791	5100
**SEM**	189	1687	302	50	158	111	237	231	215	238	260	128
**SSMD**	**4.22**	**0.02**	**-3.46**	**14.11**	**3.24**	**21.24**	**2.71**	**-3.87**	**-5.19**	**4.24**	**1.18**	**0.00**
**Fold Change**	0.83	1.30	1.22	0.85	0.90	0.46	0.85	1.23	1.23	0.78	0.94	1.00

DRUG	GW5074	PF-477736	Cabozantinib	PP2	CGI1746	VE-822	LY2835219	CNX-2006	NMS-P937	WZ4003	Sorafenib	DMSO
**MEAN**	4783	5139	4649	3764	3789	3286	4552	2362	5009	5712	3943	5259
**SEM**	276	301	298	152	217	205	429	95	102	379	161	111
**SSMD**	**1.49**	**1.12**	**2.22**	**8.87**	**5.84**	**8.54**	**2.10**	**25.73**	**1.89**	**-0.45**	**6.35**	**0.00**
**Fold Change**	0.91	0.98	0.88	0.71	0.72	0.62	0.86	0.45	0.95	1.09	0.75	1.00

DRUG	IKK-16	Go 6983	JNK-IN-8	VS-5584	PP1	AZD1208	SSR128129E	Ro 31-8220	CNX-774	EHop-016	KN-62	DMSO
**MEAN**	3638	6170	6187	3263	4926	5348	5676	4107	5132	4481	5018	5008
**SEM**	180	314	237	144	132	83	140	312	269	275	142	133
**SSMD**	**6.03**	**-2.41**	**-4.40**	**10.54**	**0.15**	**-3.76**	**-4.19**	**2.52**	**0.06**	**1.80**	**0.07**	**0.00**
**Fold Change**	0.73	1.24	1.24	0.65	0.99	1.07	1.14	0.83	1.03	0.90	1.01	1.00

DRUG	PF-562271	BAY11-7082	SC-514	CZC24832	MK-8745	AZD3463	AVL-292	GF109203X	PD173955	TG003	KN-93	DMSO
**MEAN**	3304	5868	4750	4942	5154	2931	4313	5597	4847	5568	5961	4953
**SEM**	155	88	120	135	132	178	265	385	382	294	461	217
**SSMD**	**9.44**	**-10.07**	**1.21**	**-0.97**	**-2.32**	**9.94**	**2.70**	**-1.07**	**0.00**	**-2.91**	**-1.43**	**0.00**
**Fold Change**	0.67	1.20	0.97	1.00	1.05	0.60	0.87	1.13	0.98	1.13	1.21	1.00

DRUG	Tyrphostin 9	Icotinib	Tofacitinib	IPI-145	LDK378	NU6027	SKI II	GSK650394	CO-1686	6H05	AR-A014418	DMSO
**MEAN**	3913	6492	6006	5357	3644	6592	6081	7446	3379	4939	5196	4739
**SEM**	209	625	150	109	134	624	237	363	109	268	370	194
**SSMD**	**3.31**	**-1.63**	**-7.49**	**-6.04**	**6.28**	**-1.95**	**-5.06**	**-6.42**	**9.92**	**-0.40**	**-1.46**	**0.00**
**Fold Change**	0.83	1.37	1.27	1.14	0.78	1.42	1.29	1.58	0.72	1.05	1.11	1.00

DRUG	ZM 323881	CHIR-99021	Fingolimod	XL019	GSK2334470	TIC10	PF-543	Skepinone-L	PFK15	K-Ras(G12C) inhibitor 9	LDC000067	DMSO
**MEAN**	3810	12848	4858	4604	3845	5633	4486	4953	6100	7433	5421	5201
**SEM**	144	2121	514	102	165	359	236	335	483	386	331	128
**SSMD**	**8.86**	**-3.47**	**0.44**	**5.50**	**7.19**	**-0.75**	**3.17**	**1.86**	**-0.94**	**-4.44**	**-0.46**	**0.00**
**Fold Change**	0.74	2.49	0.94	0.89	0.74	1.08	0.86	0.96	1.17	1.43	1.04	1.00

DRUG	ZM 306416	TAK-715	VX-702	PD168393	Zotarolimus	CGK 733	GZD824	Losmapimod	TAK-632	HS-173	EHT 1864	DMSO
**MEAN**	5812	5428	5199	5677	5972	7626	3226	5093	3529	4356	4922	5106
**SEM**	218	237	205	136	464	384	88	168	185	135	295	173
**SSMD**	**-2.67**	**-0.53**	**-1.06**	**-3.37**	**-1.10**	**-5.48**	**17.41**	**-1.14**	**7.15**	**4.86**	**0.71**	**0.00**
**Fold Change**	1.14	1.06	1.02	1.12	1.17	1.50	0.63	1.00	0.69	0.86	0.96	1.00

DRUG	JNK Inhibitor IX	XMD8-92	GNF-5	GNE-7915	WH-4-023	IM-12	Filgotinib	GSK2636771	PQ 401	ZM 39923	SMI-4a	VE-821
**MEAN**	5083	4828	8264	5322	4850	7451	5101	5985	5709	4668	5332	5282
**SEM**	332	134	213	180	203	308	237	201	127	283	144	428
**SSMD**	**-0.18**	**1.66**	**-13.31**	**-1.63**	**0.54**	**-7.31**	**-0.16**	**-4.23**	**-5.68**	**0.41**	**-2.31**	**-0.66**
**Fold Change**	1.02	0.96	1.65	1.06	0.97	1.49	1.02	1.20	1.14	0.93	1.07	1.06

DRUG	AG-18	CEP-32496	AZD5363	TCS 359	Tyrphostin AG 1296	NSC 23766	PRT062607	Butein	GDC-0349	BMS-345541	ETP-46464	Pacritinib
**MEAN**	5153	2847	3269	5537	4863	4728	2522	5375	3896	4945	3491	2729
**SEM**	205	212	167	652	217	164	204	359	443	338	101	251
**SSMD**	**-0.59**	**8.37**	**8.20**	**-0.17**	**0.84**	**0.97**	**10.72**	**-0.20**	**2.78**	**0.39**	**12.32**	**8.22**
**Fold Change**	1.03	0.57	0.65	1.11	0.97	0.95	0.50	1.07	0.78	0.99	0.70	0.55

DRUG	P276-00	Bardoxolone
**MEAN**	2624	6889
**SEM**	43	32
**SSMD**	**48.11**	**-51.01**
**Fold Change**	0.52	1.38

Supplemental Table S2: Secondary Screen

	0.5 μM				
	non-ARE		ARE		
DRUG	*AVE*	*SEM*	*AVE*	*SEM*	*P*
DMSO control	0.98	0.15	0.93	0	0.7633
AT9283	0.97	0.06	0.83	0.05	0.1527
AT7867	0.82	0.07	0.58	0.07	0.0708
Torin 2	0.96	0.16	0.97	0.02	0.9373
Ibrutinib (PCI-32765)	0.48	0.03	0.54	0.03	0.2034
AZ 628	1.3	0.17	1	0.05	0.1731
PF-562271	0.99	0.22	0.59	0.01	0.1465
Cediranib (AZD2171)	1.26	0.03	1.02	0.17	0.2405
WZ8040	1.03	0.03	1.21	0.19	0.4072
AZD1480	0.99	0.13	0.9	0.03	0.5613
DCC-2036 (Rebastinib)	1.04	0.03	0.86	0.1	0.1635
GDC-0980 (RG7422)	0.77	0.05	0.78	0.02	0.9284
Golvatinib (E7050)	1.01	0.03	0.74	0.03	0.0039
AZD3463	0.98	0.04	0.97	0.12	0.8977
Sorafenib Tosylate	0.9	0.05	0.58	0.05	0.0106
Crizotinib (PF-02341066)	0.85	0.05	0.58	0.02	0.0084
Ponatinib (AP24534)	1.01	0.13	0.94	0.07	0.6833
TAK-901	0.79	0	0.64	0.1	0.1997
PF-04691502	0.66	0.04	0.63	0.06	0.6959
A-674563	0.69	0.04	0.65	0	0.4291
CO-1686 (AVL-301)	0.95	0.04	0.84	0.03	0.1208
SB203580	1	0.04	0.79	0.02	0.0088
BMS-754807	1.09	0.09	1.01	0.03	0.4738
TAE226 (NVP-TAE226)	0.93	0.17	0.91	0.01	0.9018
AZD2014	0.75	0.02	0.64	0.04	0.074
AP26113	0.46	0.33	0.59	0.15	0.7422
ZM 323881 HCl	0.5	0.03	0.49	0.03	0.8189
Palbociclib (PD-0332991) HCl	1.2	0.12	0.84	0.04	0.0458
BMS-536924	1.18	0.04	0.95	0.09	0.0869
ENMD-2076	0.96	0.05	0.9	0.15	0.7219
AZD7762	1.11	0.37	0.75	0.09	0.3994
GDC-0068	0.97	0.25	1.1	0.12	0.6826
PF-3758309	1.19	0.23	1.02	0	0.504
GSK2334470	0.72	0.02	0.47	0.02	0.0004

**Sunitinib Malate**	0.96	0.02	0.71	0.06	0.0201
**PI-103**	1.07	0.16	0.93	0.09	0.4844
**AT7519**	1.03	0.09	0.88	0.11	0.3515
**KU-0063794**	0.9	0.14	0.76	0.11	0.4448
**CCT128930**	0.82	0.15	0.78	0.02	0.7836
**PP2**	1.16	0.1	1.15	0.06	0.9151
**GZD824**	1	0.08	1.11	0.13	0.5229
**Nilotinib (AMN-107)**	0.86	0	0.48	0.02	0
**Y-27632 2HCl**	1.04	0.02	0.87	0.09	0.133
**KW-2449**	1.09	0.05	1.02	0.04	0.2915
**Volasertib (BI 6727)**	1.14	0.03	0.71	0.03	0.0006
**Trametinib (GSK1120212)**	1.09	0.27	1.31	0.06	0.4736
**PP121**	1.02	0.08	0.82	0.13	0.2423
**CGI1746**	0.6	0	0.57	0.04	0.6164
**TAK-632**	1.28	0	0.93	0.07	0.0081
**Danusertib (PHA-739358)**	1.36	0.12	0.7	0.01	0.0054
**ZSTK474**	1.03	0.17	0.98	0.15	0.8362
**PP242**	1.07	0.13	0.92	0.15	0.5048
**NVP-BSK805 2HCl**	0.83	0.07	0.69	0.08	0.2816
**TG101348 (SAR302503)**	1.26	0.31	1.02	0.06	0.4913
**INK 128 (MLN0128)**	1.04	0.01	1.02	0.01	0.1993
**VE-822**	0.68	0.05	0.62	0.11	0.6362
**CEP-32496**	0.96	0.06	1.07	0	0.1319
**SNS-032 (BMS-387032)**	1.14	0.09	0.63	0.04	0.0079
**Refametinib (RDEA119, Bay 86-9766)**	1.01	0.08	0.67	0.01	0.0111
**PIK-75**	0.8	0.08	0.74	0.02	0.4937
**GSK1838705A**	0.63	0.03	0.68	0.04	0.3131
**Flavopiridol HCl**	0.85	0.09	0.91	0.06	0.5764
**OSI-027**	0.87	0.03	0.58	0.15	0.129
**CNX-2006**	0.85	0.08	0.75	0.01	0.2874
**AZD5363**	1.15	0.23	1.05	0.07	0.7023
**BI 2536**	0.9	0.03	0.65	0.02	0.0022
**Regorafenib (BAY 73-4506)**	0.87	0.02	0.64	0.05	0.0128
**Hesperadin**	0.92	0.09	0.68	0.04	0.0721
**BGT226 (NVP-BGT226)**	0.67	0.17	0.64	0.02	0.8608
**TG101209**	0.47	0.06	0.49	0.05	0.8169
**WYE-125132 (WYE-132)**	0.86	0.09	0.88	0.08	0.8689
**IKK-16 (IKK Inhibitor VII)**	0.63	0.06	0.64	0.01	0.8933

**PRT062607 (P505-15, BIIB057) HCl**	1.17	0.41	1.13	0.11	0.9288
**Cabozantinib (XL184, BMS-907351)**	0.92	0.06	0.77	0.12	0.324
**OSI-906 (Linsitinib)**	1.31	0.15	1.14	0.03	0.3257
**SGI-1776 free base**	1	0.04	0.92	0.04	0.2435
**Dinaciclib (SCH727965)**	1.09	0.14	0.97	0.03	0.4508
**NU7441 (KU-57788)**	0.81	0.05	0.81	0.06	0.9266
**CH5132799**	0.92	0.19	0.98	0.07	0.7776
**VS-5584 (SB2343)**	0.77	0.03	0.8	0.03	0.452
**ETP-46464**	0.71	0.01	0.74	0.01	0.1065
**Milciclib (PHA-848125)**	0.85	0.13	0.68	0.08	0.313
**CCT137690**	0.16	0.96	0.93	0.25	0.48
**CHIR-124**	0.06	1.04	0.97	0.06	0.4305
**CX-6258 HCl**	0.14	1.27	1.62	0.05	0.3063
**CNX-774**	0.12	1.21	1.01	0.15	0.5038
**Tyrphostin AG 1296**	0.08	1.19	1.09	0.12	0.449
**P276-00**	0.23	1.52	0.95	0.16	0.6639
**Pacritinib (SB1518)**	0.13	0.97	0.81	0	0.5267

Supplemental Table S3

GeneID	Symbol	Length	DMSO-Expression	PLK1 inhibitor-E	log2FoldCha	Padj	Up/Down-	Fold
51166	AADAT	2167	234.8727612	97.7055394	-1.2653671	6.89E-06	Down	0.415993
3983	ABLIM1	6804	1853.188812	1073.281516	-0.7879813	1.01E-09	Down	0.579154
8309	ACOX2	2356	128.1355282	54.90384016	-1.2226916	0.001573	Down	0.428483
51703	ACSL5	3372	4588.124886	2297.488984	-0.9978467	1.59E-20	Down	0.500747
8728	ADAM19	6554	1228.296036	578.6359916	-1.0859303	8.99E-09	Down	0.471088
10863	ADAM28	5102	175.7165015	87.84451076	-1.0002256	0.001356	Down	0.499922
56999	ADAMTS9	7335	866.0704115	475.9371054	-0.8637134	3.56E-07	Down	0.549536
11033	ADAP1	2367	53.44223955	15.69740859	-1.7674541	0.000607	Down	0.293727
134637	ADAT2	6746	235.6755715	135.5944761	-0.7975038	0.004401	Down	0.575344
109	ADCY3	4743	602.148389	270.8873043	-1.1524263	1.77E-10	Down	0.449868
113	ADCY7	6100	831.6621113	397.0802583	-1.0665669	2.16E-10	Down	0.477454
266977	ADGRF1	4945	2895.812125	1110.065398	-1.3833233	3.44E-34	Down	0.383335
22859	ADGRL1	7859	699.0836487	376.9099143	-0.8912453	1.89E-06	Down	0.539149
154	ADRB2	2058	1269.213955	703.098932	-0.8521357	2.71E-09	Down	0.553964
64782	AEN	3134	672.4269392	252.8638357	-1.4110168	4.38E-16	Down	0.376047
134265	AFAP1L1	6005	353.1402397	191.8564908	-0.8802136	0.000211	Down	0.543287
79814	AGMAT	3154	138.7988132	66.5399	-1.0607036	0.004513	Down	0.479398
9447	AIM2	1558	69.15865834	29.30862536	-1.2385846	0.008104	Down	0.423788
9590	AKAP12	8246	896.0924152	328.1472811	-1.449304	8.93E-20	Down	0.366198
220	ALDH1A3	3539	496.3417068	211.5962195	-1.2300198	4.11E-10	Down	0.426312
139285	AMER1	8443	158.097466	76.00416446	-1.0566639	0.002063	Down	0.480742
287	ANK2	6893	487.1447174	284.2322719	-0.7772801	0.0001	Down	0.583466
118932	ANKRD22	3993	41.28143661	12.47206801	-1.7267925	0.002237	Down	0.302123
55107	ANO1	4811	28.97378757	5.214802235	-2.4740638	3.08E-05	Down	0.179983
10541	ANP32B	1617	7230.187724	4137.437837	-0.8052955	6.53E-16	Down	0.572245
81611	ANP32E	3466	4617.093395	2254.817796	-1.0339741	2.88E-16	Down	0.488363
653145	ANXA8	2070	413.3262084	135.0983473	-1.6132708	0.00123	Down	0.326856
728113	ANXA8L1	2058	206.4494232	76.18687131	-1.4381741	2.62E-06	Down	0.369034
316	AOX1	4949	1021.295888	380.4966911	-1.4244451	1.56E-16	Down	0.372563
9582	APOBEC3	1560	564.082246	279.9910971	-1.0105246	9.23E-07	Down	0.496366
57514	ARHGAP3	8096	674.753049	382.6052206	-0.818503	3.23E-05	Down	0.56703
1E+08	ARMCX4	7424	129.7638654	59.11585212	-1.1342717	0.001588	Down	0.455565
9915	ARNT2	6576	785.3522963	362.7932996	-1.1141922	4.07E-10	Down	0.46195
51676	ASB2	2758	1466.268481	664.121446	-1.1426303	1.84E-10	Down	0.452933
29028	ATAD2	5580	3468.750054	1742.298348	-0.9934242	1.12E-16	Down	0.502284
79915	ATAD5	6877	502.857409	274.4222494	-0.8737519	2.02E-05	Down	0.545726
10331	B3GNT3	2720	104.6186182	39.8217613	-1.3935107	0.000417	Down	0.380637
580	BARD1	5175	565.0015063	308.2691458	-0.8740642	3.76E-06	Down	0.545608
8412	BCAR3	3199	3020.070573	1753.406755	-0.7844216	5.56E-11	Down	0.580585
586	BCAT1	9416	1887.985457	962.284022	-0.972313	7.12E-13	Down	0.509688
596	BCL2	6492	212.1296066	89.77005315	-1.2406398	2.75E-05	Down	0.423185
56898	BDH2	2936	274.0954849	142.8650988	-0.940025	0.000226	Down	0.521224
146227	BEAN1	2864	85.43724281	38.40517888	-1.1535642	0.008943	Down	0.449513
641	BLM	4597	477.7347085	277.3060043	-0.7847308	0.000117	Down	0.58046
168667	BMPER	5031	123.1304111	61.53561181	-1.0006937	0.006409	Down	0.49976

672	BRCA1	5099	1022.062915	499.8215299	-1.0319991	6.49E-10	Down	0.489032
83990	BRIP1	8166	1367.635933	504.1751668	-1.4396873	6.73E-21	Down	0.368647
55299	BRIX1	1303	1741.471913	980.8826209	-0.8281548	2.89E-09	Down	0.563249
84798	C19orf48	1545	1872.465467	1057.756072	-0.8239321	2.01E-10	Down	0.5649
114897	C1QTNF1	2868	93.95787376	39.71661337	-1.2422715	0.003672	Down	0.422707
55286	C4orf19	3647	186.2090416	75.74295378	-1.2977395	2.20E-05	Down	0.406763
135398	C6orf141	1696	115.5808256	48.71670482	-1.2464136	0.001048	Down	0.421495
785	CACNB4	7881	63.92943524	22.34620996	-1.5164502	0.001814	Down	0.349545
29775	CARD10	3912	608.2484233	345.4387693	-0.8162307	6.35E-06	Down	0.567924
84733	CBX2	4265	315.1407543	170.9507975	-0.8824152	0.000226	Down	0.542459
57545	CC2D2A	2950	593.8510104	306.7657971	-0.9529634	1.26E-07	Down	0.51657
91050	CCDC149	4041	272.1376878	159.9641912	-0.7665878	0.003449	Down	0.587806
80071	CCDC15	3897	193.7112702	95.87677033	-1.0146547	0.000751	Down	0.494947
339512	CCDC190	1552	58.31386684	18.65040079	-1.6446324	0.00088	Down	0.319828
91057	CCDC34	1493	540.9554036	305.1918056	-0.8257934	0.000166	Down	0.564172
388115	CCDC9B	5344	2397.520361	1386.734592	-0.7898514	1.25E-08	Down	0.578404
898	CCNE1	1948	319.3411397	117.9950354	-1.4363723	4.47E-09	Down	0.369495
9134	CCNE2	2739	818.8507731	236.4053867	-1.7923377	2.34E-22	Down	0.288704
9738	CCP110	5481	664.5571615	369.2617534	-0.8477495	1.16E-05	Down	0.555651
29126	CD274	3398	651.3059033	297.4872441	-1.1305075	3.38E-10	Down	0.456755
10849	CD3EAP	3288	682.1461479	390.7121591	-0.8039747	0.000314	Down	0.572769
9308	CD83	2345	406.327293	173.3237266	-1.2291731	1.82E-08	Down	0.426562
993	CDC25A	3717	755.2290156	271.9982407	-1.4733169	1.75E-16	Down	0.360153
55561	CDC42BP	4945	146.8164524	63.63806122	-1.2060519	0.000413	Down	0.433453
8318	CDC45	1998	1013.302184	338.1603445	-1.5832851	2.45E-16	Down	0.333721
990	CDC6	3053	2274.143187	1102.22957	-1.0448984	1.48E-18	Down	0.484679
8317	CDC7	3237	1041.111262	612.9646757	-0.7642484	5.27E-06	Down	0.58876
55038	CDCA4	2453	1489.680504	865.6961545	-0.7830703	1.61E-06	Down	0.581129
113130	CDCA5	2599	2736.1209	1565.880792	-0.8051576	1.06E-06	Down	0.5723
83879	CDCA7	2638	538.4769099	174.7486157	-1.6236035	3.79E-14	Down	0.324524
1031	CDKN2C	2039	1367.636074	725.7508369	-0.9141381	1.30E-07	Down	0.530661
81620	CDT1	2742	1232.034521	477.4632869	-1.367581	4.70E-20	Down	0.387541
57214	CEMIP	7140	951.5282138	538.8710439	-0.8203064	0.000144	Down	0.566322
55166	CENPQ	1753	618.072452	357.119762	-0.791368	2.85E-05	Down	0.577796
79682	CENPU	2554	2196.84006	1159.170772	-0.9223367	3.63E-12	Down	0.527654
10036	CHAF1A	3344	2258.961307	1116.607921	-1.0165369	9.61E-18	Down	0.494301
8208	CHAF1B	2297	637.8133187	348.7189807	-0.8710693	1.14E-05	Down	0.546741
1122	CHML	7082	1301.726935	608.562862	-1.0969486	1.97E-15	Down	0.467504
9469	CHST3	6978	936.6806617	481.8750715	-0.9588981	4.55E-08	Down	0.51445
4261	CIITA	4029	397.1299918	193.5815755	-1.0366697	1.64E-06	Down	0.487451
51192	CKLF	836	1332.977052	744.4599883	-0.8403857	1.11E-08	Down	0.558494
9076	CLDN1	3452	87.06663304	40.27668731	-1.1121749	0.009111	Down	0.462596
54102	CLIC6	4048	236.7736352	132.4420346	-0.8381474	0.003866	Down	0.559361
79827	CLMP	2955	537.7161358	186.8129851	-1.52525	2.58E-14	Down	0.347419
63967	CLSPN	7092	1988.072128	958.4011548	-1.0526685	2.45E-15	Down	0.482076
22866	CNKSR2	5554	115.7839639	52.17899455	-1.1498944	0.002288	Down	0.450658
22837	COBLL1	9374	97.47659058	36.80299174	-1.4052327	0.000675	Down	0.377557

1306	COL15A1	5422	57.2707228	19.2529374	-1.5727192	0.00201	Down		0.336174
1307	COL16A1	5584	71.90967548	29.84609702	-1.2686436	0.005518	Down		0.41505
1308	COL17A1	5610	457.3825243	204.6041095	-1.1605661	4.27E-07	Down		0.447337
85301	COL27A1	7818	480.1816288	235.9088966	-1.0253504	5.75E-07	Down		0.491291
1282	COL4A1	6545	1104.906097	649.0522697	-0.7675172	2.73E-05	Down		0.587428
1294	COL7A1	9169	2515.902625	1180.785951	-1.0913286	6.17E-21	Down		0.469329
118881	COMTD1	1344	274.3236155	154.8571385	-0.8249409	0.001546	Down		0.564505
1436	CSF1R	3986	112.8303978	29.27001301	-1.9466605	4.77E-07	Down		0.259416
1437	CSF2	800	1902.767677	614.67142	-1.6302121	2.73E-05	Down		0.323041
1440	CSF3	1595	1065.725428	97.58542336	-3.4490263	2.65E-63	Down		0.091567
55790	CSGALNA	3790	213.0573874	69.95849799	-1.6066709	1.93E-06	Down		0.328355
1520	CTSS	4107	472.0012263	236.3394249	-0.9979303	1.97E-05	Down		0.500718
6376	CX3CL1	3220	65.98117498	23.28333552	-1.5027567	0.002553	Down		0.352878
2919	CXCL1	1184	3821.991721	1417.049983	-1.431434	0.002949	Down		0.370762
6373	CXCL11	1603	11.52337704	3.388864311	-1.7656898	0.005602	Down		0.294086
2920	CXCL2	1234	300.7356758	83.77978846	-1.8438219	8.88E-13	Down		0.278583
2921	CXCL3	1166	380.6830198	90.16742417	-2.077912	6.46E-15	Down		0.236857
3576	CXCL8	1666	2306.773642	660.238618	-1.804817	3.00E-07	Down		0.286217
56603	CYP26B1	4394	329.643413	159.5359265	-1.0470249	3.57E-05	Down		0.483965
1577	CYP3A5	1935	72.4658986	19.84947571	-1.8682013	0.000183	Down		0.273915
1551	CYP3A7	2099	18.1934337	5.341288809	-1.7681581	0.006107	Down		0.293583
1633	DCK	2618	1077.535698	607.3561196	-0.8271211	3.43E-07	Down		0.563653
9937	DCLRE1A	4369	641.9996943	325.2513319	-0.9810176	3.98E-08	Down		0.506622
64858	DCLRE1B	3772	782.0019687	457.6493309	-0.7729297	2.05E-06	Down		0.585228
1643	DDB2	1846	478.7148119	268.4091575	-0.8347326	8.69E-05	Down		0.560687
55601	DDX60	6099	258.6978444	127.096154	-1.0253477	0.000171	Down		0.491292
50619	DEF6	2320	125.3069523	46.98648993	-1.4151486	0.000155	Down		0.374971
79961	DENND2I	3228	172.0486106	82.13961173	-1.0666662	0.001068	Down		0.477421
1719	DHFR	3929	4752.550962	2684.566597	-0.8240129	4.34E-12	Down		0.564869
1755	DMBT1	6205	114.2407304	44.26691449	-1.3677764	0.000464	Down		0.387488
1763	DNA2	4287	491.543707	224.5327994	-1.1303935	2.88E-08	Down		0.456791
79962	DNAJC22	4161	102.0242747	41.75119036	-1.2890232	0.001232	Down		0.409228
55619	DOCK10	7288	396.9799911	191.9829649	-1.048088	1.61E-06	Down		0.483609
1794	DOCK2	6117	492.8688746	245.9525925	-1.0028236	1.09E-06	Down		0.499022
1824	DSC2	5235	465.1955063	245.9279772	-0.9196013	0.000153	Down		0.528655
79075	DSCC1	2291	480.523214	231.137094	-1.0558573	3.13E-07	Down		0.481011
79980	DSN1	2240	1479.961626	763.024477	-0.9557585	4.70E-12	Down		0.51557
51514	DTL	4588	1927.89265	779.5963705	-1.3062254	4.48E-23	Down		0.404377
1854	DUT	1942	1383.59889	698.6409644	-0.9858026	8.21E-11	Down		0.504945
1869	E2F1	2722	2584.575644	1186.838842	-1.1228034	6.89E-22	Down		0.459201
1870	E2F2	5201	264.8830192	45.54569012	-2.5399689	6.90E-19	Down		0.171946
79733	E2F8	3551	744.468736	129.0951729	-2.5277762	1.16E-38	Down		0.173406
55268	ECHDC2	1773	309.4207942	157.829259	-0.9712055	4.44E-05	Down		0.51008
1906	EDN1	2109	389.8089829	184.2976084	-1.08073	1.54E-06	Down		0.47279
1907	EDN2	1168	34.16915326	11.04737183	-1.6289913	0.005338	Down		0.323314
1917	EEF1A2	2022	256.489251	143.9151279	-0.8336801	0.004909	Down		0.561096
114794	ELFN2	8379	626.7209711	356.7168941	-0.8130437	1.07E-05	Down		0.56918

133418	EMB	4309	1129.693614	662.2287968	-0.7705299	6.62E-06	Down	0.586202
285203	EOGT	4666	633.9575038	337.6966116	-0.9086584	4.46E-07	Down	0.53268
4072	EPCAM	1731	433.8141019	229.98039	-0.9155661	8.33E-06	Down	0.530136
2051	EPHB6	3380	57.27191975	19.69620723	-1.5399101	0.003907	Down	0.343907
253152	EPHX4	1443	291.221284	164.5692269	-0.8234212	0.00211	Down	0.5651
157570	ESCO2	3376	568.8726989	302.167839	-0.9127557	8.55E-06	Down	0.53117
9700	ESPL1	6641	1425.769013	703.9467553	-1.0182021	1.30E-08	Down	0.493731
80004	ESRP2	4021	278.6267074	139.2364492	-1.0007966	6.56E-05	Down	0.499724
2118	ETV4	2318	1428.259513	733.7989093	-0.9608015	8.16E-12	Down	0.513771
2123	EVI2A	2890	585.2478992	279.3406937	-1.0670221	1.81E-08	Down	0.477303
9156	EXO1	3322	1073.008329	404.7024516	-1.4067278	7.70E-18	Down	0.377166
5393	EXOSC9	1619	1465.706991	792.0595221	-0.887916	1.65E-08	Down	0.540394
2146	EZH2	2670	2470.977137	1327.867503	-0.8959705	3.72E-13	Down	0.537386
9002	F2RL3	3467	84.14854817	25.83806335	-1.7034404	8.95E-05	Down	0.307053
55179	FAIM	1151	297.3299041	173.8645441	-0.7741008	0.001708	Down	0.584753
63901	FAM111A	3712	2312.126065	875.1897284	-1.4015523	1.05E-28	Down	0.378522
374393	FAM111B	3450	2103.763592	520.8531397	-2.014024	1.73E-39	Down	0.247582
84908	FAM136A	1866	1806.05535	1054.194912	-0.7767005	5.19E-08	Down	0.5837
727866	FAM156B	3222	507.7826717	291.483792	-0.8007955	0.000127	Down	0.574033
83648	FAM167A	4093	163.2009191	61.83219331	-1.4002191	1.55E-05	Down	0.378872
79632	FAM184A	4045	38.91417363	13.11586087	-1.5689832	0.00542	Down	0.337046
1E+08	FAM196B	5962	396.6283168	117.8473746	-1.7508681	9.51E-14	Down	0.297123
131583	FAM43A	3182	1963.230511	1084.436378	-0.8562842	4.74E-08	Down	0.552373
145773	FAM81A	3468	78.76410837	31.5695388	-1.3190052	0.002816	Down	0.400811
84985	FAM83A	4033	484.4105536	142.056224	-1.7697683	2.75E-13	Down	0.293256
2175	FANCA	5183	1216.450317	683.3275443	-0.8320282	2.54E-08	Down	0.561739
2177	FANCD2	5187	813.3678757	462.3950274	-0.8147821	1.03E-05	Down	0.568494
2189	FANCG	2649	1183.04733	646.1877521	-0.8724825	1.28E-08	Down	0.546206
55215	FANCI	4749	2069.520204	1132.661224	-0.8695799	3.11E-09	Down	0.547306
355	FAS	3951	118.5152491	59.34596056	-0.997851	0.007357	Down	0.500745
120114	FAT3	19048	124.6652723	23.70280941	-2.3949297	3.68E-10	Down	0.190132
10826	FAXDC2	3046	324.8529073	177.9313769	-0.8684657	0.000329	Down	0.547729
2203	FBP1	1558	223.7190818	81.26945872	-1.4609031	1.60E-07	Down	0.363266
26271	FBXO5	2174	964.2537524	456.3148948	-1.0793831	2.98E-11	Down	0.473231
2237	FEN1	2308	4139.515897	2084.968343	-0.9894366	4.74E-18	Down	0.503674
55612	FERMT1	5167	368.374774	155.8670875	-1.2408579	1.98E-08	Down	0.423121
2246	FGF1	3818	82.8676803	26.71983553	-1.6328984	0.000346	Down	0.32244
55137	FIGN	7210	214.3826017	123.7591481	-0.7926527	0.006115	Down	0.577282
24147	FJX1	2701	1381.476516	611.7090951	-1.1752934	4.49E-17	Down	0.442794
342184	FMN1	12564	606.7953483	355.9986839	-0.7693381	0.000104	Down	0.586687
752	FMNL1	3973	4563.667447	2366.901071	-0.9471943	3.59E-13	Down	0.51864
2296	FOXC1	3452	322.2484821	182.3378941	-0.8215591	0.000666	Down	0.56583
55691	FRMD4A	6131	1220.630343	665.9762474	-0.8740837	6.27E-08	Down	0.5456
10468	FST	1863	181.5996888	62.61884804	-1.5360929	7.75E-07	Down	0.344818
8321	FZD1	4350	1736.513435	997.4001922	-0.7999492	6.87E-08	Down	0.574369
2591	GALNT3	3280	1033.402171	444.3936547	-1.2174917	2.89E-10	Down	0.43003
8200	GDF5	2586	50.29128332	19.37899155	-1.3758149	0.009141	Down	0.385335

79017	GGCT	1197	682.5062107	389.7937959	-0.808131	4.66E-05	Down	0.571121
2687	GGT5	2462	720.1510523	417.9520769	-0.784962	2.11E-05	Down	0.580367
9837	GINS1	3293	766.301442	386.0888272	-0.9889792	7.24E-08	Down	0.503834
51659	GINS2	1196	448.161661	128.7631868	-1.799299	6.22E-17	Down	0.287314
64785	GINS3	2296	297.4352299	145.1950873	-1.0345829	2.85E-05	Down	0.488157
2697	GJA1	3169	408.7611267	183.6164223	-1.1545629	1.36E-07	Down	0.449202
2706	GJB2	2347	753.3135729	247.2028777	-1.607555	0.000137	Down	0.328154
11010	GLIPR1	3924	2538.184616	1392.358274	-0.8662665	3.34E-12	Down	0.548565
169792	GLIS3	7083	334.744764	161.376917	-1.0526273	7.28E-06	Down	0.482089
150763	GPAT2	2971	745.7114597	413.0874269	-0.8521704	2.82E-06	Down	0.553951
2827	GPR3	2149	211.7295506	104.5354434	-1.0182305	0.00053	Down	0.493722
8111	GPR68	2864	1395.77046	357.8007518	-1.9638334	7.50E-31	Down	0.256346
53836	GPR87	1521	43.03080511	14.81203587	-1.5385999	0.005518	Down	0.344219
2886	GRB7	2149	79.7505538	33.16569856	-1.2658026	0.004387	Down	0.415868
134266	GRPEL2	4105	503.7694925	292.2969709	-0.7853289	0.000127	Down	0.58022
54103	GSAP	3039	299.7846957	114.4327034	-1.3894273	2.46E-07	Down	0.381716
112495	GTF3C6	957	706.1244926	349.0866517	-1.0163374	1.88E-08	Down	0.49437
3037	HAS2	3275	384.8597661	114.1312711	-1.7536387	0.002909	Down	0.296553
3038	HAS3	3961	105.9527297	46.42349755	-1.1904936	0.002504	Down	0.438153
115106	HAUS1	1145	737.1756251	384.9635884	-0.9372864	2.09E-08	Down	0.522214
23354	HAUS5	4309	599.1714752	328.3531906	-0.8677205	1.54E-06	Down	0.548012
50810	HDGFL3	2446	457.7094275	181.9494243	-1.3308945	1.84E-10	Down	0.397522
3070	HELLS	3076	766.5658343	323.2843356	-1.2456061	6.73E-11	Down	0.421731
3280	HES1	1475	292.5702136	135.2702122	-1.1129387	8.56E-06	Down	0.462351
64399	HHIP	3555	105.8777216	45.87932427	-1.206483	0.002199	Down	0.433324
55355	HJURP	3189	2901.626951	1706.710344	-0.7656438	6.56E-06	Down	0.588191
80201	HKDC1	3719	272.2960012	90.3745296	-1.5911877	3.23E-08	Down	0.331898
3219	HOXB9	2711	291.2969568	129.9258842	-1.1648018	1.94E-06	Down	0.446026
10855	HPSE	4629	719.5469012	405.3448542	-0.8279389	3.02E-06	Down	0.563333
9957	HS3ST1	1965	594.7517287	313.0705611	-0.9257997	5.71E-07	Down	0.526389
10561	IFI44	1742	398.5909308	117.2658621	-1.7651258	2.45E-15	Down	0.294201
64135	IFIH1	3617	179.0357138	97.2291208	-0.880787	0.006901	Down	0.543071
3434	IFIT1	4425	549.4866558	243.7749828	-1.1725344	3.43E-08	Down	0.443641
7866	IFRD2	2129	2518.137076	1464.877979	-0.7815763	4.35E-11	Down	0.581731
57722	IGDCC4	6485	494.3088411	219.1408847	-1.173554	1.58E-08	Down	0.443328
10643	IGF2BP3	4168	1300.088377	417.5015118	-1.6387564	1.90E-24	Down	0.321133
3484	IGFBP1	1608	317.0280918	178.2801726	-0.8304644	0.002232	Down	0.562348
3589	IL11	2381	793.26584	257.0437227	-1.6257906	5.79E-17	Down	0.324032
3552	IL1A	2947	287.6786065	54.15496541	-2.4092924	1.86E-18	Down	0.188248
3553	IL1B	1498	304.8633295	103.0882206	-1.5642831	0.009177	Down	0.338146
9466	IL27RA	2685	295.1011238	127.5128208	-1.2105671	1.81E-06	Down	0.432099
3569	IL6	1197	336.8284069	96.57767302	-1.8022522	2.92E-13	Down	0.286727
3575	IL7R	4643	82.68727362	14.48167277	-2.513437	9.01E-08	Down	0.175138
3624	INHBA	6105	116.5955395	38.22076899	-1.6090839	3.29E-05	Down	0.327806
3654	IRAK1	3525	5379.898661	3130.949105	-0.7809789	4.62E-14	Down	0.581972
3669	ISG20	1758	139.016852	55.0111856	-1.3374629	0.000184	Down	0.395716
9358	ITGBL1	2658	76.62077237	32.48200759	-1.2380948	0.005504	Down	0.423932

3707	ITPKB	6162	159.1408943	49.29768401	-1.6907128	1.19E-06	Down	0.309774
56704	JPH1	4427	146.0338985	53.79653692	-1.4407181	1.86E-05	Down	0.368384
81621	KAZALD1	2734	203.0212674	111.4782764	-0.8648683	0.003275	Down	0.549097
3783	KCNN4	2240	760.2938203	335.0111962	-1.1823478	6.21E-11	Down	0.440634
9132	KCNQ4	3954	50.76507787	14.47199264	-1.8105728	0.000776	Down	0.285078
56479	KCNQ5	6500	926.2759451	498.7072321	-0.8932489	1.10E-08	Down	0.5384
143888	KDELC2	4311	2204.590739	1214.435806	-0.8602246	4.39E-10	Down	0.550867
57535	KIAA1324	6793	136.6939895	63.00670697	-1.1173725	0.001653	Down	0.460933
146909	KIF18B	4308	1648.470707	825.130547	-0.998434	6.32E-13	Down	0.500543
10112	KIF20A	3471	3030.507432	1762.026242	-0.782324	1.22E-06	Down	0.581429
151230	KLHL23	4089	436.2380649	186.8604346	-1.2231545	4.51E-07	Down	0.428345
26249	KLHL3	6806	87.24267739	29.29162473	-1.5745458	0.000187	Down	0.335749
57542	KLHL42	6477	517.7996387	276.2682879	-0.906324	4.61E-05	Down	0.533543
9735	KNTC1	6980	1882.646919	1029.744405	-0.8704762	5.12E-11	Down	0.546966
144501	KRT80	3867	4524.15297	2538.773727	-0.8335159	1.91E-15	Down	0.56116
144811	LACC1	4211	442.3419479	249.0547845	-0.8286989	5.94E-05	Down	0.563037
7462	LAT2	2010	76.05237598	26.05886434	-1.5452191	0.000572	Down	0.342644
51176	LEF1	2962	282.3219625	98.04446897	-1.5258332	9.41E-09	Down	0.347279
137994	LETM2	2598	279.5286688	136.3225967	-1.0359715	0.000212	Down	0.487687
3978	LIG1	3409	1802.324304	1029.366502	-0.8081019	1.71E-10	Down	0.571133
3985	LIMK2	3398	378.7851446	220.4334048	-0.7810369	0.000991	Down	0.581948
55679	LIMS2	1935	97.29338191	45.09560351	-1.1093549	0.006298	Down	0.463501
286826	LIN9	3147	453.4889506	258.0289015	-0.8135347	8.58E-05	Down	0.568986
4001	LMNB1	2806	3133.382686	1236.254903	-1.3417447	3.35E-21	Down	0.394543
389602	LOC38960	4584	440.5556187	216.986866	-1.0217164	5.42E-07	Down	0.49253
84695	LOXL3	2993	39.60475471	11.75479612	-1.7524241	0.001797	Down	0.296803
84171	LOXL4	3657	1105.824456	463.0558352	-1.2558643	2.73E-13	Down	0.418743
1902	LPAR1	3743	1927.697504	836.6637536	-1.2041588	1.12E-21	Down	0.434022
389816	LRRC26	1212	168.2501138	77.02680415	-1.127175	0.000524	Down	0.457811
201255	LRRC45	2741	577.9325278	340.8314139	-0.7618428	6.35E-05	Down	0.589743
85444	LRRCC1	3694	283.5191495	112.7446387	-1.3303874	1.75E-06	Down	0.397661
164312	LRRN4	2982	56.61576969	19.56115118	-1.5332127	0.002198	Down	0.345507
134353	LSM11	6591	357.9608267	171.817037	-1.0589286	1.44E-05	Down	0.479988
4053	LTBP2	8568	1420.464357	753.6356424	-0.9144235	1.61E-09	Down	0.530556
7851	MALL	3071	889.7205405	375.726575	-1.2436691	4.02E-13	Down	0.422297
79694	MANEA	4633	320.5916101	163.2042174	-0.9740583	3.13E-05	Down	0.509072
149175	MANEAL	2819	328.4772255	148.6426878	-1.1439449	1.09E-06	Down	0.452521
5603	MAPK13	6348	1922.656334	856.4286758	-1.1666959	4.14E-19	Down	0.44544
84930	MASTL	4670	1759.569391	1020.562205	-0.7858583	5.32E-07	Down	0.580007
55796	MBNL3	11272	573.5307865	204.2008088	-1.4898823	1.81E-15	Down	0.356042
154141	MBOAT1	4334	255.8238775	88.61124348	-1.5295893	2.00E-08	Down	0.346376
345643	MCIDAS	2104	87.40210838	29.14736375	-1.5843027	0.000285	Down	0.333486
55388	MCM10	4559	1359.587015	425.7649057	-1.6750395	7.50E-27	Down	0.313158
4171	MCM2	3504	2760.40065	807.150546	-1.773968	3.80E-44	Down	0.292403
4172	MCM3	3234	7522.465588	2667.170425	-1.4958956	3.47E-48	Down	0.354561
4173	MCM4	4800	4301.743022	2125.988339	-1.0167877	2.96E-19	Down	0.494216
4174	MCM5	2568	2237.642801	708.1475949	-1.6598578	1.83E-39	Down	0.31647

4175	MCM6	3791	1768.729218	557.7075271	-1.6651325	1.81E-34	Down	0.315315
4176	MCM7	3024	7950.554442	4513.302768	-0.8168719	4.45E-16	Down	0.567671
84515	MCM8	3763	1304.08107	725.6978845	-0.8455926	3.22E-09	Down	0.556482
79648	MCPH1	5800	449.1115624	241.6833404	-0.8939558	1.42E-05	Down	0.538137
29969	MDFIC	5226	893.1564078	446.4163469	-1.000523	3.30E-09	Down	0.499819
4212	MEIS2	3216	270.9978649	132.8600237	-1.0283744	5.60E-05	Down	0.490262
254042	METAP1D	3142	279.4256169	128.7450661	-1.1179471	6.42E-05	Down	0.460749
92312	MEX3A	6124	173.014936	58.60519231	-1.5617962	1.81E-06	Down	0.338729
4237	MFAP2	1121	185.3792399	53.6576026	-1.7886252	6.98E-07	Down	0.289448
146664	MGAT5B	4068	121.0078448	54.08909469	-1.1616909	0.002829	Down	0.446988
284021	MILR1	1488	346.3960449	134.8437204	-1.3611341	8.38E-08	Down	0.389276
126353	MISP	2891	660.0207192	234.050788	-1.4956897	3.56E-14	Down	0.354611
4288	MKI67	11488	11393.68647	6129.92056	-0.8942943	0.002124	Down	0.53801
283078	MKX	3655	47.94604336	10.63629626	-2.1724159	4.99E-05	Down	0.221839
197259	MLKL	2721	498.7122356	157.3918008	-1.6638472	1.79E-16	Down	0.315596
253714	MMS22L	8620	812.6660612	382.3586637	-1.0877361	1.17E-10	Down	0.470499
84057	MND1	945	490.97913	212.5821737	-1.2076411	1.32E-08	Down	0.432976
55329	MNS1	2023	137.1181107	58.57332987	-1.2271033	0.000779	Down	0.427174
55034	MOCOS	3059	564.4670357	264.8383479	-1.0917773	8.04E-09	Down	0.469183
642475	MROH6	3251	349.2288062	179.0688281	-0.9636583	5.55E-05	Down	0.512755
2956	MSH6	4149	2754.692295	1359.700843	-1.0186019	4.00E-16	Down	0.493594
27085	MTBP	3087	481.1547744	238.1841521	-1.0144236	3.46E-06	Down	0.495026
4522	MTHFD1	3466	4964.647756	2764.494678	-0.8446756	1.92E-14	Down	0.556836
25902	MTHFD1L	3101	1172.325214	614.3328274	-0.9322805	5.10E-10	Down	0.524029
4593	MUSK	2466	62.82301634	11.17915581	-2.490482	1.14E-06	Down	0.177947
4602	MYB	3060	50.87419237	18.06163325	-1.4940056	0.006928	Down	0.355025
4605	MYBL2	2774	2679.849514	780.2353121	-1.7801708	4.52E-32	Down	0.291149
80179	MYO19	4302	3515.862452	1798.467051	-0.9671109	1.75E-19	Down	0.511529
55930	MYO5C	6975	814.9354314	438.3145345	-0.8947192	7.58E-07	Down	0.537852
745	MYRF	5756	269.1073367	114.6202615	-1.2313196	2.27E-06	Down	0.425928
259232	NALCN	6867	76.59168967	32.48973338	-1.237204	0.005726	Down	0.424194
89795	NAV3	9774	1410.466831	799.4967481	-0.8190087	2.52E-09	Down	0.566831
4688	NCF2	2202	349.1940301	134.4794898	-1.3766427	8.68E-09	Down	0.385114
55247	NEIL3	2402	407.7358334	237.6643417	-0.7787093	0.000601	Down	0.582888
1E+08	NEMP2	3637	137.7094466	72.61085211	-0.9233704	0.00861	Down	0.527276
54492	NEURL1B	5954	146.7687102	49.07684704	-1.58043	9.81E-06	Down	0.334382
4773	NFATC2	7173	759.8231101	378.3054655	-1.006112	1.08E-06	Down	0.497886
84807	NFKBID	2152	142.2948378	63.50220611	-1.1640047	0.000721	Down	0.446272
64332	NFKBIZ	3928	1122.169456	661.9633721	-0.7614673	3.46E-06	Down	0.589896
374354	NHLRC2	6409	712.9937899	407.0059321	-0.8088397	9.97E-06	Down	0.570841
79815	NIPAL2	4573	222.7318625	110.3334467	-1.0134378	0.000258	Down	0.495364
9241	NOG	1892	1407.759302	700.6760909	-1.0065811	3.89E-12	Down	0.497724
79400	NOX5	2725	80.28508816	32.97128175	-1.2839221	0.008884	Down	0.410678
4883	NPR3	6380	37.79527372	11.17032331	-1.7585349	0.001883	Down	0.295548
7025	NR2F1	3734	165.7291073	91.17641889	-0.8620944	0.008793	Down	0.550153
375387	NRROS	2568	259.2257771	117.6033933	-1.1402795	1.08E-05	Down	0.453672
23225	NUP210	7218	213.9921667	112.5846963	-0.9265473	0.003203	Down	0.526116

4939	OAS2	2775	473.665839	179.637386	-1.398782	6.32E-10	Down	0.379249
4940	OAS3	6657	691.5895512	259.3198472	-1.4151834	5.73E-14	Down	0.374962
8638	OASL	2072	139.2176123	62.08969235	-1.164916	0.001298	Down	0.44599
4998	ORC1	3192	610.0171353	227.376069	-1.4237693	3.92E-14	Down	0.372737
5019	OXCT1	3572	554.9781676	256.9056046	-1.1111927	7.30E-09	Down	0.462911
5026	P2RX5	2277	325.5443454	160.6612977	-1.0188316	1.48E-05	Down	0.493516
132430	PABPC4L	4944	69.77101595	26.41168291	-1.4014516	0.002683	Down	0.378548
29943	PADI1	3847	61.36725554	17.27580173	-1.8287164	0.000174	Down	0.281515
10606	PAICS	3332	9082.5134	5143.957817	-0.8202128	1.75E-15	Down	0.566358
124222	PAQR4	2467	1114.230256	615.9990867	-0.8550473	2.58E-09	Down	0.552847
10038	PARP2	1866	1096.580783	611.249622	-0.8431785	2.69E-08	Down	0.557414
23178	PASK	4692	252.8727498	91.63539279	-1.4644347	2.46E-07	Down	0.362377
84108	PCGF6	2248	553.2417991	307.7917022	-0.8459558	1.59E-05	Down	0.556342
9768	PCLAF	1509	1362.06186	653.669384	-1.0591592	2.46E-13	Down	0.479912
5111	PCNA	1321	4562.123061	2629.909665	-0.7946921	3.08E-13	Down	0.576466
5144	PDE4D	7765	456.4612248	209.2531657	-1.1252429	4.25E-06	Down	0.458425
8622	PDE8B	4114	78.09858954	34.50698175	-1.1784082	0.008084	Down	0.441839
64236	PDLIM2	1825	347.2537762	188.6166345	-0.8805335	0.000125	Down	0.543167
23590	PDSS1	1921	371.5398406	206.2329922	-0.8492418	0.000952	Down	0.555076
375033	PEAR1	4866	459.1455829	171.5997797	-1.419904	1.06E-11	Down	0.373737
5175	PECAM1	6831	214.4225122	80.05569821	-1.4213804	3.22E-06	Down	0.373355
8864	PER2	6342	179.5388293	83.94937425	-1.0967044	0.000491	Down	0.467583
8863	PER3	6321	530.243859	250.0164115	-1.0846332	7.62E-08	Down	0.471512
1911	PHC1	5206	382.1686865	212.411234	-0.8473495	0.000157	Down	0.555805
5253	PHF2	5349	1023.710816	485.645058	-1.075834	3.03E-13	Down	0.474397
9088	PKMYT1	2172	1032.694001	389.8117745	-1.4055633	3.58E-20	Down	0.377471
29941	PKN3	3404	856.6414138	481.3219942	-0.8316891	1.80E-07	Down	0.561871
11187	PKP3	2879	630.5833794	333.592644	-0.9185997	1.08E-05	Down	0.529022
5321	PLA2G4A	2828	153.9591091	81.11550931	-0.9244975	0.005714	Down	0.526864
51316	PLAC8	1403	157.2944989	84.43262795	-0.8975957	0.007169	Down	0.536781
5328	PLAU	2395	11265.32086	5448.628045	-1.0479235	3.32E-14	Down	0.483664
151056	PLB1	5148	107.8369242	31.26479846	-1.7862401	5.38E-06	Down	0.289927
26499	PLEK2	1568	1366.956496	749.3999916	-0.8671595	3.54E-09	Down	0.548225
57664	PLEKHA4	3104	274.6104596	89.0387578	-1.6248812	2.92E-10	Down	0.324237
22874	PLEKHA6	7434	348.5981061	119.6099784	-1.543227	5.71E-11	Down	0.343117
144100	PLEKHA7	5134	271.4868205	131.9774286	-1.0405909	5.29E-05	Down	0.486128
25894	PLEKHG4	4600	404.7534277	166.5042584	-1.2814842	1.20E-08	Down	0.411372
57449	PLEKHG5	4735	124.0236292	62.1716099	-0.9962872	0.007257	Down	0.501288
5362	PLXNA2	11457	1081.368442	572.5966612	-0.917267	8.80E-06	Down	0.529511
56937	PMEPA1	4782	5984.526752	2344.418961	-1.3520068	3.26E-25	Down	0.391747
5422	POLA1	5470	765.1281657	379.6134968	-1.0111701	2.40E-09	Down	0.496144
10714	POLD3	3824	990.7460836	378.514726	-1.3881659	6.99E-19	Down	0.38205
5427	POLE2	1848	966.8869333	361.5233712	-1.4192583	5.66E-18	Down	0.373904
661	POLR3D	1946	338.1684964	191.7276399	-0.8186839	0.000454	Down	0.566959
10622	POLR3G	3285	683.1577943	384.8090629	-0.8280761	4.64E-05	Down	0.56328
5452	POU2F2	6302	4642.709511	2692.683468	-0.7859224	5.27E-14	Down	0.579981
5463	POU6F1	3503	116.406077	54.22429774	-1.102155	0.003318	Down	0.46582

133522	PPARGC1	10642	220.72664	78.82391991	-1.4855554	2.13E-07	Down	0.357111
60490	PPCDC	2235	287.8441211	168.5286057	-0.7722943	0.002635	Down	0.585486
8499	PPFIA2	5540	38.14435497	10.65773294	-1.839569	0.001633	Down	0.279405
11107	PRDM5	5282	272.7887648	111.6603558	-1.2886672	5.26E-07	Down	0.409329
5557	PRIM1	1471	509.7269701	191.9595224	-1.4089226	1.58E-12	Down	0.376593
5583	PRKCH	3868	129.4631285	61.11808451	-1.08287	0.002451	Down	0.472089
163154	PRR22	1407	86.86391759	38.30786335	-1.1811165	0.005849	Down	0.44101
79899	PRR5L	3754	188.3027093	68.8711284	-1.4510825	1.66E-06	Down	0.365747
79056	PRRG4	5538	210.3778406	89.56567915	-1.2319648	3.18E-05	Down	0.425737
64063	PRSS22	1391	158.7935593	64.50520978	-1.2996648	0.000774	Down	0.406221
56952	PRTFDC1	1990	368.2221987	198.9627663	-0.8880781	6.43E-05	Down	0.540333
29893	PSMC3IP	1464	674.0400272	368.9832371	-0.869279	3.66E-06	Down	0.54742
9050	PSTPIP2	3054	71.62898487	26.38997381	-1.4405536	0.001609	Down	0.368426
5743	PTGS2	4507	382.3493449	193.4454438	-0.9829647	0.000118	Down	0.505939
5791	PTPRE	5243	875.0219011	496.4817232	-0.8175785	1.75E-05	Down	0.567393
26108	PYGO1	8290	403.1513423	231.71891	-0.7989457	0.000364	Down	0.574769
81890	QTRT1	1352	604.0040967	317.6622342	-0.9270648	4.37E-07	Down	0.525927
23682	RAB38	1479	293.1633222	154.2099345	-0.9268089	0.000213	Down	0.526021
5888	RAD51	2299	435.3483168	181.9180536	-1.2588814	9.55E-09	Down	0.417868
10635	RAD51AP	2228	667.254912	292.432131	-1.1901362	3.74E-10	Down	0.438261
5892	RAD51D	2429	172.4759821	64.02457983	-1.4296977	6.01E-06	Down	0.371209
8438	RAD54L	3013	492.4863908	243.1733462	-1.0180988	3.71E-07	Down	0.493767
135250	RAET1E	1874	60.56237422	23.59449053	-1.3599717	0.005569	Down	0.38959
5902	RANBP1	1138	3605.90134	2094.559691	-0.7837129	2.16E-10	Down	0.58087
23108	RAP1GAP	6573	187.0590503	58.51583906	-1.6765947	4.67E-08	Down	0.31282
5923	RASGRF1	4860	154.1071416	51.93504657	-1.5691534	2.64E-06	Down	0.337006
55225	RAVER2	4376	276.8082081	148.5570325	-0.8978698	0.000506	Down	0.536679
5932	RBBP8	3264	2578.055456	1219.821006	-1.0796138	3.01E-15	Down	0.473155
5933	RBL1	5916	1612.097157	808.0585725	-0.9964069	9.29E-13	Down	0.501247
389677	RBM12B	5115	476.5693095	280.4077936	-0.7651596	0.000124	Down	0.588388
221662	RBM24	2467	115.5947528	57.26010048	-1.0134738	0.007646	Down	0.495352
1827	RCAN1	2464	597.2078562	342.6363004	-0.8015551	2.56E-05	Down	0.57373
92241	RCSD1	3642	400.1806939	205.7209975	-0.9599625	3.01E-05	Down	0.51407
5649	RELN	11565	59.99104185	11.28652236	-2.4101461	1.67E-06	Down	0.188137
5982	RFC2	1738	1993.061928	961.3622999	-1.0518344	5.83E-16	Down	0.482354
5983	RFC3	2268	756.9390091	408.8829484	-0.8884892	3.30E-07	Down	0.54018
5984	RFC4	1433	1421.648064	747.3592661	-0.9276905	6.33E-10	Down	0.525699
57381	RHOJ	3619	202.2894624	53.88664313	-1.9084215	1.27E-09	Down	0.266384
26150	RIBC2	1405	48.58091904	16.42096859	-1.5648506	0.003181	Down	0.338013
79890	RIN3	3834	811.5660185	444.6259478	-0.8681163	5.78E-08	Down	0.547862
116028	RMI2	1451	357.2178623	198.267417	-0.8493566	0.000162	Down	0.555032
79621	RNASEH2	1689	489.6138679	222.7909585	-1.1359537	3.23E-08	Down	0.455034
55328	RNLS	2385	147.5361392	67.17449378	-1.1350829	0.00069	Down	0.455309
54538	ROBO4	3811	848.1912606	157.1247093	-2.4324795	6.09E-44	Down	0.185247
6240	RRM1	3244	4889.84859	2398.5312	-1.0276386	4.42E-18	Down	0.490512
6241	RRM2	3295	5188.557574	1670.399798	-1.6351401	2.06E-33	Down	0.321939
6242	RTKN	2238	372.1565163	216.7413018	-0.7799354	0.000634	Down	0.582393

219790	RTKN2	4342	263.2458321	128.796411	-1.0313183	0.000132	Down	0.489263
6273	S100A2	970	1189.19956	694.8277615	-0.7752635	8.02E-06	Down	0.584282
140700	SAMD10	2196	499.5405876	272.471725	-0.8744954	9.66E-06	Down	0.545445
23328	SASH1	7301	735.5548521	401.224721	-0.8744224	1.50E-07	Down	0.545472
10371	SEMA3A	5672	417.527795	211.1670797	-0.9834873	9.49E-06	Down	0.505756
10501	SEMA6B	3961	1373.678408	468.7286503	-1.5512194	0.000406	Down	0.341222
29843	SENP1	4800	719.5512024	382.1293676	-0.9130362	6.31E-08	Down	0.531066
8293	SERF1A	1092	254.5821459	5.261274184	-5.5965752	3.12E-33	Down	0.020666
3053	SERPIND1	2237	154.3941213	49.9613805	-1.6277326	1.61E-06	Down	0.323596
56256	SERTAD4	3101	558.9341027	207.9566526	-1.4263954	1.78E-13	Down	0.372059
6422	SFRP1	4482	55.61871594	15.81552158	-1.8142293	0.000403	Down	0.284356
118980	SFXN2	2600	365.7509416	175.3862461	-1.060326	2.93E-06	Down	0.479524
119559	SFXN4	1400	485.004858	278.2432629	-0.8016524	0.00011	Down	0.573692
6446	SGK1	2429	1616.402881	503.5348471	-1.6826233	2.39E-32	Down	0.311516
400745	SH2D5	3705	243.5114989	101.3223953	-1.2650368	2.76E-05	Down	0.416089
23616	SH3BP1	2867	448.2267061	145.6043886	-1.6221748	9.35E-10	Down	0.324845
79628	SH3TC2	26588	425.5029721	162.7086758	-1.386878	1.24E-08	Down	0.382391
53358	SHC3	9768	1059.228539	530.7525819	-0.9969025	1.23E-11	Down	0.501075
79801	SHCBP1	3405	1509.83619	866.9850803	-0.800313	1.20E-06	Down	0.574225
6469	SHH	2033	1126.630624	539.9369237	-1.0611518	1.98E-10	Down	0.479249
387914	SHISA2	2889	820.2077889	439.8860382	-0.8988596	2.77E-05	Down	0.53631
357	SHROOM	5771	467.1191621	196.2023929	-1.251448	8.22E-07	Down	0.420026
59307	SIGIRR	1688	74.76956432	25.99070411	-1.5244554	0.000674	Down	0.347611
6502	SKP2	3491	666.5766415	337.6822234	-0.9811045	1.96E-07	Down	0.506592
10723	SLC12A7	5316	550.467552	318.1142095	-0.7911127	0.000217	Down	0.577898
6563	SLC14A1	3971	83.62548428	29.69015178	-1.4939582	0.001051	Down	0.355037
51296	SLC15A3	2134	156.1246588	76.60435592	-1.0272001	0.001837	Down	0.490661
9120	SLC16A6	3955	47.04066275	16.00846398	-1.5550735	0.004317	Down	0.340311
9194	SLC16A7	11834	573.1536307	251.7662377	-1.1868371	1.58E-10	Down	0.439265
6507	SLC1A3	3701	208.9037719	109.0580613	-0.9377421	0.001101	Down	0.522049
54020	SLC37A1	3306	438.2156743	249.7510452	-0.8111504	0.000393	Down	0.569927
64116	SLC39A8	2917	2030.739232	1020.524109	-0.9926947	9.88E-13	Down	0.502538
8501	SLC43A1	2453	312.0381828	154.7189387	-1.0120728	2.16E-05	Down	0.495833
29015	SLC43A3	2689	10959.3836	4577.37518	-1.2595742	3.21E-35	Down	0.417667
84179	SLC49A3	2003	229.5637384	127.92499	-0.8435967	0.006451	Down	0.557253
6541	SLC7A1	7357	4970.908285	2867.468086	-0.7937321	6.23E-14	Down	0.57685
28234	SLCO1B3	2832	181.1444179	98.72613331	-0.8756364	0.004484	Down	0.545013
146857	SLFN13	8469	655.8322911	266.6686103	-1.2982789	1.65E-13	Down	0.406611
26050	SLITRK5	4461	98.09737813	27.49804703	-1.8348854	6.12E-06	Down	0.280314
6604	SMARCD3	1693	190.3715813	98.28696171	-0.9537462	0.003354	Down	0.51629
56935	SMCO4	979	139.2419546	66.54319205	-1.065231	0.002189	Down	0.477896
6641	SNTB1	4975	83.1790977	33.92043421	-1.2940664	0.003942	Down	0.4078
8470	SORBS2	4511	407.8165461	200.6481717	-1.0232523	3.89E-05	Down	0.492006
10174	SORBS3	2101	1157.612388	600.6859407	-0.9464695	3.13E-11	Down	0.518901
11262	SP140	2264	223.3024648	71.95737372	-1.6337847	1.21E-08	Down	0.322242
147841	SPC24	2003	722.3839641	380.136829	-0.9262471	3.12E-06	Down	0.526225
25803	SPDEF	1914	1498.13771	528.585075	-1.5029626	0.000142	Down	0.352828

54908	SPDL1	2700	1874.776307	1098.023363	-0.7718097	8.80E-06	Down	0.585682
90853	SPOCD1	2518	242.4524972	58.99714601	-2.038985	7.55E-12	Down	0.243335
6695	SPOCK1	4841	105.7598846	32.48448937	-1.7029696	7.18E-05	Down	0.307153
6712	SPTBN2	10620	386.4932332	217.4335065	-0.8298689	0.000147	Down	0.56258
6768	ST14	3319	219.1632853	86.91270625	-1.3343671	3.53E-05	Down	0.396566
1.01E+08	ST20-MTI	2282	15.75057111	3.807860483	-2.0483516	0.00108	Down	0.24176
55620	STAP2	1471	629.7719528	341.2655296	-0.8839348	7.34E-07	Down	0.541887
6776	STAT5A	4265	471.8545597	186.3463423	-1.3403557	7.20E-11	Down	0.394923
261729	STEAP2	6819	274.3691808	139.3769894	-0.977126	0.000176	Down	0.507991
26032	SUSD5	5005	159.4103932	82.43952791	-0.9513375	0.00427	Down	0.517153
91683	SYT12	3499	77.67422727	28.43757387	-1.4496376	0.001907	Down	0.366113
23329	TBC1D30	7678	107.9176184	49.52023197	-1.1238404	0.003981	Down	0.458871
6899	TBX1	1977	92.76203474	32.6186936	-1.5078355	0.000337	Down	0.351638
6916	TBXAS1	2284	283.1609296	149.2085474	-0.924292	0.001228	Down	0.526939
6920	TCEA3	1750	312.4748	171.5157686	-0.8653986	0.002028	Down	0.548895
6941	TCF19	3219	3118.644225	1381.262591	-1.1749314	1.65E-23	Down	0.442905
6949	TCOF1	4914	3251.576836	1867.501539	-0.8000301	6.03E-12	Down	0.574337
7015	TERT	4018	287.4559304	141.0932639	-1.0266917	0.000739	Down	0.490834
7023	TFAP4	2147	433.2993413	184.2361534	-1.2338078	9.67E-09	Down	0.425194
7042	TGFB2	5934	1787.531407	769.2269035	-1.2164875	1.70E-14	Down	0.430329
1.01E+08	TGFBR3L	1256	196.6055447	95.39016443	-1.0433916	0.000385	Down	0.485186
7052	TGM2	5033	9041.172983	4699.208159	-0.9440923	4.97E-17	Down	0.519756
7056	THBD	4048	2472.103899	1020.092136	-1.2770399	7.03E-26	Down	0.412641
92610	TIFA	3090	460.7797828	261.3252503	-0.8182309	0.000119	Down	0.567137
91151	TIGD7	3435	248.5315261	125.8547655	-0.981669	0.000395	Down	0.506394
54962	TIPIN	1295	373.6012422	168.1812373	-1.1514825	4.21E-06	Down	0.450162
7098	TLR3	3057	136.7005851	32.26771747	-2.082856	1.40E-08	Down	0.236047
10333	TLR6	5891	260.1900852	141.6468676	-0.8772673	0.001043	Down	0.544398
79905	TMC7	3882	279.4811976	138.2496285	-1.0154756	5.10E-05	Down	0.494665
255104	TMCO4	3071	547.4403252	239.999333	-1.1896713	7.80E-10	Down	0.438403
202915	TMEM184	6293	174.0168254	79.57258588	-1.1288834	0.000337	Down	0.457269
399474	TMEM20(	2500	480.5436806	134.5924408	-1.8360702	4.58E-19	Down	0.280084
137835	TMEM71	2029	139.4442388	26.98162398	-2.3696392	8.36E-10	Down	0.193494
7112	TMPO	4038	9371.306416	4740.435708	-0.9832305	5.18E-12	Down	0.505846
3371	TNC	8605	5753.759583	2446.889589	-1.2335559	0.003021	Down	0.425268
8764	TNFRSF14	3498	222.5388445	127.2928447	-0.8059059	0.007335	Down	0.572003
8741	TNFSF13	2274	401.6102468	216.2593942	-0.8930333	6.21E-05	Down	0.538481
9966	TNFSF15	6639	938.5150107	286.2303235	-1.7132033	3.06E-18	Down	0.304982
79931	TNIP3	2416	48.77032226	17.88932671	-1.4469044	0.006496	Down	0.366808
23371	TNS2	4738	1013.831054	538.3294142	-0.9132561	1.16E-09	Down	0.530985
4796	TONSL	4519	1667.158836	977.8770939	-0.7696665	1.51E-09	Down	0.586553
11073	TOPBP1	5378	1816.172948	917.9227081	-0.984457	1.13E-12	Down	0.505416
9760	TOX	4131	121.4863094	53.57206246	-1.181241	0.002226	Down	0.440972
7161	TP73	5022	251.7448479	57.53239128	-2.1295159	1.92E-14	Down	0.228535
7164	TPD52L1	2250	89.67388737	39.3729274	-1.187484	0.004756	Down	0.439068
129293	TRABD2A	2172	106.4581293	51.04521585	-1.0604385	0.008855	Down	0.479486
7188	TRAF5	4001	548.3560187	301.0117429	-0.8652931	5.15E-06	Down	0.548935

57761	TRIB3	2475	508.5568696	294.8538389	-0.7864091	9.99E-05	Down	0.579785
9830	TRIM14	4629	1074.831435	585.7963818	-0.8756392	2.91E-09	Down	0.545012
205860	TRIML2	1655	183.9841822	53.33264583	-1.7864909	8.11E-08	Down	0.289876
9319	TRIP13	2378	1058.859396	579.3935959	-0.8698954	2.32E-07	Down	0.547187
10103	TSPAN1	1639	239.4769168	120.9758249	-0.9851678	0.005413	Down	0.505167
7298	TYMS	1598	5152.418402	2093.055956	-1.2996389	7.01E-23	Down	0.406228
494514	TYMSOS	860	38.84791951	13.6787564	-1.5059003	0.008649	Down	0.35211
7301	TYRO3	4001	464.6438132	202.5257073	-1.1980202	1.29E-07	Down	0.435873
55148	UBR7	3709	2400.917272	1391.89706	-0.7865332	1.94E-09	Down	0.579736
91544	UBXN11	1672	271.4960934	153.1888084	-0.8256205	0.001618	Down	0.564239
90226	UCN2	1546	395.7123888	176.1741994	-1.1674496	1.14E-07	Down	0.445208
29128	UHRF1	3981	3903.53532	1205.575213	-1.6950597	2.12E-51	Down	0.308842
7374	UNG	2102	1672.169543	643.0942708	-1.378619	1.10E-19	Down	0.384587
9816	URB2	5641	782.0106224	454.3431921	-0.7834057	1.79E-05	Down	0.580994
7398	USP1	3685	3251.435805	1457.847099	-1.1572375	4.28E-20	Down	0.44837
7421	VDR	4700	208.6795891	109.247715	-0.9336864	0.001747	Down	0.523519
8876	VNN1	3844	533.3891821	175.1333637	-1.6067346	1.82E-16	Down	0.328341
340547	VSIG1	3145	165.3789204	61.51319252	-1.4268076	1.50E-05	Down	0.371953
54621	VSIG10	5003	651.9842811	370.2262245	-0.8164301	4.21E-06	Down	0.567845
11169	WDHD1	6044	1896.338326	1038.151118	-0.8691999	2.11E-10	Down	0.54745
79968	WDR76	4037	839.9484074	284.556909	-1.5615835	1.23E-18	Down	0.338779
197335	WDR90	5541	550.7565632	305.6514506	-0.8495274	1.23E-05	Down	0.554967
7490	WT1	2438	28.76899491	8.150974079	-1.8194704	0.0028	Down	0.283325
7516	XRCC2	3094	582.2367191	294.213004	-0.9847448	1.92E-07	Down	0.505315
80149	ZC3H12A	2725	2521.492042	1303.662847	-0.9517069	1.76E-14	Down	0.51702
195828	ZNF367	3714	927.5152729	269.7092337	-1.7819661	2.37E-28	Down	0.290787
55205	ZNF532	6089	930.476007	386.1585242	-1.2687757	2.62E-15	Down	0.415012
114991	ZNF618	9352	261.0071657	83.21961882	-1.6490938	5.80E-10	Down	0.31884
115509	ZNF689	3565	344.1511269	169.9107777	-1.0182649	8.83E-06	Down	0.49371
91752	ZNF804A	4700	81.9150415	31.15311189	-1.3947521	0.001286	Down	0.38031
152485	ZNF827	7805	733.3376439	375.3384256	-0.9662856	1.57E-08	Down	0.511822
11130	ZWINT	1703	2636.04569	1452.348157	-0.859988	1.85E-11	Down	0.550957

Supplemental Table S4

ARE Type	Gene Symbol	Ensembl Gene ID	Ensembl Transcript ID	Cluster	Sequence	Start	End
3'UTR	ADAM28	ENSG00000042980	ENST00000265769	1	ACTTATTTAATTT	4600	4613
3'UTR	ADAMTS3	ENSG00000156140	ENST00000286657	1	AGATATTTATTTT	1122	1135
3'UTR	ADAMTS9	ENSG00000163638	ENST00000498707	1	AATTATTTATTGT	240	253
3'UTR	ADCY7	ENSG00000121281	ENST00000254235	1	GAATATTTATAGA	2521	2534
3'UTR	AEN	ENSG00000181026	ENST00000332810	2	TTATTTATTTATT	929	942
3'UTR	AKAP12	ENSG00000131016	ENST00000402676	1	ACATATTTATATG	337	350
3'UTR	ALDH1A3	ENSG00000184254	ENST00000329841	1	CATTATTTATAAT	1607	1620
3'UTR	ALG10B	ENSG00000175548	ENST00000308742	1	ACTAATTTATTCT	335	348
3'UTR	ANKRD6	ENSG00000135299	ENST00000369408	1	TAAAATTTATTGA	596	609
3'UTR	ARMCX4	ENSG00000196440	ENST00000433011	1	GTTAATTTATTTT	3159	3172
3'UTR	AS3MT	ENSG00000214435	ENST00000369880	3	ATTTATTTATTTA	491	504
3'UTR	B3GALT5	ENSG00000183778	ENST00000380620	1	AGTAATTTATTAA	5929	5942
3'UTR	BARD1	ENSG00000138376	ENST00000619009	1	TTTTATTTATAAC	1680	1693
3'UTR	BCAT1	ENSG00000060982	ENST00000261192	1	AATTATTTATTTG	2139	2152
3'UTR	BCL2	ENSG00000171791	ENST00000398117	3	ATTTATTTATTTA	251	264
3'UTR	BEND3	ENSG00000178409	ENST00000369042	1	GAAAATTTATTGA	1422	1435
3'UTR	BMPER	ENSG00000164619	ENST00000297161	1	TTTAATTTAATAA	277	290
3'UTR	BMPR1B	ENSG00000138696	ENST00000515059	2	ATATTTATTTATA	3094	3107
3'UTR	CACNB4	ENSG00000182389	ENST00000539935	1	ATATATTTATTAC	2076	2089
3'UTR	CCDC190	ENSG00000185860	ENST00000367912	1	CTTAATTTATTTG	104	117
3'UTR	CCNE2	ENSG00000175305	ENST00000520509	1	GTAAATTTATTTG	1771	1784
3'UTR	CD274	ENSG00000120217	ENST00000381573	1	TTTTATTTATTTT	1683	1696
3'UTR	CDC25A	ENSG00000164045	ENST00000302506	1	TTTAATTTATTCA	427	440
3'UTR	CDC6	ENSG00000094804	ENST00000209728	1	ATATATTTATTTT	896	909
3'UTR	CHML	ENSG00000203668	ENST00000366553	1	TCTTATTTAATAC	750	763
3'UTR	CHST3	ENSG00000122863	ENST00000373115	2	TAATTTATTTATT	2576	2589
3'UTR	CIITA	ENSG00000179583	ENST00000324288	1	TTTTATTTAATTT	2684	2697
3'UTR	CLMP	ENSG00000166250	ENST00000448775	1	AAATATTTATTAT	3544	3557
3'UTR	COBLL1	ENSG00000082438	ENST00000392717	2	TTATTTATTTATA	2751	2764
3'UTR	COL27A1	ENSG00000196739	ENST00000356083	1	TATTATTTAAACA	452	465
3'UTR	CSF2	ENSG00000164400	ENST00000296871	5	ATTTATTTATTTATTT/	228	249
3'UTR	CSGALNACT1	ENSG00000147408	ENST00000397998	1	TTTTATTTAAAAA	1606	1619
3'UTR	CX3CL1	ENSG00000006210	ENST00000006053	1	AATTATTTATTAA	295	308
3'UTR	CXCL1	ENSG00000163739	ENST00000395761	3	ATTTATTTATTTA	145	158
3'UTR	CXCL2	ENSG00000081041	ENST00000508487	5	ATTTATTTATTTATTT/	145	166
3'UTR	CXCL3	ENSG00000163734	ENST00000296026	1	TGTTATTTAAAGA	206	219
3'UTR	CXCL8	ENSG00000169429	ENST00000307407	2	GTATTTATTTAAG	694	707
3'UTR	DIXDC1	ENSG00000150764	ENST00000440460	1	TATTATTTATATA	1220	1233
3'UTR	DNA2	ENSG00000138346	ENST00000358410	1	TTTTATTTAATAA	944	957
3'UTR	DOCK10	ENSG00000135905	ENST00000258390	1	TTTAATTTATTTG	76	89
3'UTR	DSC2	ENSG00000134755	ENST00000280904	1	AGTTATTTATAAA	5716	5729
3'UTR	DTL	ENSG00000143476	ENST00000542077	1	AGATATTTAATAC	1068	1081
3'UTR	E2F1	ENSG00000101412	ENST00000343380	2	TTATTTATTTATC	590	603
3'UTR	E2F8	ENSG00000129173	ENST00000527884	1	AATTATTTATAAA	533	546
3'UTR	EDN1	ENSG00000078401	ENST00000379375	1	AGATATTTATATT	949	962

3'UTR	EDN2	ENSG00000127129	ENST00000372587	1	AATTATTTATTTT	558	571
3'UTR	EOGT	ENSG00000163378	ENST00000383701	1	ATTAATTTATATT	1082	1095
3'UTR	EVI2A	ENSG00000126860	ENST00000462804	1	AATTATTTAAACT	159	172
3'UTR	EXOSC9	ENSG00000123737	ENST00000513654	2	GTATTTATTTACT	1563	1576
3'UTR	EZH2	ENSG00000106462	ENST00000492143	1	AATAATTTATAGT	2369	2382
3'UTR	F2RL3	ENSG00000127533	ENST00000248076	1	ATTAATTTAATTC	478	491
3'UTR	FAM81A	ENSG00000157470	ENST00000288228	1	ATATATTTAATAA	744	757
3'UTR	FAS	ENSG00000026103	ENST00000355740	1	TAATATTTATATT	1171	1184
3'UTR	FRMD4A	ENSG00000151474	ENST00000357447	1	TTTAATTTATTCT	3233	3246
3'UTR	FST	ENSG00000134363	ENST00000256759	2	ACATTTATTTATA	282	295
3'UTR	GALNT3	ENSG00000115339	ENST00000392701	1	TCTTATTTATATC	124	137
3'UTR	GDF15	ENSG00000130513	ENST00000252809	2	GTATTTATTTAAA	225	238
3'UTR	GJA1	ENSG00000152661	ENST00000282561	1	ACTAATTTATTTG	1440	1453
3'UTR	GLIPR1	ENSG00000139278	ENST00000266659	1	TATAATTTATAAA	676	689
3'UTR	GPR3	ENSG00000181773	ENST00000374024	3	ATTTATTTATTTA	442	455
3'UTR	GSAP	ENSG00000186088	ENST00000257626	1	ACATATTTATTTT	531	544
3'UTR	HS3ST1	ENSG00000002587	ENST00000002596	1	TATAATTTATTTG	88	101
3'UTR	IFI44L	ENSG00000137959	ENST00000370751	1	ATTAATTTATTTT	3684	3697
3'UTR	IFIT1	ENSG00000185745	ENST00000546318	1	AATTATTTAATAT	2257	2270
3'UTR	IKZF2	ENSG00000030419	ENST00000457361	1	ACATATTTATATG	6735	6748
3'UTR	IL11	ENSG00000095752	ENST00000264563	3	ATTTATTTATTTA	43	56
3'UTR	IL1A	ENSG00000115008	ENST00000263339	1	TATTATTTATATA	610	623
3'UTR	IL1B	ENSG00000125538	ENST00000263341	3	ATTTATTTATTTA	345	358
3'UTR	IL6	ENSG00000136244	ENST00000404625	1	TTTAATTTATTAA	134	147
3'UTR	IL7R	ENSG00000168685	ENST00000303115	1	TCATATTTAATTA	3087	3100
3'UTR	INHBA	ENSG00000122641	ENST00000242208	2	ATATTTATTTAAG	3521	3534
3'UTR	ITPKB	ENSG00000143772	ENST00000429204	1	TTATATTTATTTT	2560	2573
3'UTR	JPH1	ENSG00000104369	ENST00000342232	1	ACTAATTTAATTC	873	886
3'UTR	KDELC2	ENSG00000178202	ENST00000323468	1	AAATATTTATATT	2598	2611
3'UTR	KIAA1211	ENSG00000109265	ENST00000504228	2	TTATTTATTTATT	86	99
3'UTR	KLHL3	ENSG00000146021	ENST00000309755	1	GTATATTTATTGT	1758	1771
3'UTR	KLHL42	ENSG00000087448	ENST00000381271	1	TCTAATTTAATCT	950	963
3'UTR	LRRCC1	ENSG00000133739	ENST00000360375	1	TGTTATTTAATTG	379	392
3'UTR	LSM11	ENSG00000155858	ENST00000286307	2	TTATTTATTTAGA	4807	4820
3'UTR	MANEA	ENSG00000172469	ENST00000358812	2	TTTAATTTATTTAAAA	1625	1642
3'UTR	MAPK13	ENSG00000156711	ENST00000373766	1	TTATATTTATATA	5023	5036
3'UTR	MCM10	ENSG00000065328	ENST00000378714	2	AGATTTATTTATC	1561	1574
3'UTR	MCM6	ENSG00000076003	ENST00000264156	1	AGTAATTTATTTT	653	666
3'UTR	MEX3A	ENSG00000254726	ENST00000532414	1	TATAATTTAATAT	2178	2191
3'UTR	MKX	ENSG00000150051	ENST00000375790	1	TTTAATTTATTTT	2115	2128
3'UTR	MOB3B	ENSG00000120162	ENST00000262244	1	GTTTATTTATAGA	3617	3630
3'UTR	MOCOS	ENSG00000075643	ENST00000261326	1	GAATATTTATATT	540	553
3'UTR	MTBP	ENSG00000172167	ENST00000305949	1	TCTTATTTAATTA	259	272
3'UTR	MTHFD1L	ENSG00000120254	ENST00000367321	1	ACTAATTTATTTC	219	232
3'UTR	MYB	ENSG00000118513	ENST00000442647	1	AGTAATTTAATTT	613	626
3'UTR	NEMP2	ENSG00000189362	ENST00000409150	1	TATTATTTAAATA	3655	3668
3'UTR	NOL4L	ENSG00000197183	ENST00000359676	1	AGTAATTTATTTT	424	437

3'UTR	NPR3	ENSG00000113389	ENST00000265074	1	TTTTATTTAATCA	3683	3696
3'UTR	NRROS	ENSG00000174004	ENST00000328557	1	AAATATTTATTAA	214	227
3'UTR	NUP210	ENSG00000132182	ENST00000254508	1	GTTTATTTAATAA	1413	1426
3'UTR	OAS2	ENSG00000111335	ENST00000342315	1	TCATATTTATATG	520	533
3'UTR	PABPC4L	ENSG00000254535	ENST00000421491	2	TTTAATTTATTTAAAT	3159	3176
3'UTR	PCDH7	ENSG00000169851	ENST00000511884	1	CATTATTTAAACA	1402	1415
3'UTR	PDE4B	ENSG00000184588	ENST00000329654	1	ATTAATTTATATA	1007	1020
3'UTR	PDGFB	ENSG00000100311	ENST00000331163	2	AAATTTATTTATA	1629	1642
3'UTR	PER3	ENSG00000049246	ENST00000613533	1	ACTAATTTATTTT	1269	1282
3'UTR	PHF2	ENSG00000197724	ENST00000359246	1	TGTAATTTATTAA	1434	1447
3'UTR	PLA2G4A	ENSG00000116711	ENST00000367466	1	TTTTATTTATATA	414	427
3'UTR	PLAU	ENSG00000122861	ENST00000446342	1	TTTTATTTATATT	870	883
3'UTR	PLEKHA6	ENSG00000143850	ENST00000272203	1	GGAAATTTATTAT	3633	3646
3'UTR	PLXNA2	ENSG00000076356	ENST00000367033	1	CTTTATTTAATTT	352	365
3'UTR	PMEPA1	ENSG00000124225	ENST00000265626	1	AGTTATTTATATA	586	599
3'UTR	POLD3	ENSG00000077514	ENST00000263681	2	ACATTTATTTATA	1028	1041
3'UTR	POU6F1	ENSG00000184271	ENST00000389243	1	TTTAATTTAATCT	802	815
3'UTR	PPARGC1B	ENSG00000155846	ENST00000309241	1	CATTATTTATTAG	6698	6711
3'UTR	PPFIA2	ENSG00000139220	ENST00000549396	1	AATTATTTAAAAA	761	774
3'UTR	PRDM1	ENSG00000057657	ENST00000369096	1	CATAATTTATTAT	360	373
3'UTR	PRDM5	ENSG00000138738	ENST00000264808	1	CATTATTTATTCT	666	679
3'UTR	PRKCH	ENSG00000027075	ENST00000332981	1	AGATATTTATTAA	625	638
3'UTR	PRRG4	ENSG00000135378	ENST00000257836	1	TGATATTTATAGA	2827	2840
3'UTR	PRRX1	ENSG00000116132	ENST00000239461	1	ATATATTTATATA	954	967
3'UTR	PRSS22	ENSG00000005001	ENST00000161006	1	AATTATTTATTCT	347	360
3'UTR	PSMC3IP	ENSG00000131470	ENST00000253789	1	CAATATTTATTAT	271	284
3'UTR	PSTPIP2	ENSG00000152229	ENST00000409746	1	TATTATTTATTGT	1771	1784
3'UTR	PTGS2	ENSG00000073756	ENST00000367468	3	ATTTATTTATTTA	15	28
3'UTR	PTPN22	ENSG00000134242	ENST00000359785	1	TTATATTTATTAG	542	555
3'UTR	RAD54L	ENSG00000085999	ENST00000442598	1	CAAAATTTATTTT	128	141
3'UTR	RAPGEF5	ENSG00000136237	ENST00000344041	1	TAATATTTATTTG	3599	3612
3'UTR	RBBP8	ENSG00000101773	ENST00000327155	1	AGTTATTTATAGT	46	59
3'UTR	RBL1	ENSG00000080839	ENST00000373664	1	CTTAATTTAATAT	701	714
3'UTR	RTKN2	ENSG00000182010	ENST00000373789	1	ACTTATTTAAAAA	805	818
3'UTR	SAMD10	ENSG00000130590	ENST00000369886	1	CATTATTTATACA	591	604
3'UTR	SEMA3A	ENSG00000075213	ENST00000265362	1	GTTTATTTATATA	5444	5457
3'UTR	SERF1A	ENSG00000172058	ENST00000354833	2	TAATTTATTTATT	378	391
3'UTR	SERTAD4	ENSG00000082497	ENST00000367012	1	ATATATTTATTAA	1735	1748
3'UTR	SFXN2	ENSG00000156398	ENST00000369893	1	TCTTATTTATTGA	3494	3507
3'UTR	SGK1	ENSG00000118515	ENST00000367858	1	TAAAATTTATTGA	601	614
3'UTR	SH3BP1	ENSG00000100092	ENST00000451997	2	TTATTTATTTATT	2884	2897
3'UTR	SHC3	ENSG00000148082	ENST00000375835	2	TAATTTATTTATA	5271	5284
3'UTR	SHH	ENSG00000164690	ENST00000297261	1	GATAATTTATTAT	339	352
3'UTR	SLC16A6	ENSG00000108932	ENST00000327268	1	TAATATTTATAAC	1859	1872
3'UTR	SLC1A3	ENSG00000079215	ENST00000265113	1	AAATATTTATTTG	1355	1368
3'UTR	SLC39A8	ENSG00000138821	ENST00000356736	1	GAATATTTAATAT	1284	1297
3'UTR	SLITRK5	ENSG00000165300	ENST00000325089	2	TTATTTATTTAAA	#####	11697

3'UTR	SMCO4	ENSG00000166002	ENST00000298966	1	AAATATTTATTTT	498	511
3'UTR	SNTB1	ENSG00000172164	ENST00000395601	1	TTTAATTTAATTA	2178	2191
3'UTR	SORCS3	ENSG00000156395	ENST00000369701	1	AATAATTTAATGT	570	583
3'UTR	SOST	ENSG00000167941	ENST00000301691	1	ACATATTTATTTT	1466	1479
3'UTR	SPC24	ENSG00000161888	ENST00000592540	1	ACTTATTTAAATG	1568	1581
3'UTR	STEAP2	ENSG00000157214	ENST00000287908	2	AGATTTATTTATG	3607	3620
3'UTR	STYK1	ENSG00000060140	ENST00000075503	1	TAATATTTATTAA	947	960
3'UTR	SUSD5	ENSG00000173705	ENST00000309558	1	ATATATTTATAAA	2202	2215
3'UTR	TGFB2	ENSG00000092969	ENST00000366930	1	AGTTATTTATTGT	465	478
3'UTR	TGM2	ENSG00000198959	ENST00000361475	1	TTTTATTTAATCC	2455	2468
3'UTR	THBD	ENSG00000178726	ENST00000377103	2	TTATTTATTTATT	1725	1738
3'UTR	TIPIN	ENSG00000075131	ENST00000261881	2	GTATTTATTTATA	168	181
3'UTR	TLR6	ENSG00000174130	ENST00000436693	1	GCTTATTTAATTT	494	507
3'UTR	TMC7	ENSG00000170537	ENST00000421369	1	TATTATTTAAAGT	1218	1231
3'UTR	TMEM184A	ENSG00000164855	ENST00000297477	1	TCTTATTTATATT	692	705
3'UTR	TNFSF15	ENSG00000181634	ENST00000374045	1	TAATATTTATTGA	1934	1947
3'UTR	TOPBP1	ENSG00000163781	ENST00000260810	1	AGATATTTATTTT	267	280
3'UTR	TOX	ENSG00000198846	ENST00000361421	1	TTTTATTTATTAT	2070	2083
3'UTR	TRAF5	ENSG00000082512	ENST00000336184	1	TATTATTTATATT	212	225
3'UTR	TYRO3	ENSG00000092445	ENST00000263798	1	TCAAATTTATTGT	4459	4472
3'UTR	USP43	ENSG00000154914	ENST00000285199	1	GAATATTTAATAT	557	570
3'UTR	VDR	ENSG00000111424	ENST00000395324	1	TTAAATTTATTAG	1631	1644
3'UTR	VEPH1	ENSG00000197415	ENST00000362010	1	ACTTATTTAATTA	604	617
3'UTR	VNN1	ENSG00000112299	ENST00000367928	1	TTTTATTTAAAAA	178	191
3'UTR	WDHD1	ENSG00000198554	ENST00000360586	1	GTTTATTTATAGT	1514	1527
3'UTR	WDR76	ENSG00000092470	ENST00000263795	1	GTTAATTTATAAA	1753	1766
3'UTR	ZNF367	ENSG00000165244	ENST00000375256	1	GATTATTTATAAG	377	390
3'UTR	ZNF532	ENSG00000074657	ENST00000336078	1	TGTAATTTATTAA	871	884
3'UTR	ZNF618	ENSG00000157657	ENST00000288466	1	ATTAATTTATACT	5688	5701
3'UTR	ZNF804A	ENSG00000170396	ENST00000302277	1	ATATATTTATAGT	277	290
3'UTR	ZNF827	ENSG00000151612	ENST00000508784	1	ACTTATTTAAAAG	2190	2203
